# Safety of psychotropic medications in pregnancy: an umbrella review

**DOI:** 10.1038/s41380-024-02697-0

**Published:** 2024-09-12

**Authors:** Nicholas Fabiano, Stanley Wong, Arnav Gupta, Jason Tran, Nishaant Bhambra, Kevin K. Min, Elena Dragioti, Corrado Barbui, Jess G. Fiedorowicz, Corentin J. Gosling, Samuele Cortese, Jasmine Gandhi, Gayatri Saraf, Risa Shorr, Simone N. Vigod, Benicio N. Frey, Richard Delorme, Marco Solmi

**Affiliations:** 1 SCIENCES Lab, Department of Psychiatry, University of Ottawa, Ottawa, ON Canada; 2 Department of Psychiatry, University of Toronto, Toronto, ON Canada; 3 Department of Medicine, University of Calgary, Calgary, AB Canada; 4 College of Public Health, Kent State University, Kent, OH US; 5 Department of Family Medicine, University of Ottawa, Ottawa, ON Canada; 6 Faculty of Medicine, University of Ottawa, Ottawa, ON Canada; 7 Research Laboratory Psychology of Patients, Families & Health Professionals, Department of Nursing, School of Health Sciences, University of Ioannina, Ioannina, Greece; 8 WHO Collaborating Centre for Research and Training in Mental Health and Service Evaluation, Department of Neuroscience, Biomedicine and Movement Sciences, Section of Psychiatry, University of Verona, Verona, Italy; 9 Department of Psychiatry, University of Ottawa, Ottawa, ON Canada; 10 Department of Mental Health, The Ottawa Hospital, Ottawa, ON Canada; 11 Ottawa Hospital Research Institute (OHRI) Clinical Epidemiology Program, University of Ottawa, Ottawa, ON Canada; 12 School of Epidemiology and Public Health, Faculty of Medicine, University of Ottawa, Ottawa, ON Canada; 13 DysCo Laboratory, Université Paris Nanterre, F9200 Nanterre, France; 14 Laboratory of Psychopathology and Health Process, Université Paris Cité, F92000 Paris, France; 15 Centre for Innovation in Mental Health, School of Psychology, Faculty of Environmental and Life Sciences, University of Southampton, Southampton, UK; 16 Clinical and Experimental Sciences (CNS and Psychiatry), Faculty of Medicine, University of Southampton, Southampton, UK; 17 Solent NHS Trust, Southampton, UK; 18 Hassenfeld Children’s Hospital at NYU Langone, New York University Child Study Center, New York, NY USA; 19 DiMePRe-J-Department of Precision and Regenerative Medicine-Jonic Area, University of Bari “Aldo Moro”, Bari, Italy; 20 The Royal’s Institute of Mental Health Research, Ottawa, ON Canada; 21 Library Services, The Ottawa Hospital, Ottawa, ON Canada; 22 Department of Psychiatry, Women’s, College Hospital and University of Toronto, Toronto, ON Canada; 23 Department of Psychiatry and Behavioural Neurosciences, McMaster University, Hamilton, ON Canada; 24 Women’s Health Concerns Clinic, St. Joseph’s Healthcare Hamilton, Hamilton, ON Canada; 25 Child and Adolescent Psychiatry Department, Robert Debré Hospital, APHP, University of Paris Cité, Paris, France; 26 Department of Child and Adolescent Psychiatry, Charité Universitätsmedizin, Berlin, Germany

**Keywords:** Psychiatric disorders, Psychology

## Abstract

Weighing risks and benefits of the use of psychotropic medications during pregnancy remains a challenge worldwide. We systematically assessed the strength of associations between psychotropic medication use in pregnant people with mental disorders and various adverse health outcomes in both pregnant people and foetuses. Systematic reviews with meta-analyses of observational studies investigating the association between exposure to psychotropic medication in pregnancy and any adverse health outcomes were included. Credibility was graded into convincing, highly suggestive, suggestive, weak or not significant. Quality of the meta-analyses and of individual studies were assessed with A Measurement Tool to Assess Systematic Reviews 2 (AMSTAR 2) the Newcastle-Ottawa Scale (NOS), respectively. We considered 21 meta-analyses encompassing 17,290,755 participants (AMSTAR 2 high = 1, low = 12, or critically low = 8). Evidence was suggestive for: (1) preterm birth in pregnant people with either any mental disorder (equivalent odds ratio 1.62 (95% confidence interval 1.24–2.12) or depression (1.65 [1.34–2.02]) receiving antidepressants during any trimester of pregnancy; (2) small for gestational age for pregnant people with depression receiving a SSRI during any trimester of pregnancy (1.50 [1.19–1.90]); and (3) major congenital malformation (1.24 [1.09–1.40]) or cardiac malformations (1.28 [1.11–1.47]) in babies for pregnant people with depression or anxiety receiving paroxetine during first trimester of pregnancy. Additional associations were supported by weak evidence, or were not statistically significant. This umbrella review found no convincing or highly suggestive level of evidence of adverse health outcomes associated with psychotropic medication use in pregnant people with mental disorders.

## Introduction

Weighing risks and benefits of the use of psychotropic medications in pregnancy remains a challenge worldwide, generating uncertainty in many healthcare professionals with and without specialised training [1]. Although mental disorders are prevalent in pregnant people [2, 3] and psychotropic medications are frequently used [4–7], concerns for maternal and foetal safety as well as long-term neurodevelopmental effects have been raised [8–12].

Existing evidence on the safety of psychotropic medications during pregnancy is still inconsistent, leading to uncertainty and insufficient guidance for clinicians [13]. As our understanding of the impact of psychotropic medications during pregnancy expands, so does our knowledge about the effects of untreated psychiatric illness on foetal development and obstetric outcomes. However, it remains challenging to disseminate this information to those providing care and with evidence that can easily be applied. As such, the National Institute for Health Care Excellence (NICE) has suggested that decisions should be patient-specific [14]. There are a lack of randomised controlled trials (RCTs) in this area due to ethical considerations and challenges in recruiting pregnant people for clinical trials [15, 16]. Further, RCTs are not well suited to detect rare outcomes. Therefore, we must turn to observational studies to provide information on various adverse health outcomes associated with psychotropic medication use during pregnancy [17, 18]. Observational studies include larger and more representative populations, with longer follow-up times, providing naturalistic information on long-term and infrequent health consequences, but are limited by the confounding factors resulting from these studies.

Potential risks associated with certain psychotropic medications for pregnant people may be overestimated due to confounding by indication [19, 20]. This is primarily due to a lack of consideration given to risk associated with the underlying mental disorder when comparing people on psychotropic medication versus those who are not.

In this umbrella review, we aim to quantify the strength of associations from meta-analyses of observational studies on the association of the use of psychotropic drug during pregnancy and various adverse health outcomes in both pregnant people and foetuses, while controlling for underlying maternal psychiatric conditions (i.e., confounding by indication).

## Methods

This study followed an a priori protocol available at: https://osf.io/8vt4g/. Protocol amendments with their rationale are available in supplementary material 1. We adhered to the Preferred Reporting Items for Overviews of Reviews (PRIOR) and PRISMA 2020 guidelines (adapting PRISMA to the abstract of an umbrella review; Supplementary Material 1, Supplementary eTables 1and 2) guidelines [21, 22].

### Search strategy and inclusion criteria

We searched PubMed, Scopus, and PsycINFO for systematic reviews (as of 04/05/2023) with meta-analysis of observational studies investigating the association between exposure to psychotropic medication in pregnancy and any adverse health outcome (in pregnant persons or foetuses). A librarian (RS) was involved in optimising the search strategy (Supplementary eTable 3, Supplementary Material 1). Psychotropic medications among those in category N05, N06, N07 of the Anatomical Therapeutic Chemical (ATC) World Health Organization (WHO) database were included as listed in Supplementary eTable 4 (Supplementary Material 1). The inclusion and exclusion criteria are available in the eMethods.

### Study screening, data extraction and quality assessment

Study screening was conducted in Covidence in two stages [23]. In the first stage, blinded pairs from among five investigators (SW, AG, JT, NB, KM) independently screened the titles and abstracts and included eligible systematic reviews. A sixth reviewer (NF) was available for discrepancies to reach consensus. The full texts of eligible articles were retrieved and the same five investigators (SW, AG, JT, NB, KM) independently assessed them for inclusion with discrepancies resolved by a sixth reviewer (NF). At the second stage, the same authors screened the individual studies from eligible systematic reviews against inclusion criteria. Hence, the inclusion criteria were applied in full at the individual study level. We also manually searched the cited references of eligible studies to ensure that no relevant systematic reviews were missed. Data extraction and Quality assessment details are available in the eMethods.

### Statistical analysis

All statistical analyses and meta-analyses were performed with R (R Foundation for Statistical Computing, version 4.2.1), using the Umbrella Review Package for R (metaumbrella) [24].

We extracted the most adjusted effect size for each association of individual studies included in each meta-analysis as indicated by the meta-analytic OR, RR, HR or SMD measures, and repeated the meta-analyses to calculate the pooled effect sizes with the 95%CIs using a random-effects model with the restricted maximum likelihood (REML) variance estimator for meta-analyses with 10 or more primary studies and the Hartung, Knapp, Sidik, and Jonkman (HKSJ) method for meta-analyses with fewer than 10 studies [25–27]. The summary effect sizes were subsequently converted into equivalent odds ratios (eORs) for comparative purposes [28]. The direction of the effect sizes was harmonised: an eOR greater than 1 indicated an increased likelihood of the adverse health outcome, while an eOR less than 1 indicated a decreased likelihood of it [29, 30]. When multiple outcomes were assessed using the same primary studies per meta-analysis, we estimated a pooled effect size by assuming a correlation of 0.8 between outcomes [31]. Further details are available in the eMethods.

### Strength of association assessment

Associations with statistically significant (*P* < 0.05) effect sizes were ranked as convincing (Class I), highly suggestive (Class II), suggestive (Class III), or weak (Class IV) evidence according to sample size, strength of association, and assessment of the presence of biases [29]. The criteria for each class are summarised in Supplementary eTable 5 (Supplementary Material 1). Details of the sensitivity analysis are in the eMethods.

## Results

### Literature search

**Fig. 1:**
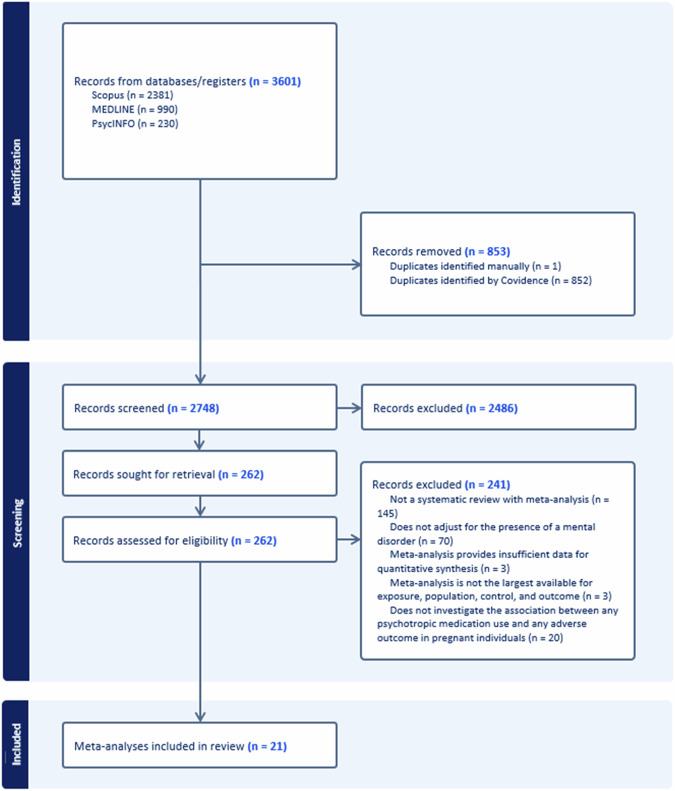
Study selection flow. Flowchart and decision-making process for the inclusion of meta-analyses.

Beginning with 2748 records after duplicate removal, we excluded 2486 records at title and abstract screening, and 241 at full-text, resulting in 21 meta-analyses included [9, 11, 32–50]. The study selection flow is reported in Fig. 1. All studies that were identified by manual search had already been pinpoint in the systematic search. The full list of studies excluded at full-text assessment, with reasons for exclusion, is reported in Supplementary eTable 6 (Supplementary Material 2).

### Study characteristics

The 21 meta-analyses (which encompassed 17,290,755 participants) investigated 66 meta-analytical associations including 242 individual estimates from primary studies (206 cohort studies, 36 case-control studies).The included meta-analyses were published between 2013 and 2022. The quality of the included meta-analyses according to AMSTAR 2 was high in one (5%), low in 12 (57%), and critically low in eight (38%). The median number of individual studies included in each meta-analyses was 3.5 (interquartile range 2–4, range 2–18), the median number of participants was 122,775 (IQR 19,537.5–1,073,324, Range 259–2,673,972), and the median number of cases was 3767 (IQR 682–11895.75, Range 26–90589). The characteristics of the included meta-analyses are presented in Table 1. Further study characteristics are available in the eResults. All variables individual studies adjusted for in their analyses are also available in Supplementary eTable 8.

**Table 1:** Characteristics of included meta-analyses.

Author, year	k	Population (age)	Trimester	Psychotropic medication	Outcome	Quality
Lou, 2022 [32]	8	Mental disorder (adult)	First	SNRI	Congenital malformation, major congenital malformation, cardiac malformation	L
Kautzky, 2022 [33]	33	Depression (adult)	Any	Antidepressant	NICU admission, low 1-minute APGAR, low 5-minute APGAR, respiratory problems	L
Xing, 2020 [34]	54	Depression (adult)	Any	Antidepressant	Preterm birth, low birth weight	L
Chang, 2020 [35]	23	Depression (adult)	Any	SSRI	Preterm birth	CL
Grigoriadis, 2020 [36]	14	Mental disorder (adult)	Any	Benzodiazepine	Preterm birth, low birth weight	L
Andersen, 2020 [37]	29	Opioid use disorder (adult)	Any	Opioid maintenance therapy	Cognition, psychomotor tests	L
Fornaro, 2020 [11]	8	Bipolar disorder (adult)	Any	Lithium	Preterm birth, low birth weight, congenital malformation, cardiac malformation	H
Grigoriadis, 2019 [38]	8	Mental disorder (adult)	Any	Benzodiazepine	Congenital malformation	L
Brown, 2017 [39]	6	Mental disorder (adult)	First, any	SSRI	Autism	L
Berard, 2016 [40]	23	Depression or anxiety (adult)	First	Paroxetine	Cardiac malformation, major congenital malformation	L
Huybrechts, 2014 [41]	41	Mental disorder (adult)	Any	Antidepressant	Preterm birth	L
Ross, 2013 [42]	23	Depression (adult)	Any	Antidepressant	Birth weight, gestational age	L
Gao, 2018 [43]	29	Mental disorder (adult)	Any	SSRI, citalopram, fluoxetine, paroxetine, sertraline	Major congenital malformation, cardiac malformation,	L
Zhao, 2018 [44]	15	Depression (adult)	Any	SSRI	Small for gestational age, low birth weight	L
Poels, 2018 [45]	3	Mental disorder (adult)	Any	Antipsychotic	Neuromotor deficit	CL
Morales, 2018 [46]	18	Affective disorder (adult)	Any	Antidepressant	Autism	CL
Kaplan, 2017 [47]	4	Mental disorder (adult)	Any	SSRI	Autism	CL
Zhou, 2018 [48]	14	Mental disorder (adult)	Any, first, second and third	Antidepressant, SSRI	Autism	CL
Halvorsen, 2019 [49]	18	Mental disorder (adult)	Any, first, second and third	SSRI	Autism, ADHD, mental retardation	CL
Wang, 2023 [73]	5	Depression (adult)	Any	Antidepressant	Gestational diabetes	CL
Grigoriadis, 2022 [50]	7	Mental disorder (adult)	Any, first	Hypnotic benzodiazepine	Congenital malformation, preterm birth	CL

*k* number of studies for each factor, *SSRI* selective serotonin reuptake inhibitor, *SNRI* serotonin and norepinephrine reuptake inhibitors, *NICU* neonatal intensive care unit, *APGAR* appearance, pulse, grimace, activity, and respiration, *ADHD* attention deficit/hyperactivity disorder, *CL* critically low, *L* low, *H* high.

### Summary of associations

Of the 66 meta-analytical associations, 22 (33%) had a nominally statistically significant effect (*P* ≤ 0.05) under random-effects models, however none reached *P* ≤ 10^−6^. Forty-six meta-analytical associations (69%) had greater than 1000 cases. Twenty-one meta-analytical associations (32%) exhibited large heterogeneity (I^2^ > 50%), and 8 (12%) had a 95% prediction interval that excluded the null value. Further, small study effects were found for four (6%) and excess significance bias was found for three meta-analytical associations (5%).

No associations showed convincing (Class I) or highly suggestive (Class II) level of evidence. Five associations (8%) showed suggestive evidence (Class III), 16 (24%) showed weak evidence (Class IV), and 45 (68%) showed no evidence (not significant). A detailed summary of the classification level of evidence is presented in Supplementary eTable 7 (Supplementary Material 3). In the upcoming sections, we primarily describe the associations of weak evidence (Class IV) and higher.

### Strength of evidence for associations between psychotropic drugs and adverse health outcomes

#### Antidepressants

There were 50 associations in this class of psychotropic drug. No associations presented with Class I or II evidence, while 16 (Table 2, Supplementary eTable 7; Supplementary Material 3) presented with suggestive (Class III) or weak (Class IV) evidence.

**Table 2:** Meta-analytical associations between antidepressants and adverse health outcomes in foetus or pregnant individuals supported by convincing, highly suggestive, suggestive, or weak evidence.

Author, year	Trimester	Psychotropic medication	Outcome	Studies		CE	eOR (95%CI)
				(k)	n/No		
Depression							
Xing, 2020	Any	Antidepressant	Preterm birth	10	16630/736843	III	1.65 (1.34, 2.02)
Zhao, 2018	Any	SSRI	Small for gestational age	10	90589/1927094	III	1.50 (1.19, 1.90)
Zhao, 2018	Any	SSRI	Low birth weight	10	41565/1381745	IV	1.38 (1.12, 1.69)
Chang, 2020	Any	SSRI	Preterm birth	4	42/17977	IV	1.46 (1.25, 1.71)
Depression or anxiety							
Berard, 2016	First	Paroxetine	Major congenital malformation	15	88282/2061842	III	1.24 (1.09, 1.40)
Berard, 2016	First	Paroxetine	Cardiac malformation	18	32683/2379469	III	1.28 (1.11, 1.47)
Mental disorder							
Huybrechts, 2014	Any	Antidepressant	Preterm birth	11	12978/236750	III	1.62 (1.24, 2.12)
Halvorsen, 2019	Second and third	SSRI	Autism	2	3135/816568	IV	2.16 (1.93, 2.41)
Halvorsen, 2019	First	SSRI	Autism	4	7093/62954	IV	1.87 (1.04, 3.38)
Zhou, 2018	Second and third	Antidepressant	Autism	4	8909/1501185	IV	1.78 (1.26, 2.52)
Halvorsen, 2019	Second	SSRI	Autism	4	7057/57162	IV	1.73 (1.05, 2.84)
Zhou, 2018	First	SSRI	Autism	3	20259/2262861	IV	1.67 (1.50, 1.85)
Halvorsen, 2019	Any	SSRI	Autism	5	11811/113241	IV	1.60 (1.15, 2.21)
Kaplan, 2017	Any	SSRI	Autism	4	9692/1352844	IV	1.58 (1.15, 2.18)
Zhou, 2018	First	Antidepressant	Autism	5	11924/2015067	IV	1.49 (1.03, 2.17)
Halvorsen, 2019	Any	SSRI	Autism	4	2310/1178689	IV	1.28 (1.13, 1.45)

Results are displayed in descending order of level of evidence and effect size; only associations for which an eOR was available are displayed.

*n* cases, *N* population, *CE* class of evidence (convincing (I), highly suggestive (II), suggestive (III), weak (IV)), *eOR* equivalent odds ratio, *NR* not reported, *k* number of studies for each factor, *SSRI* selective serotonin reuptake inhibitor.

Class III evidence emerged for the following: (1) preterm birth [any mental disorder (eOR 1.62 [95% confidence interval 1.24–2.12]) or depression (1.65 [1.34–2.02]) receiving antidepressants during any trimester of pregnancy]; (2) small for gestational age [pregnant people with depression receiving a SSRI during any trimester of pregnancy (1.50 [1.19–1.90]); and (3) major congenital malformations (1.24 [1.09–1.40]) or cardiac malformations (1.28 [1.11–1.47]) in pregnant people with depression or anxiety receiving paroxetine in first trimester. Sensitivity analyses by study type (restricting to cohort) or by adjusted studies did not change the credibility of any of these associations.

Class IV (weak) evidence emerged for autism in children of pregnant individuals with any mental disorder receiving antidepressants (or specifically SSRIs) during any trimester of pregnancy. Associations without statistically significant effects are in the eResults.

#### Mood stabilisers

Of the eight associations in this class of psychotropic medication, no association presented Class I, II or III evidence. There were five associations (Table 3, Supplementary eTable 7; Supplementary Material 3) that presented with weak evidence (Class IV): cardiac malformations and congenital malformations in pregnant people with bipolar disorder receiving lithium during the first (eOR 1.88 [95% confidence interval 1.26–2.81], 1.97 [1.38–2.79]) or any (1.84 [1.21–2.78], 1.94 [1.19–3.17]) trimester of pregnancy; and preterm birth in pregnant people with bipolar disorder receiving lithium during any trimester of pregnancy (1.91 [1.01–3.63]). Associations without statistically significant effects are in the eResults.

**Table 3:** Meta-analytical associations between mood stabilizers and adverse health outcomes in fetus or pregnant people supported by convincing, highly suggestive, suggestive, or weak evidence.

Author, year	Trimester	Psychotropic medication	Outcome	Studies		CE	eOR (95%CI)
				(k)	n/No		
Bipolar disorder							
Fornaro, 2020	First	Lithium	Congenital malformation	4	984/22225	IV	1.97(1.38, 2.79)
Fornaro, 2020	Any	Lithium	Congenital malformation	3	951/22011	IV	1.94 (1.19, 3.17)
Fornaro, 2020	Any	Lithium	Preterm birth	5	2143/22718	IV	1.91 (1.00, 3.63)
Fornaro, 2020	Any	Lithium	Cardiac malformation	4	15691/1345591	IV	1.84 (1.21, 2.78)
Fornaro, 2020	First	Lithium	Cardiac malformation	4	15691/1345519	IV	1.88 (1.26, 2.81)

Results are displayed in descending order of level of evidence and effect size; only associations for which an eOR was available are displayed.

*n* cases, *N* population, *CE* class of evidence (convincing (I), highly suggestive (II), suggestive (III), weak (IV)), *eOR* equivalent odds ratio, *NR* not reported, *k* number of studies for each factor.

### Antipsychotics

No associations presented Class I, II or III evidence. There was weak evidence (Class IV) for neuromotor deficits in children from pregnant people with any mental disorder receiving antipsychotics during any trimester of pregnancy (Table 4).

**Table 4:** Meta-analytical associations between antipsychotics and adverse health outcomes in foetus or pregnant people supported by convincing, highly suggestive, suggestive, or weak evidence.

Author, year	Trimester	Psychotropic medication	Outcome	Studies		CE	eOR (95%CI)
				(k)	n/No		
Mental disorder							
Poels, 2018	Any	Antipsychotic	Neuromotor deficit	2	26/259	IV	1.63 (1.14, 2.33)

Results are displayed in descending order of level of evidence and effect size; only associations for which an eOR was available are displayed.

*n* cases, *N* population, *CE* class of evidence (convincing (I), highly suggestive (II), suggestive (III), weak (IV)), *eOR* equivalent odds ratio, *k* number of studies for each factor.

### Benzodiazepines

None of the five associations in this class of psychotropic medication had a statistically significant effect (Supplementary eTable 7; Supplementary Material 3), which are listed in the eResults.

### Opioid maintenance therapy

None of the two associations in this class of psychotropic medication had a statistically significant effect (Supplementary eTable 7; Supplementary Material 3), which are listed in the eResults.

## Discussion

In our umbrella review involving 21 meta-analyses of observational studies, we reported that none of the 66 associations between psychotropic medications in pregnancy and safety outcomes had convincing or highly suggestive evidence. A limited number of these associations were supported by suggestive evidence with very small to small effect sizes, namely the association between preterm birth in babies from pregnant people receiving antidepressants with either any mental disorder [41] or depression [34] during any trimester of pregnancy; small for gestational age in pregnant people with depression receiving a SSRI during any trimester of pregnancy [44]; and major congenital malformations or cardiac malformations in newborns of pregnant people with depression or anxiety receiving paroxetine during first trimester of pregnancy [40].

To the authors’ knowledge, this is the first comprehensive umbrella review to systematically assess the risk of adverse health outcomes, both in pregnant people and their children, associated with psychotropic medication use during pregnancy. This umbrella review spans numerous published meta-analyses of observational studies, diligently grading the credibility of evidence with well-established criteria, while carefully controlling for underlying psychiatric conditions.

Understanding that the pregnancy and post-partum are critically vulnerable periods for the mental health of pregnant people is essential. Approximately 15% of pregnant people have a mental disorder and upwards of 13% are on psychotropic medication [51, 52]. Despite this, people on psychotropic medications during pregnancy remain vulnerable to relapse, with even greater risks if medications are discontinued [7, 53]. Often neglected is the risk to the foetus from untreated or inadequately treated maternal psychiatric illness [54, 55]. Even after adjusting for the underlying mental disorder, suggestive evidence indicated an association between antidepressant use during any trimester of pregnancy and preterm birth or small for gestational age, albeit with a very small effect sizes. The exact mechanism for this remains elusive; however, recent research has demonstrated that serotonin reuptake inhibitors may induce sterile foetal membrane inflammation during pregnancy through the p38 MAPK pathway. This could lead to preterm premature rupture of membranes and subsequent preterm birth [56]. Similarly, this inflammatory process could explain the increased risk of small for gestational age, but this area warrants further investigation [57]. Additionally, it is plausible that people with more severe symptoms are more likely to require pharmacological treatment compared to those with milder symptoms, who might benefit from psychosocial or lifestyle interventions. Therefore, the possibility of confounding by severity alongside the diagnosis (i.e., confounding by indication) cannot be overlooked.

We found suggestive evidence associating paroxetine use during the first trimester of pregnancy with the development of major congenital malformation or cardiac malformations. The first trimester is commonly regarded as a highly vulnerable period for teratogenic exposure, as multiple organ systems are being formed by means of organogenesis at this time [58]. The precise mechanism underlying these malformations remains unclear, but it is believed to involve alterations in the serotonin and related receptors that play a crucial role in the development of monoamine-dependent structures [59]. Additionally, there appears to be a dose-dependent response, with doses of paroxetine exceeding 25 mg/day during the first trimester, being associated with a higher risk of both major congenital malformations and major cardiac malformations [60].

Weak evidence was found regarding the potential association between pregnant people with any mental disorder receiving antidepressants during any trimester and the risk of autism in the child. This association did not appear to vary significantly based on the trimester of exposure. It has been hypothesised that a pregnant person’s predisposition to developing a mental disorder, specifically depression or anxiety, may be a primary source of confounding [49]. Evidence has demonstrated that autism shares genetic risk with other psychiatric disorders that require the use of psychotropics [61]. With this in mind, none of the included primary studies in our analysis took maternal family history of mental illness into account. Furthermore, since the majority of persons are in the 20–30 age range when they become parents, and mental disorders can manifest later in life, the increased predisposition for mental illness may not be fully clear prior to pregnancy making this impossible to account for in observational studies.

Lithium use during the first or any trimester in pregnancy was associated with congenital or cardiac malformations, with slightly larger effect size observed within the first trimester, albeit supported by weak evidence [11]. Further, previous research has demonstrated that lithium dosage seems to play a role in determining health outcomes of the foetus whereby the risk of cardiac malformation appears to triple with dosages >900 mg/day compared to those ≤600 mg/day and serum lithium levels >0.64 mEq/L increase the risk of complications [62]. We also found that lithium use during any trimester of pregnancy was associated with preterm birth. This association has not been clearly delineated, however felt to be due to lithium’s propensity to cause either clinical or subclinical hypothyroidism, which has been associated with preterm birth [63, 64]. It is important to note that the abrupt discontinuation of a mood stabiliser such as lithium carries a high risk for morbidity in pregnant people with bipolar disorder [7]. Therefore, in order to minimise adverse health outcomes, clinicians should consider using the lowest effective lithium while balancing the significant risk of relapse [65].

Antipsychotics are often used to treat those with severe mental illness, who are vulnerable for relapse during pregnancy [66]. However, despite antipsychotics being one of the earliest classes of psychotropic medications introduced, there exists only limited research regarding their safety during pregnancy [67]. In our umbrella review, only one meta-analytic association focused on antipsychotic use during any trimester in pregnancy and found that it was weakly associated with neuromotor deficits in the exposed foetus. However, it is important to note that neuromotor deficits in infants may be mild and transient [67]. This finding is rather nonspecific as there were no sub-analyses by first- or second-generation antipsychotics. Although antipsychotics have heterogeneous pharmacodynamic profiles, generally they antagonise the postsynaptic D2 receptors. While there is a paucity of literature in humans, prenatal antipsychotic exposure in rats has demonstrated attenuation of dopamine autoreceptor function and reduction of binding in the mesolimbic pathway [68, 69]. As dopamine is commonly implicated in motor control and development, this prenatal exposure may result in the neuromotor deficits we reported [70].

It is important to note that the commonly used anti-epileptic drugs, such as valproic acid or lamotrigine, were not included in this umbrella review as there existed no meta-analyses examining the use of these medications in mental disorders while accounting for confounding by indication. Here, we must turn to safety data resulting from other conditions such as epilepsy to inform prescribers of medications for which limited data is available [71]. Notably, valproic acid is known to be highly teratogenic and should be avoided in pregnancy [71]. Currently a dynamic meta-analysis database titled metaPreg is being developed which gives direct access with regards to the safety of various drugs during pregnancy, such as the antiepileptic drugs [72]. Although extremely useful, there are currently limited psychotropic medications reported and several of the meta-analytic associations did not control for confounding by indication, nor assess the credibility of evidence which was done in this current study.

### Strengths and limitations

This is the first umbrella review grading the credibility of evidence on the association between psychotropic medications and pregnant people or neonatal outcomes, according to quantitative criteria and accounting for confounding by indication.

Some limitations should be mentioned. First, we did not include or grade evidence from RCTs, instead focusing on observational studies as there is a lack of RCTs in this population due to ethical, methodological considerations and challenges recruiting pregnant people [15, 16]. Observational research can hardly infer causality. Second, due to the lack of randomization, various other confounders may exist for associations, leading to inaccurate conclusions. In this study, we attempted to minimise this by only including studies which accounted for confounding by indication as well as conducting sensitivity analyses for primary studies which adjusted their results based on confounding variables. However, no study accounted for the severity of symptoms, within each diagnostic group. Third, umbrella reviews do not include evidence from individual cohort or case-control studies that have not been previously aggregated into a meta-analysis. Although this may serve as a source of missing information, individual studies are frequently exploratory in nature, require replication, and to be pooled into meta-analyses so that a complete understanding of an association can be appreciated. Fourth, we included meta-analyses based on number of studies rather than overall quality. We opted to include in this way so as to avoid a selection bias which would disregard a large amount of available literature. Fifth, the excess of significant bias was potentially underpowered in the meta-analyses which included only a few studies, however a specific threshold to obtain adequate power has yet to be established. Sixth, the majority of associations focused on adverse health outcomes in the foetus and only one outcome (gestational diabetes) focused on the pregnant person [73]. This demonstrates a paucity of literature in this area, which is imperative to make an informed decision regarding the safety of both foetus and pregnant people. Seventh, the equivalent odds ratio was used to harmonise effect sizes in order to compare strength of associations between various outcomes. Although convenient for comparison, this comes at the expense of losing data on the time-to-event analyses. Eighth, polypharmacy was unable to be fully accounted for in our analysis, with pregnant persons potentially taking more than just a single psychotropic medication. Ninth, the majority of included meta-analyses were of low to critically low quality, largely due to poorly defining the inclusion criteria, not following an a *priori* protocol, and not providing a list of excluded studies with justification. Lastly, for the included studies the cases over the population is not representative of the prevalence for the adverse health outcomes listed. For example, the prevalence of small for gestational age is 27% versus 5% which is reported in this study [74].

To establish a possible causal association between psychotropic medications and adverse health outcomes in pregnant people future studies are required, while controlling for underlying psychiatric conditions and symptom severity, to avoid inaccurate conclusions due to confounding by indication or by severity. This research should examine dose-effect response, severity of psychiatric condition, and mechanisms for adverse health outcomes.

## Conclusions

This umbrella review demonstrated that adverse health outcomes associated with psychotropic medication use in pregnancy are supported by suggestive evidence at best, with no associations being supported by convincing or highly suggestive level of evidence. Safety data from pregnant people with other conditions (i.e., epilepsy) may be used to inform prescription of medication for which there is not data available from populations with mental disorders.

## Supplementary information

Supplementary material 1

Supplementary material 2

Supplementary material 3

Supplementary material 4

Supplementary material 5

## Data Availability

The whole dataset is available from authors upon request.
