# Supplementary material for: Safety of psychotropic medications in pregnancy: an umbrella review
Source: Mol Psychiatry. 2024 Sep 12;30(1):327–35. doi: 10.1038/s41380-024-02697-0 (PMC11649568; doi:10.1038/s41380-024-02697-0)
Supplement: Supplementary file 1 — Supplementary material 1 [file 41380_2024_2697_MOESM1_ESM.docx]

**Supplementary material 1**

**Safety of psychotropic medications in pregnancy: an umbrella review**

Nicholas Fabiano MD^1^, Stanley Wong MD^1,2^, Arnav Gupta MD^3,4^, Jason Tran MD^2^, Nishaant Bhambra MD^5^, Kevin Min BA^6^, Elena Dragioti PhD^7,8^, Corrado Barbui MD^9^, Jess G Fiedorowicz MD PhD^,10,11,12,13^, Corentin J. Gosling PhD^14,15,16^, Samuele Cortese MD PhD^16,17,18,19,20^, Jasmine Gandhi MD^10,12^, Gayatri Saraf MD^10,12,21^, Risa Shorr MLS^22^, Simone N Vigod MD MSc^23^, Benicio N Frey MD PhD^24,25^, Richard Delorme MD PhD^26^, Marco Solmi MD PhD^1,11,12,13,27,#^

1. SCIENCES Lab, Department of Psychiatry, University of Ottawa, Ottawa, ON, Canada
2. Department of Psychiatry, University of Toronto, Toronto, ON, Canada
3. Department of Medicine, University of Calgary, Calgary, AB, Canada
4. College of Public Health, Kent State University, Kent OH, United States
5. Department of Family Medicine, University of Ottawa, Ottawa, ON, Canada
6. Faculty of Medicine, University of Ottawa, Ottawa, ON, Canada
7. Research Laboratory Psychology of Patients, Families & Health Professionals, Department of Nursing, School of Health Sciences, University of Ioannina, Ioannina, Greece
8. Pain and Rehabilitation Centre and Department of Health, Medicine and Caring Sciences, Linköping University, Linköping, Sweden
9. WHO Collaborating Centre for Research and Training in Mental Health and Service Evaluation, Department of Neuroscience, Biomedicine and Movement Sciences, Section of Psychiatry, University of Verona, Verona, Italy
10. Department of Psychiatry, University of Ottawa, Ottawa, ON, Canada
11. Department of Mental Health, The Ottawa Hospital, Ottawa, ON, Canada
12. Ottawa Hospital Research Institute (OHRI) Clinical Epidemiology Program, University of Ottawa, Ottawa, ON, Canada
13. School of Epidemiology and Public Health, Faculty of Medicine, University of Ottawa, Ottawa, ON, Canada
14. DysCo Laboratory, F9200, Université Paris Nanterre, Nanterre, France
15. Laboratory of Psychopathology and Health Process, F92000, Université Paris Cité, Paris, France
16. Centre for Innovation in Mental Health, School of Psychology, Faculty of Environmental and Life Sciences, University of Southampton, Southampton, UK
17. Clinical and Experimental Sciences (CNS and Psychiatry), Faculty of Medicine, University of Southampton, Southampton, UK
18. Solent NHS Trust, Southampton, UK
19. Hassenfeld Children’s Hospital at NYU Langone, New York University Child Study Center, New York City, New York, USA
20. DiMePRe-J-Department of Precision and Regenerative Medicine-Jonic Area, University of Bari “Aldo Moro”, Bari, Italy
21. The Royal's Institute of Mental Health Research, Ottawa, ON, Canada
22. Library Services, The Ottawa Hospital, Ottawa, ON, Canada
23. Department of Psychiatry, Women's College Hospital and University of Toronto, Toronto, Ontario, Canada
24. Department of Psychiatry and Behavioural Neurosciences, McMaster University, Hamilton, Ontario, Canada
25. Women's Health Concerns Clinic, St. Joseph's Healthcare Hamilton, ON, Canada
26. Child and Adolescent Psychiatry Department, Robert Debré Hospital, APHP, University of Paris Cité, Paris, France
27. Department of Child and Adolescent Psychiatry, Charité Universitätsmedizin, Berlin, Germany

**# Corresponding author**

Marco Solmi, MD, PhD

University of Ottawa, Psychiatry Department - 501 Smyth Road, Ottawa, ON, Canada – +1-613-791-5555 - [msolmi@toh.ca](mailto:msolmi@toh.ca)

**Protocol amendments**

The following amendments to the original protocol have been implemented:

We originally planned to use a random-effects model with the restricted maximum likelihood (REML) variance for all meta-analyses. However, after consultation with the BMJ statistical editor for another publication, we opted to use the Hartung, Knapp, Sidik, and Jonkman (HKSJ) method for meta-analyses with less than 10 studies as this method is less prone to type I errors.

Given that credibility of evidence was suggestive at best, we conducted the following sensitivity analyses only for Class III evidence:

- Adjusted studies only
- Cohort studies only

eTable 1. PRIOR checklist.[(1)](https://www.zotero.org/google-docs/?BA4fiT)

| **Section**  **Topic** | **#** | **Item** | **Location Reported** |
| --- | --- | --- | --- |
| **TITLE** | | |  |
| Title | 1 | Identify the report as an overview of reviews. | Page 1 |
| **ABSTRACT** | | |  |
| Abstract | 2 | Provide a comprehensive and accurate summary of the purpose, methods, and results of the overview of reviews. | Page 3 |
| **INTRODUCTION** | | | |
| Rationale | 3 | Describe the rationale for conducting the overview of reviews in the context of existing knowledge. | Page 6 |
| Objectives | 4 | Provide an explicit statement of the objective(s) or question(s) addressed by the overview of reviews. | Page 6 |
| **METHODS** | | |  |
| Eligibility criteria | 5a | Specify the inclusion and exclusion criteria for the overview of reviews. If supplemental primary studies were included, this should be stated, with a rationale. | Page 7 |
|  | 5b | Specify the definition of ‘systematic review’ as used in the inclusion criteria for the overview of reviews. | NA |
| Information sources | 6 | Specify all databases, registers, websites, organizations, reference lists, and other sources searched or consulted to identify systematic reviews and supplemental primary studies (if included).  Specify the date when each source was last searched or consulted. | Page 7 |
| Search strategy | 7 | Present the full search strategies for all databases, registers and websites, such that they could be reproduced. Describe any search filters and limits applied. | Page 7, eTable 3 |
| Selection process | 8a | Describe the methods used to decide whether a systematic review or supplemental primary study (if included) met the inclusion criteria of the overview of reviews. | Page 7 |
|  | 8b | Describe how overlap in the populations, interventions, comparators, and/or outcomes of systematic reviews was identified and managed during study selection. | Page 7 |
| Data collection process | 9a | Describe the methods used to collect data from reports. | Page 7 |
|  | 9b | If applicable, describe the methods used to identify and manage primary study overlap at the level of the comparison and outcome during data collection. For each outcome, specify the method used to illustrate and/or quantify the degree of primary study overlap across systematic reviews. | NA |
|  | 9c | If applicable, specify the methods used to manage discrepant data across systematic reviews during data collection. | NA |
| Data items | 10 | List and define all variables and outcomes for which data were sought. Describe any assumptions made and/or measures taken to identify and clarify missing or unclear information. | Page 7 |
| Risk of bias assessment | 11a | Describe the methods used to assess risk of bias or methodological quality of the included systematic reviews. | Page 8 |
|  | 11b | Describe the methods used to collect data on (from the systematic reviews) and/or assess the risk of bias of the primary studies included in the systematic reviews. Provide a justification for instances where flawed, incomplete, or missing assessments are identified but not re-assessed. | Page 8 |
|  | 11c | Describe the methods used to assess the risk of bias of supplemental primary studies (if included). | NA |
| Synthesis methods | 12a | Describe the methods used to summarize or synthesize results and provide a rationale for the choice(s). | Page 8 |
|  | 12b | Describe any methods used to explore possible causes of heterogeneity among results. | Page 8 |
|  | 12c | Describe any sensitivity analyses conducted to assess the robustness of the synthesized results. | Page 9 |
| Reporting bias assessment | 13 | Describe the methods used to collect data on (from the systematic reviews) and/or assess the risk of bias due to missing results in a summary or synthesis (arising from reporting biases at the levels of the systematic reviews, primary studies, and supplemental primary studies, if included). | Page 8 |
| Certainty of results | 14 | Describe the methods used to collect data on (from the systematic reviews) and/or assess certainty (or confidence) in the body of evidence for an outcome. | Page 8 |
| **RESULTS** | | |  |
| Systematic review  and supplemental  primary study  selection | 15a | Describe the results of the search and selection process, including the number of records screened, assessed for eligibility, and included in the overview of reviews, ideally with a flow diagram. | Page 9 |
|  | 15b | Provide a list of studies that might appear to meet the inclusion criteria, but were excluded, with the main reason for exclusion. | Page 9, eTable 6 |
| Characteristics of  systematic  reviews and  supplemental  primary studies | 16 | Cite each included systematic review and supplemental primary study (if included) and present its characteristics. | Table 1 |
| Primary study  overlap | 17 | Describe the extent of primary study overlap across the included systematic reviews. | NA |
| Risk of bias in  systematic  reviews, primary  studies, and  supplemental  primary studies | 18a | Present assessments of risk of bias or methodological quality for each included systematic review. | Table 1 |
|  | 18b | Present assessments (collected from systematic reviews or assessed anew) of the risk of bias of the primary studies included in the systematic reviews. | eTable 8 |
|  | 18c | Present assessments of the risk of bias of supplemental primary studies (if included). | NA |
| Summary or  synthesis of  results | 19a | For all outcomes, summarize the evidence from the systematic reviews and supplemental primary studies (if included). If meta-analyses were done, present for each the summary estimate and its precision and measures of statistical heterogeneity. If comparing groups, describe the direction of the effect. | Pages 10-11 |
|  | 19b | If meta-analyses were done, present results of all investigations of possible causes of heterogeneity. | Pages 10-11 |
|  | 19c | If meta-analyses were done, present results of all sensitivity analyses conducted to assess the robustness of synthesized results. | Pages 10-11 |
| Reporting biases | 20 | Present assessments (collected from systematic reviews and/or assessed anew) of the risk of bias due to missing primary studies, analyses, or results in a summary or synthesis (arising from reporting biases at the levels of the systematic reviews, primary studies, and supplemental primary studies, if included) for each summary or synthesis assessed. | Pages 10-11 |
| Certainty of  evidence | 21 | Present assessments (collected or assessed anew) of certainty (or confidence) in the body of evidence for each outcome | Pages 10-11 |
| **DISCUSSION** | | |  |
| Discussion | 22a | Summarize the main findings, including any discrepancies in findings across the included systematic reviews and supplemental primary studies (if included). | Pages 11-12 |
|  | 22b | Provide a general interpretation of the results in the context of other evidence. | Pages 11-15 |
|  | 22c | Discuss any limitations of the evidence from systematic reviews, their primary studies, and supplemental primary studies (if included) included in the overview of reviews. Discuss any limitations of the overview of reviews methods used. | Pages 14-15 |
|  | 22d | Discuss implications for practice, policy, and future research (both systematic reviews and primary research). Consider the relevance of the findings to the end users of the overview of reviews, e.g., healthcare providers, policymakers, patients, among others. | Pages 11-15 |
| **OTHER INFORMATION** | | |  |
| Registration and protocol | 23a | Provide registration information for the overview of reviews, including register name and registration number, or state that the overview of reviews was not registered. | Page 6 |
|  | 23b | Indicate where the overview of reviews protocol can be accessed, or state that a protocol was not prepared. | Page 6 |
|  | 23c | Describe and explain any amendments to information provided at registration or in the protocol. Indicate the stage of the overview of reviews at which amendments were made. | Page 6, supplementary material 1 |
| Support | 24 | Describe sources of financial or non-financial support for the overview of reviews, and the role of the funders or sponsors in the overview of reviews. | Page 16 |
| Competing interests | 25 | Declare any competing interests of the overview of reviews' authors. | Pages 15-16 |
| Author information | 26a | Provide contact information for the corresponding author. | Page 1 |
|  | 26b | Describe the contributions of individual authors and identify the guarantor of the overview of reviews. | Page 16 |
| Availability of data and other materials | 27 | Report which of the following are available, where they can be found, and under which conditions they may be accessed: template data collection forms; data collected from included systematic reviews and supplemental primary studies; analytic code; any other materials used in the overview of reviews. | Page 16 |

eTable 2. PRISMA 2020 abstract checklist adapted for umbrella reviews.[(2)](https://www.zotero.org/google-docs/?TNxIkw)

| **Section and Topic** | **Item #** | **Checklist item** | **Reported (Yes/No)** |
| --- | --- | --- | --- |
| **TITLE** | | |  |
| Title | 1 | Identify the report as an umbrella review. | Yes |
| **BACKGROUND** | | |  |
| Objectives | 2 | Provide an explicit statement of the main objective(s) or question(s) the umbrella review addresses. | Yes |
| **METHODS** | | |  |
| Eligibility criteria | 3 | Specify the inclusion and exclusion criteria for the umbrella review. | Yes |
| Information sources | 4 | Specify the information sources (e.g. databases, registers) used to identify studies and the date when each was last searched. | Yes |
| Risk of bias | 5 | Specify the methods used to assess quality in the included reviews/meta-analyses. | Yes |
| Synthesis of results | 6 | Specify the methods used to present and synthesise results, including exploring biases. | Yes |
| **RESULTS** | | |  |
| Included studies | 7 | Give the total number of included comparisons, reviews, studies and describes reviews’ quality. | Yes |
| Synthesis of results | 8 | Present results for main outcomes or of most credible evidence, preferably indicating the number of included studies and participants for each. If  meta-analysis was done, report the summary estimate and confidence/credible interval. If comparing groups, indicate the direction of the effect (i.e.  which group is favoured). | Yes |
| **DISCUSSION** | | |  |
| Limitations of evidence | 9 | Provide a brief summary of the limitations of the evidence included in the umbrella review (e.g. review quality, inconsistency and imprecision). | Yes |
| Interpretation | 10 | Provide a general interpretation of the results and important implications. | Yes |
| **OTHER** | | |  |
| Funding | 11 | Specify the primary source of funding for the umbrella review. | Yes |
| Registration | 12 | Provide the register name and registration number. | Yes |

eTable 3. Search strategy.

| Ovid MEDLINE(R) ALL <1946 to May 04, 2023>    1 exp Psychotropic Drugs/ 392907  2 (antipsychotic* or psychotropic* or neuroleptic*).tw,kf. 79945  3 (clonidine or guanfacine or chlorpromazine or levomepromazine or promazine or acepromazine or triflupromazine or cyamemazine or chlorproethazine or dixyrazine or fluphenazine or perphenazine or prochlorperazine or thiopropazate or trifluoperazine or acetophenazine or thioproperazine or butaperazine or perazine or periciazine or thioridazine or mesoridazine or pipotiazine or haloperidol or trifluperidol or melperone or moperone or pipamperone or bromperidol or benperidol or droperidol or fluanisone or lumateperone or oxypertine or molindone or sertindole or ziprasidone or lurasidone or flupentixol or clopenthixol or chlorprothixene or tiotixene or zuclopenthixol or fluspirilene or pimozide or penfluridol or loxapine or clozapine or olanzapine or quetiapine or asenapine or clotiapine or olanzapine or sulpiride or sultopride or tiapride or remoxipride or amisulpride or veralipride or levosulpiride or lithium or prothipendyl or risperidone or mosapramine or zotepine or aripiprazole or paliperidone or iloperidone or cariprazine or brexpiprazole or pimavanserin or diazepam or chlordiazepoxide or medazepam or oxazepam or potassium clorazepate or lorazepam or adinazolam or bromazepam or clobazam or ketazolam or prazepam or alprazolam or halazepam or pinazepam or camazepam or nordazepam or fludiazepam or ethyl loflazepate or etizolam or clotiazepam or cloxazolam or tofisopam or bentazepam or mexazolam or lorazepam or hydroxyzine or captodiame or hydroxyzine or meprobamate or emylcamate or mebutamate or meprobamate or benzoctamine or buspirone or mephenoxalone or gedocarnil or etifoxine or fabomotizole or Lavandulae aetheroleum or pentobarbital or amobarbital or butobarbital or barbital or aprobarbital or secobarbital or talbutal or vinylbital or vinbarbital or cyclobarbital or heptabarbital or reposal or methohexital or hexobarbital or thiopental or etallobarbital or allobarbital or proxibarbal or barbiturates or chloral hydrate or chloralodol or acetylglycinamide chloral hydrate or dichloralphenazone or paraldehyde or flurazepam or nitrazepam or flunitrazepam or estazolam or triazolam or lormetazepam or temazepam or midazolam or brotizolam or quazepam or loprazolam or doxefazepam or cinolazepam or remimazolam or nimetazepam or glutethimide or methyprylon or pyrithyldione or zopiclone or zolpidem or zaleplon or eszopiclone or melatonin or ramelteon or tasimelteon or methaqualone or clomethiazole or bromisoval or carbromal or scopolamine or propiomazine or triclofos or ethchlorvynol or Valerianae radix or hexapropymate or bromides or apronal or valnoctamide or methylpentynol or niaprazine or dexmedetomidine or suvorexant or Lemborexant or meprobamate or methaqualone or methylpentynol or clomethiazole or emepronium or dipiperonylaminoethanol or desipramine or imipramine or imipramine oxide or clomipramine or opipramol or trimipramine or lofepramine or dibenzepin or amitriptyline or nortriptyline or protriptyline or doxepin or iprindole or melitracen or butriptyline or dosulepin or amoxapine or dimetacrine or amineptine or maprotiline or quinupramine or zimeldine or fluoxetine or citalopram or paroxetine or sertraline or alaproclate or fluvoxamine or etoperidone or escitalopram or isocarboxazid or nialamide or phenelzine or tranylcypromine or iproniazide or iproclozide or moclobemide or toloxatone or oxitriptan or tryptophan or mianserin or nomifensine or trazodone or nefazodone or minaprine or bifemelane or viloxazine or oxaflozane or mirtazapine or bupropion or medifoxamine or tianeptine or pivagabine or venlafaxine or milnacipran or reboxetine or gepirone or duloxetine or agomelatine or desvenlafaxine or vilazodone or Hyperici herba or vortioxetine or esketamine or levomilnacipran or brexanolone or amfetamine or dexamfetamine or metamfetamine or methylphenidate or pemoline or fencamfamine or modafinil or fenozolone or atomoxetine or fenetylline or dexmethylphenidate or lisdexamfetamine or armodafinil or solriamfetol or dexmethylphenidate or caffeine or propentofylline or meclofenoxate or pyritinol or piracetam or deanol or fipexide or citicoline or oxiracetam or pirisudanol or linopirdine or nizofenone or aniracetam or acetylcarnitine or idebenone or prolintane or pipradrol or pramiracetam or adrafinil or vinpocetine or temgicoluril or phenibut or tacrine or donepezil or rivastigmine or galantamine or ipidacrine or donepezil or memantine or Ginkgo folium or aducanumab or nicotine or varenicline or cytisinicline or disulfiram or calcium carbimide or acamprosate or naltrexone or nalmefene or buprenorphine or methadone or levacetylmethadol or lofexidine or levomethadone or diamorphine).tw,kf. 531092  4 exp Selective Serotonin Reuptake Inhibitors/ 45928  5 (Selective Serotonin Reuptake Inhibitor* or ssri).tw,kf. 15815  6 (snri or nri or ndri or tca or nassa or nari or fga or bdz).tw,kf. 21207  7 (antidepressant* or anti depressant*).tw,kf. 76245  8 dopamine antagon*.tw,kf. 2580  9 anti adhd.tw,kf. 23  10 mood stabili?er*.tw,kf. 4410  11 tricyclic*.tw,kf. 17847  12 or/1-11 806860  13 Pregnancy/ 981997  14 exp pregnancy complications/ 472274  15 pregnan*.tw,kf. 606675  16 Pregnant Women/ 14136  17 gestation*.tw,kf. 245152  18 congenital abnormalities/ or abnormalities, drug-induced/ or exp infant, newborn, diseases/ 236683  19 (birth defect* or congenital abnormalit*).tw,kf. 20803  20 ((f?etal or f?etus) adj (malformation* or Anomal* or Defect*)).tw,kf. 5490  21 (infant* or newborn* or neonate*).tw,kf. 674066  22 exp infant/ 1246871  23 or/13-22 2426121  24 12 and 23 38658  25 (systematic review* or meta analy*).pt,tw. 448160  26 ((systematic* adj3 (review* or overview*)) or (methodologic* adj3 (review* or overview*))).tw. 311426  27 ((quantitative adj3 (review* or overview* or synthes*)) or (research adj3 (integrati* or overview*))).tw. 15323  28 25 or 26 or 27 473872  29 24 and 28 997  30 exp animals/ not humans/ 5118677  31 29 not 30 990  Scopus – May 8, 2023  ( TITLE-ABS-KEY ( ( systematic* W/3 ( review* OR overview* ) ) OR ( methodologic* W/3 ( review* OR overview* ) ) ) OR TITLE-ABS-KEY ( ( quantitative W/3 ( review* OR overview* OR synthes* ) ) OR ( research W/3 ( integrati* OR overview* ) ) ) OR TITLE-ABS-KEY ( ( integrative W/3 ( review* OR overview* ) ) OR ( collaborative W/3 ( review* OR overview* ) ) OR ( pool* W/3 analy* ) ) OR TITLE-ABS-KEY ( "data synthes*" OR "data extraction*" OR "data abstraction*" ) OR TITLE-ABS-KEY ( handsearch* OR "hand search*" ) OR TITLE-ABS-KEY ( "mantel haenszel" OR peto OR "der simonian" OR dersimonian OR "fixed effect*" OR "latin square*" ) OR TITLE-ABS-KEY ( "met analy*" OR metanaly* OR "technology assessment*" OR hta OR htas OR "technology overview*" OR "technology appraisal*" ) OR TITLE-ABS-KEY ( "meta regression*" OR metaregression* ) OR TITLE-ABS-KEY ( meta-analy* OR metaanaly* OR "systematic review*" OR "biomedical technology assessment*" OR "bio-medical technology assessment*" ) OR TITLE-ABS-KEY ( medline OR cochrane OR pubmed OR medlars OR embase OR cinahl ) OR SRCTITLE ( cochrane OR ( health W/2 "technology assessment" ) OR "evidence report" ) OR TITLE-ABS-KEY ( comparative W/3 ( efficacy OR effectiveness ) ) OR TITLE-ABS-KEY ( "outcomes research" OR "relative effectiveness" ) OR TITLE-ABS-KEY ( ( indirect OR "indirect treatment" OR mixed-treatment OR bayesian ) W/3 comparison* ) OR TITLE-ABS-KEY ( multi* W/3 treatment W/3 comparison* ) OR TITLE-ABS-KEY ( mixed W/3 treatment W/3 ( meta-analy* OR metaanaly* ) ) OR TITLE-ABS-KEY ( "umbrella review*" ) OR TITLE-ABS-KEY ( multi* W/2 paramet* W/2 evidence W/2 synthesis ) OR TITLE-ABS-KEY ( multiparamet* W/2 evidence W/2 synthesis ) OR TITLE-ABS-KEY ( multi-paramet* W/2 evidence W/2 synthesis ) ) AND ( ( ( TITLE-ABS-KEY ( tricyclic* ) ) OR ( ( TITLE-ABS-KEY ( "mood stabiliser*" ) OR TITLE-ABS-KEY ( "mood stabilizer*" ) ) ) OR ( TITLE-ABS-KEY ( "anti adhd" ) ) OR ( TITLE-ABS-KEY ( ( antidepressant* OR "anti depressant*" ) ) ) OR ( TITLE-ABS-KEY ( ( snri OR nri OR ndri OR tca OR nassa OR nari OR fga OR bdz ) ) ) OR ( TITLE-ABS-KEY ( ( propentofylline OR meclofenoxate OR pyritinol OR piracetam OR deanol OR fipexide OR citicoline OR oxiracetam OR pirisudanol OR linopirdine OR nizofenone OR aniracetam OR acetylcarnitine OR idebenone OR prolintane OR pipradrol OR pramiracetam OR adrafinil OR vinpocetine OR temgicoluril OR phenibut OR tacrine OR donepezil OR rivastigmine OR galantamine OR ipidacrine OR donepezil OR memantine OR "Ginkgo folium" OR aducanumab OR nicotine OR varenicline OR cytisinicline OR disulfiram OR "calcium carbamide" OR acamprosate OR naltrexone OR nalmefene OR buprenorphine OR methadone OR levacetylmethadol OR lofexidine OR levomethadone OR diamorphine ) ) ) OR ( TITLE-ABS-KEY ( ( mirtazapine OR bupropion OR medifoxamine OR tianeptine OR pivagabine OR venlafaxine OR milnacipran OR reboxetine OR gepirone OR duloxetine OR agomelatine OR desvenlafaxine OR vilazodone OR "Hyperici herba" OR vortioxetine OR esketamine OR levomilnacipran OR brexanolone OR amfetamine OR dexamfetamine OR metamfetamine OR methylphenidate OR pemoline OR fencamfamine OR modafinil OR fenozolone OR atomoxetine OR fenetylline OR dexmethylphenidate OR lisdexamfetamine OR armodafinil OR solriamfetol OR dexmethylphenidate OR caffeine ) ) ) OR ( TITLE-ABS-KEY ( ( amoxapine OR dimetacrine OR amineptine OR maprotiline OR quinupramine OR zimeldine OR fluoxetine OR citalopram OR paroxetine OR sertraline OR alaproclate OR fluvoxamine OR etoperidone OR escitalopram OR isocarboxazid OR nialamide OR phenelzine OR tranylcypromine OR iproniazide OR iproclozide OR moclobemide OR toloxatone OR oxitriptan OR tryptophan OR mianserin OR nomifensine OR trazodone OR nefazodone OR minaprine OR bifemelane OR viloxazine OR oxaflozane ) ) ) OR ( TITLE-ABS-KEY ( ( valnoctamide OR methylpentynol OR niaprazine OR dexmedetomidine OR suvorexant OR lemborexant OR meprobamate OR methaqualone OR methylpentynol OR clomethiazole OR emepronium OR dipiperonylaminoethanol OR desipramine OR imipramine OR "imipramine oxide" OR clomipramine OR opipramol OR trimipramine OR lofepramine OR dibenzepin OR amitriptyline OR nortriptyline OR protriptyline OR doxepin OR iprindole OR melitracen OR butriptyline OR dosulepin ) ) ) OR ( TITLE-ABS-KEY ( ( brotizolam OR quazepam OR loprazolam OR doxefazepam OR cinolazepam OR remimazolam OR nimetazepam OR glutethimide OR methyprylon OR pyrithyldione OR zopiclone OR zolpidem OR zaleplon OR eszopiclone OR melatonin OR ramelteon OR tasimelteon OR methaqualone OR clomethiazole OR bromisoval OR carbromal OR scopolamine OR propiomazine OR triclofos OR ethchlorvynol OR "Valerianae radix" OR hexapropymate OR bromides OR apronal ) ) ) OR ( TITLE-ABS-KEY ( ( barbital OR aprobarbital OR secobarbital OR talbutal OR vinylbital OR vinbarbital OR cyclobarbital OR heptabarbital OR reposal OR methohexital OR hexobarbital OR thiopental OR etallobarbital OR allobarbital OR proxibarbal OR barbiturates OR "chloral hydrate" OR chloralodol OR "acetylglycinamide chloral hydrate" OR dichloralphenazone OR paraldehyde OR flurazepam OR nitrazepam OR flunitrazepam OR estazolam OR triazolam OR lormetazepam OR temazepam OR midazolam ) ) ) OR ( TITLE-ABS-KEY ( ( cloxazolam OR tofisopam OR bentazepam OR mexazolam OR lorazepam OR hydroxyzine OR captodiame OR hydroxyzine OR meprobamate OR emylcamate OR mebutamate OR meprobamate OR benzoctamine OR buspirone OR mephenoxalone OR gedocarnil OR etifoxine OR fabomotizole OR "Lavandulae aetheroleum" OR pentobarbital OR amobarbital OR butobarbital ) ) ) OR ( TITLE-ABS-KEY ( ( risperidone OR mosapramine OR zotepine OR aripiprazole OR paliperidone OR iloperidone OR cariprazine OR brexpiprazole OR pimavanserin OR diazepam OR chlordiazepoxide OR medazepam OR oxazepam OR "potassium clorazepate" OR lorazepam OR adinazolam OR bromazepam OR clobazam OR ketazolam OR prazepam OR alprazolam OR halazepam OR pinazepam OR camazepam OR nordazepam OR fludiazepam OR "ethyl loflazepate" OR etizolam OR clotiazepam ) ) ) OR ( TITLE-ABS-KEY ( ( benperidol OR droperidol OR fluanisone OR lumateperone OR oxypertine OR molindone OR sertindole OR ziprasidone OR lurasidone OR flupentixol OR clopenthixol OR chlorprothixene OR tiotixene OR zuclopenthixol OR fluspirilene OR pimozide OR penfluridol OR loxapine OR clozapine OR olanzapine OR quetiapine OR asenapine OR clotiapine OR olanzapine OR sulpiride OR sultopride OR tiapride OR remoxipride OR amisulpride OR veralipride OR levosulpiride OR lithium OR prothipendyl ) ) ) OR ( TITLE-ABS-KEY ( ( clonidine OR guanfacine OR chlorpromazine OR levomepromazine OR promazine OR acepromazine OR triflupromazine OR cyamemazine OR chlorproethazine OR dixyrazine OR fluphenazine OR perphenazine OR prochlorperazine OR thiopropazate OR trifluoperazine OR acetophenazine OR thioproperazine OR butaperazine OR perazine OR periciazine OR thioridazine OR mesoridazine OR pipotiazine OR haloperidol OR trifluperidol OR melperone OR moperone OR pipamperone OR bromperidol ) ) ) OR ( TITLE-ABS-KEY ( ( antipsychotic* OR psychotropic* OR neuroleptic* ) ) ) ) AND ( ( TITLE-ABS ( pregnan* ) ) OR ( TITLE-ABS ( gestation* ) ) OR ( TITLE-ABS ( ( infant* OR newborn* OR neonate* OR preterm* ) ) ) OR ( ( TITLE-ABS-KEY ( "birth defect*" ) OR TITLE-ABS-KEY ( "congenital abnormalit*" ) ) ) OR ( TITLE-ABS ( fetal OR foetal OR fetus OR foetus ) AND TITLE-ABS ( malformation* OR anomal* OR defect* ) ) ) )  2381  APA PsycInfo <1806 to May Week 1 2023>    1 exp psychotropic drugs/ 93880  2 (antipsychotic* or psychotropic* or neuroleptic*).tw. 54241  3 (clonidine or guanfacine or chlorpromazine or levomepromazine or promazine or acepromazine or triflupromazine or cyamemazine or chlorproethazine or dixyrazine or fluphenazine or perphenazine or prochlorperazine or thiopropazate or trifluoperazine or acetophenazine or thioproperazine or butaperazine or perazine or periciazine or thioridazine or mesoridazine or pipotiazine or haloperidol or trifluperidol or melperone or moperone or pipamperone or bromperidol or benperidol or droperidol or fluanisone or lumateperone or oxypertine or molindone or sertindole or ziprasidone or lurasidone or flupentixol or clopenthixol or chlorprothixene or tiotixene or zuclopenthixol or fluspirilene or pimozide or penfluridol or loxapine or clozapine or olanzapine or quetiapine or asenapine or clotiapine or olanzapine or sulpiride or sultopride or tiapride or remoxipride or amisulpride or veralipride or levosulpiride or lithium or prothipendyl or risperidone or mosapramine or zotepine or aripiprazole or paliperidone or iloperidone or cariprazine or brexpiprazole or pimavanserin or diazepam or chlordiazepoxide or medazepam or oxazepam or potassium clorazepate or lorazepam or adinazolam or bromazepam or clobazam or ketazolam or prazepam or alprazolam or halazepam or pinazepam or camazepam or nordazepam or fludiazepam or ethyl loflazepate or etizolam or clotiazepam or cloxazolam or tofisopam or bentazepam or mexazolam or lorazepam or hydroxyzine or captodiame or hydroxyzine or meprobamate or emylcamate or mebutamate or meprobamate or benzoctamine or buspirone or mephenoxalone or gedocarnil or etifoxine or fabomotizole or Lavandulae aetheroleum or pentobarbital or amobarbital or butobarbital or barbital or aprobarbital or secobarbital or talbutal or vinylbital or vinbarbital or cyclobarbital or heptabarbital or reposal or methohexital or hexobarbital or thiopental or etallobarbital or allobarbital or proxibarbal or barbiturates or chloral hydrate or chloralodol or acetylglycinamide chloral hydrate or dichloralphenazone or paraldehyde or flurazepam or nitrazepam or flunitrazepam or estazolam or triazolam or lormetazepam or temazepam or midazolam or brotizolam or quazepam or loprazolam or doxefazepam or cinolazepam or remimazolam or nimetazepam or glutethimide or methyprylon or pyrithyldione or zopiclone or zolpidem or zaleplon or eszopiclone or melatonin or ramelteon or tasimelteon or methaqualone or clomethiazole or bromisoval or carbromal or scopolamine or propiomazine or triclofos or ethchlorvynol or Valerianae radix or hexapropymate or bromides or apronal or valnoctamide or methylpentynol or niaprazine or dexmedetomidine or suvorexant or Lemborexant or meprobamate or methaqualone or methylpentynol or clomethiazole or emepronium or dipiperonylaminoethanol or desipramine or imipramine or imipramine oxide or clomipramine or opipramol or trimipramine or lofepramine or dibenzepin or amitriptyline or nortriptyline or protriptyline or doxepin or iprindole or melitracen or butriptyline or dosulepin or amoxapine or dimetacrine or amineptine or maprotiline or quinupramine or zimeldine or fluoxetine or citalopram or paroxetine or sertraline or alaproclate or fluvoxamine or etoperidone or escitalopram or isocarboxazid or nialamide or phenelzine or tranylcypromine or iproniazide or iproclozide or moclobemide or toloxatone or oxitriptan or tryptophan or mianserin or nomifensine or trazodone or nefazodone or minaprine or bifemelane or viloxazine or oxaflozane or mirtazapine or bupropion or medifoxamine or tianeptine or pivagabine or venlafaxine or milnacipran or reboxetine or gepirone or duloxetine or agomelatine or desvenlafaxine or vilazodone or Hyperici herba or vortioxetine or esketamine or levomilnacipran or brexanolone or amfetamine or dexamfetamine or metamfetamine or methylphenidate or pemoline or fencamfamine or modafinil or fenozolone or atomoxetine or fenetylline or dexmethylphenidate or lisdexamfetamine or armodafinil or solriamfetol or dexmethylphenidate or caffeine or propentofylline or meclofenoxate or pyritinol or piracetam or deanol or fipexide or citicoline or oxiracetam or pirisudanol or linopirdine or nizofenone or aniracetam or acetylcarnitine or idebenone or prolintane or pipradrol or pramiracetam or adrafinil or vinpocetine or temgicoluril or phenibut or tacrine or donepezil or rivastigmine or galantamine or ipidacrine or donepezil or memantine or Ginkgo folium or aducanumab or nicotine or varenicline or cytisinicline or disulfiram or calcium carbimide or acamprosate or naltrexone or nalmefene or buprenorphine or methadone or levacetylmethadol or lofexidine or levomethadone or diamorphine).tw. 146849  4 (Selective Serotonin Reuptake Inhibitor* or ssri).tw. 9574  5 (antidepressant* or anti depressant*).tw. 43986  6 dopamine antagon*.tw. 1145  7 anti adhd.tw. 23  8 mood stabili?er*.tw. 3892  9 tricyclic*.tw. 5820  10 or/1-9 212584  11 pregnancy/ or exp obstetrical complications/ 27798  12 exp pregnancy outcomes/ 23805  13 pregnan*.tw. 55609  14 gestation*.tw. 15307  15 exp congenital disorders/ or teratogens/ or teratology/ 9233  16 (birth defect* or congenital abnormalit*).tw. 1022  17 ((f?etal or f?etus) adj (malformation* or Anomal* or Defect*)).tw. 248  18 (infant* or newborn* or neonate*).tw. 101718  19 premature birth/ 6480  20 or/11-19 167915  21 10 and 20 5650  22 meta analysis/ or "systematic review"/ 6002  23 ((systematic* adj3 (review* or overview*)) or (methodologic* adj3 (review* or overview*))).tw. 54283  24 (quantitative adj3 (review* or overview* or synthes*)).tw. 2877  25 (research adj3 (integrati* or overview*)).tw. 8736  26 (data synthes* or data extraction* or data abstraction*).tw. 3774  27 (met analy* or metanaly* or technology assessment* or HTA or HTAs or technology overview* or technology appraisal*).tw. 1128  28 (meta regression* or metaregression*).tw. 2601  29 ("systematic review" or meta anal*).md. 62918  30 or/22-29 92128  31 21 and 30 230 |
| --- |

eTable 4. List of psychotropic medications in the Anatomical Therapeutic Chemical (ATC) World Health Organization (WHO) database.

| N05AA01 chlorpromazine  N05AA02 levomepromazine  N05AA03 promazine  N05AA04 acepromazine  N05AA05 triflupromazine  N05AA06 cyamemazine  N05AA07 chlorproethazine  N05AB01 dixyrazine  N05AB02 fluphenazine  N05AB03 perphenazine  N05AB04 prochlorperazine  N05AB05 thiopropazate  N05AB06 trifluoperazine  N05AB07 acetophenazine  N05AB08 thioproperazine  N05AB09 butaperazine  N05AB10 perazine  N05AC01 periciazine  N05AC02 thioridazine  N05AC03 mesoridazine  N05AC04 pipotiazine  N05AD01 haloperidol  N05AD02 trifluperidol  N05AD03 melperone  N05AD04 moperone  N05AD05 pipamperone  N05AD06 bromperidol  N05AD07 benperidol  N05AD08 droperidol  N05AD09 fluanisone  N05AD10 lumateperone  N05AE01 oxypertine  N05AE02 molindone  N05AE03 sertindole  N05AE04 ziprasidone  N05AE05 lurasidone  N05AF01 flupentixol  N05AF02 clopenthixol  N05AF03 chlorprothixene  N05AF04 tiotixene  N05AF05 zuclopenthixol  N05AG01 fluspirilene  N05AG02 pimozide  N05AG03 penfluridol  N05AH01 loxapine  N05AH02 clozapine  N05AH03 olanzapine  N05AH04 quetiapine  N05AH05 asenapine  N05AH06 clotiapine  N05AH53 olanzapine and samidorphan  N05AL01 sulpiride  N05AL02 sultopride  N05AL03 tiapride  N05AL04 remoxipride  N05AL05 amisulpride  N05AL06 veralipride  N05AL07 levosulpiride  N05AN01 lithium  N05AX07 prothipendyl  N05AX08 risperidone  N05AX10 mosapramine  N05AX11 zotepine  N05AX12 aripiprazole  N05AX13 paliperidone  N05AX14 iloperidone  N05AX15 cariprazine  N05AX16 brexpiprazole  N05AX17 pimavanserin  N05BA01 diazepam  N05BA02 chlordiazepoxide  N05BA03 medazepam  N05BA04 oxazepam  N05BA05 potassium clorazepate  N05BA06 lorazepam  N05BA07 adinazolam  N05BA08 bromazepam  N05BA09 clobazam  N05BA10 ketazolam  N05BA11 prazepam  N05BA12 alprazolam  N05BA13 halazepam  N05BA14 pinazepam  N05BA15 camazepam  N05BA16 nordazepam  N05BA17 fludiazepam  N05BA18 ethyl loflazepate  N05BA19 etizolam  N05BA21 clotiazepam  N05BA22 cloxazolam  N05BA23 tofisopam  N05BA24 bentazepam  N05BA25 mexazolam  N05BA56 lorazepam, combinations  N05BB01 hydroxyzine  N05BB02 captodiame  N05BB51 hydroxyzine, combinations  N05BC01 meprobamate  N05BC03 emylcamate  N05BC04 mebutamate  N05BC51 meprobamate, combinations  N05BD01 benzoctamine  N05BE01 buspirone  N05BX01 mephenoxalone  N05BX02 gedocarnil  N05BX03 etifoxine  N05BX04 fabomotizole  N05BX05 Lavandulae aetheroleum  N05CA01 pentobarbital  N05CA02 amobarbital  N05CA03 butobarbital  N05CA04 barbital  N05CA05 aprobarbital  N05CA06 secobarbital  N05CA07 talbutal  N05CA08 vinylbital  N05CA09 vinbarbital  N05CA10 cyclobarbital  N05CA11 heptabarbital  N05CA12 reposal  N05CA15 methohexital  N05CA16 hexobarbital  N05CA19 thiopental  N05CA20 etallobarbital  N05CA21 allobarbital  N05CA22 proxibarbal  N05CB01 combinations of barbiturates  N05CB02 barbiturates in combination with other drugs  N05CC01 chloral hydrate  N05CC02 chloralodol  N05CC03 acetylglycinamide chloral hydrate  N05CC04 dichloralphenazone  N05CC05 paraldehyde  N05CD01 flurazepam  N05CD02 nitrazepam  N05CD03 flunitrazepam  N05CD04 estazolam  N05CD05 triazolam  N05CD06 lormetazepam  N05CD07 temazepam  N05CD08 midazolam  N05CD09 brotizolam  N05CD10 quazepam  N05CD11 loprazolam  N05CD12 doxefazepam  N05CD13 cinolazepam  N05CD14 remimazolam  N05CD15 nimetazepam  N05CE01 glutethimide  N05CE02 methyprylon  N05CE03 pyrithyldione  N05CF01 zopiclone  N05CF02 zolpidem  N05CF03 zaleplon  N05CF04 eszopiclone  N05CH01 melatonin  N05CH02 ramelteon  N05CH03 tasimelteon  N05CM01 methaqualone  N05CM02 clomethiazole  N05CM03 bromisoval  N05CM04 carbromal  N05CM05 scopolamine  N05CM06 propiomazine  N05CM07 triclofos  N05CM08 ethchlorvynol  N05CM09 Valerianae radix  N05CM10 hexapropymate  N05CM11 bromides  N05CM12 apronal  N05CM13 valnoctamide  N05CM15 methylpentynol  N05CM16 niaprazine  N05CM18 dexmedetomidine  N05CM19 suvorexant  N05CM21 lemborexant  N05CX01 meprobamate, combinations  N05CX02 methaqualone, combinations  N05CX03 methylpentynol, combinations  N05CX04 clomethiazole, combinations  N05CX05 emepronium, combinations  N05CX06 dipiperonylaminoethanol, combinations  N06AA01 desipramine  N06AA02 imipramine  N06AA03 imipramine oxide  N06AA04 clomipramine  N06AA05 opipramol  N06AA06 trimipramine  N06AA07 lofepramine  N06AA08 dibenzepin  N06AA09 amitriptyline  N06AA10 nortriptyline  N06AA11 protriptyline  N06AA12 doxepin  N06AA13 iprindole  N06AA14 melitracen  N06AA15 butriptyline  N06AA16 dosulepin  N06AA17 amoxapine  N06AA18 dimetacrine  N06AA19 amineptine  N06AA21 maprotiline  N06AA23 quinupramine  N06AB02 zimeldine  N06AB03 fluoxetine  N06AB04 citalopram  N06AB05 paroxetine  N06AB06 sertraline  N06AB07 alaproclate  N06AB08 fluvoxamine  N06AB09 etoperidone  N06AB10 escitalopram  N06AF01 isocarboxazid  N06AF02 nialamide  N06AF03 phenelzine  N06AF04 tranylcypromine  N06AF05 iproniazide  N06AF06 iproclozide  N06AG02 moclobemide  N06AG03 toloxatone  N06AX01 oxitriptan  N06AX02 tryptophan  N06AX03 mianserin  N06AX04 nomifensine  N06AX05 trazodone  N06AX06 nefazodone  N06AX07 minaprine  N06AX08 bifemelane  N06AX09 viloxazine  N06AX10 oxaflozane  N06AX11 mirtazapine  N06AX12 bupropion  N06AX13 medifoxamine  N06AX14 tianeptine  N06AX15 pivagabine  N06AX16 venlafaxine  N06AX17 milnacipran  N06AX18 reboxetine  N06AX19 gepirone  N06AX21 duloxetine  N06AX22 agomelatine  N06AX23 desvenlafaxine  N06AX24 vilazodone  N06AX25 Hyperici herba  N06AX26 vortioxetine  N06AX27 esketamine  N06AX28 levomilnacipran  N06AX29 brexanolone  N06BA01 amfetamine  N06BA02 dexamfetamine  N06BA03 metamfetamine  N06BA04 methylphenidate  N06BA05 pemoline  N06BA06 fencamfamin  N06BA07 modafinil  N06BA08 fenozolone  N06BA09 atomoxetine  N06BA10 fenetylline  N06BA11 dexmethylphenidate  N06BA12 lisdexamfetamine  N06BA13 armodafinil  N06BA14 solriamfetol  N06BA15 dexmethylphenidate and serdexmethylphenidate  N06BC01 caffeine  N06BC02 propentofylline  N06BX01 meclofenoxate  N06BX02 pyritinol  N06BX03 piracetam  N06BX04 deanol  N06BX05 fipexide  N06BX06 citicoline  N06BX07 oxiracetam  N06BX08 pirisudanol  N06BX09 linopirdine  N06BX10 nizofenone  N06BX11 aniracetam  N06BX12 acetylcarnitine  N06BX13 idebenone  N06BX14 prolintane  N06BX15 pipradrol  N06BX16 pramiracetam  N06BX17 adrafinil  N06BX18 vinpocetine  N06BX21 temgicoluril  N06BX22 phenibut  N06CA01 amitriptyline and psycholeptics  N06CA02 melitracen and psycholeptics  N06CA03 fluoxetine and psycholeptics  N06DA01 tacrine  N06DA02 donepezil  N06DA03 rivastigmine  N06DA04 galantamine  N06DA05 ipidacrine  N06DA52 donepezil and memantine  N06DA53 donepezil, memantine and Ginkgo folium  N06DX01 memantine  N06DX02 Ginkgo folium  N06DX03 aducanumab  N06DX30 combinations  N07BA01 nicotine  N07BA03 varenicline  N07BA04 cytisinicline  N07BB01 disulfiram  N07BB02 calcium carbimide  N07BB03 acamprosate  N07BB04 naltrexone  N07BB05 nalmefene  N07BC01 buprenorphine  N07BC02 methadone  N07BC03 levacetylmethadol  N07BC04 lofexidine  N07BC05 levomethadone  N07BC06 diamorphine  N07BC51 buprenorphine, combinations  C02AC02 guanfacine  C02AC01 clonidine |
| --- |

eTable 5. Classes of credibility of evidence.

| Class | Requirements |
| --- | --- |
| I | >1000 cases, p-value<10^-6^ based on random effects meta-analysis, no evidence of small-study effect or excess significance bias, has a 95% prediction interval (PI) that excludes the null value, the largest study has a nominally significant effect size (P<0.05), and has low or moderate between-study heterogeneity (I^2^<50%) |
| II | >1000 cases, p-value<10^-6^ based on random effects meta-analysis, and the largest study has a nominally significant effect size (P<0.05) |
| III | >1000 cases, and a p-value<10^-3^ based on random effects meta-analysis |
| IV | All other remained associations with a p-value ≤0.05 |

**References**

[1. Gates M, Gates A, Pieper D, Fernandes RM, Tricco AC, Moher D, et al. Reporting guideline for overviews of reviews of healthcare interventions: development of the PRIOR statement. BMJ. 2022 Aug 9;378:e070849.](https://www.zotero.org/google-docs/?cnN6LN)

[2. Page MJ, McKenzie JE, Bossuyt PM, Boutron I, Hoffmann TC, Mulrow CD, et al. The PRISMA 2020 statement: an updated guideline for reporting systematic reviews. BMJ. 2021 Mar 29;372:n71.](https://www.zotero.org/google-docs/?cnN6LN)
