# Supplementary material for: Safety of psychotropic medications in pregnancy: an umbrella review
Source: Mol Psychiatry. 2024 Sep 12;30(1):327–35. doi: 10.1038/s41380-024-02697-0 (PMC11649568; doi:10.1038/s41380-024-02697-0)
Supplement: Supplementary file 2 — Supplementary material 2 [file 41380_2024_2697_MOESM2_ESM.docx]

**Supplementary material 2**

**Safety of psychotropic medications in pregnancy: an umbrella review**

Nicholas Fabiano MD^1^, Stanley Wong MD^1,2^, Arnav Gupta MD^3,4^, Jason Tran MD^2^, Nishaant Bhambra MD^5^, Kevin Min BA^6^, Elena Dragioti PhD^7,8^, Corrado Barbui MD^9^, Jess G Fiedorowicz MD PhD^,10,11,12,13^, Corentin J. Gosling PhD^14,15,16^, Samuele Cortese MD PhD^16,17,18,19,20^, Jasmine Gandhi MD^10,12^, Gayatri Saraf MD^10,12,21^, Risa Shorr MLS^22^, Simone N Vigod MD MSc^23^, Benicio N Frey MD PhD^24,25^, Richard Delorme MD PhD^26^, Marco Solmi MD PhD^1,11,12,13,27,#^

1. SCIENCES Lab, Department of Psychiatry, University of Ottawa, Ottawa, ON, Canada
2. Department of Psychiatry, University of Toronto, Toronto, ON, Canada
3. Department of Medicine, University of Calgary, Calgary, AB, Canada
4. College of Public Health, Kent State University, Kent OH, United States
5. Department of Family Medicine, University of Ottawa, Ottawa, ON, Canada
6. Faculty of Medicine, University of Ottawa, Ottawa, ON, Canada
7. Research Laboratory Psychology of Patients, Families & Health Professionals, Department of Nursing, School of Health Sciences, University of Ioannina, Ioannina, Greece
8. Pain and Rehabilitation Centre and Department of Health, Medicine and Caring Sciences, Linköping University, Linköping, Sweden
9. WHO Collaborating Centre for Research and Training in Mental Health and Service Evaluation, Department of Neuroscience, Biomedicine and Movement Sciences, Section of Psychiatry, University of Verona, Verona, Italy
10. Department of Psychiatry, University of Ottawa, Ottawa, ON, Canada
11. Department of Mental Health, The Ottawa Hospital, Ottawa, ON, Canada
12. Ottawa Hospital Research Institute (OHRI) Clinical Epidemiology Program, University of Ottawa, Ottawa, ON, Canada
13. School of Epidemiology and Public Health, Faculty of Medicine, University of Ottawa, Ottawa, ON, Canada
14. DysCo Laboratory, F9200, Université Paris Nanterre, Nanterre, France
15. Laboratory of Psychopathology and Health Process, F92000, Université Paris Cité, Paris, France
16. Centre for Innovation in Mental Health, School of Psychology, Faculty of Environmental and Life Sciences, University of Southampton, Southampton, UK
17. Clinical and Experimental Sciences (CNS and Psychiatry), Faculty of Medicine, University of Southampton, Southampton, UK
18. Solent NHS Trust, Southampton, UK
19. Hassenfeld Children’s Hospital at NYU Langone, New York University Child Study Center, New York City, New York, USA
20. DiMePRe-J-Department of Precision and Regenerative Medicine-Jonic Area, University of Bari “Aldo Moro”, Bari, Italy
21. The Royal's Institute of Mental Health Research, Ottawa, ON, Canada
22. Library Services, The Ottawa Hospital, Ottawa, ON, Canada
23. Department of Psychiatry, Women's College Hospital and University of Toronto, Toronto, Ontario, Canada
24. Department of Psychiatry and Behavioural Neurosciences, McMaster University, Hamilton, Ontario, Canada
25. Women's Health Concerns Clinic, St. Joseph's Healthcare Hamilton, ON, Canada
26. Child and Adolescent Psychiatry Department, Robert Debré Hospital, APHP, University of Paris Cité, Paris, France
27. Department of Child and Adolescent Psychiatry, Charité Universitätsmedizin, Berlin, Germany

**# Corresponding author**

Marco Solmi, MD, PhD

University of Ottawa, Psychiatry Department - 501 Smyth Road, Ottawa, ON, Canada – +1-613-791-5555 - [msolmi@toh.ca](mailto:msolmi@toh.ca)

eTable 6. List and reason of excluded studies.

| **Study** | **Reason for Exclusion** |
| --- | --- |
| 't Jong 2012[(1)](https://www.zotero.org/google-docs/?ns12Qp) | Not a systematic review with meta-analysis |
| Addis, 2000[(2)](https://www.zotero.org/google-docs/?5AkJEc) | Does not adjust for the presence of a mental disorder |
| Addis, 2000[(2)](https://www.zotero.org/google-docs/?luB7jg) | Does not adjust for the presence of a mental disorder |
| Andalib, 2017[(3)](https://www.zotero.org/google-docs/?EsfTYp) | Does not adjust for the presence of a mental disorder |
| Andrade, 2017[(4)](https://www.zotero.org/google-docs/?b23oM5) | Not a systematic review with meta-analysis |
| Andrade, 2018[(5)](https://www.zotero.org/google-docs/?Lm1ItP) | Not a systematic review with meta-analysis |
| Andrade, 2019[(6)](https://www.zotero.org/google-docs/?JNjKJm) | Not a systematic review with meta-analysis |
| Andrade, 2019[(7)](https://www.zotero.org/google-docs/?KVd0sA) | Not a systematic review with meta-analysis |
| Andrade, 2021[(8)](https://www.zotero.org/google-docs/?MT0CVK) | Not a systematic review with meta-analysis |
| Andrade, 2022[(9)](https://www.zotero.org/google-docs/?aDIy24) | Not a systematic review with meta-analysis |
| Anonymous, 2020[(10)](https://www.zotero.org/google-docs/?SDnJUF) | Not a systematic review with meta-analysis |
| Anonymous, 2021[(11)](https://www.zotero.org/google-docs/?ujJVWE) | Not a systematic review with meta-analysis |
| Araujo, 2020[(12)](https://www.zotero.org/google-docs/?RkXIsO) | Not a systematic review with meta-analysis |
| Athar, 2022[(13)](https://www.zotero.org/google-docs/?qZOcSm) | Does not adjust for the presence of a mental disorder |
| Austin, 1998[(14)](https://www.zotero.org/google-docs/?W8UUbE) | Not a systematic review with meta-analysis |
| Balalian, 2023[(15)](https://www.zotero.org/google-docs/?tJC7Sc) | Not a systematic review with meta-analysis |
| Baldacchino, 2014[(16)](https://www.zotero.org/google-docs/?pDQT2R) | Does not adjust for the presence of a mental disorder |
| Banti, 2009[(17)](https://www.zotero.org/google-docs/?GVG7cc) | Not a systematic review with meta-analysis |
| Bar-Oz, 2007[(18)](https://www.zotero.org/google-docs/?gYFGRE) | Does not adjust for the presence of a mental disorder |
| Beex-Oosterhuis, 2021[(19)](https://www.zotero.org/google-docs/?2ZMCJY) | Not a systematic review with meta-analysis |
| Beex-Oosterhuis, 2022[(19)](https://www.zotero.org/google-docs/?dIrbqt) | Not a systematic review with meta-analysis |
| Bellantuono, 2007[(20)](https://www.zotero.org/google-docs/?UxMEQE) | Not a systematic review with meta-analysis |
| Bellantuono, 2012[(21)](https://www.zotero.org/google-docs/?COOYlK) | Not a systematic review with meta-analysis |
| Bellantuono, 2015[(22)](https://www.zotero.org/google-docs/?b7nk4j) | Not a systematic review with meta-analysis |
| Berwaerts, 2009[(23)](https://www.zotero.org/google-docs/?Ha5Gvz) | Not a systematic review with meta-analysis |
| Besag, 2023[(24)](https://www.zotero.org/google-docs/?rtFJsH) | Not a systematic review with meta-analysis |
| Blanc, 2021[(25)](https://www.zotero.org/google-docs/?2Km9gV) | Not a systematic review with meta-analysis |
| Bolea-Alamanac, 2014[(26)](https://www.zotero.org/google-docs/?ME9gNl) | Not a systematic review with meta-analysis |
| Brogly, 2014[(27)](https://www.zotero.org/google-docs/?qOSWWy) | Does not adjust for the presence of a mental disorder |
| Bröms, 2023[(28)](https://www.zotero.org/google-docs/?1q7cna) | Not a systematic review with meta-analysis |
| Browne, 2006[(29)](https://www.zotero.org/google-docs/?lnYsiJ) | Does not investigate the association between any psychotropic medication use and any adverse outcome in pregnant individuals |
| Broy, 2010[(30)](https://www.zotero.org/google-docs/?195H5c) | Not a systematic review with meta-analysis |
| Burt, 2001[(31)](https://www.zotero.org/google-docs/?0iJyJ6) | Not a systematic review with meta-analysis |
| Byatt, 2013[(32)](https://www.zotero.org/google-docs/?SUsPxj) | Not a systematic review with meta-analysis |
| Camuñas Palacín, 2016[(33)](https://www.zotero.org/google-docs/?yKhpQ3) | Not a systematic review with meta-analysis |
| Chaudhry, 2014[(34)](https://www.zotero.org/google-docs/?7hBahK) | Not a systematic review with meta-analysis |
| Chen, 2014[(35)](https://www.zotero.org/google-docs/?3ee7lF) | Does not investigate the association between any psychotropic medication use and any adverse outcome in pregnant individuals |
| Chen, 2022[(36)](https://www.zotero.org/google-docs/?DNG5GM) | Does not investigate the association between any psychotropic medication use and any adverse outcome in pregnant individuals |
| Chisolm, 2016[(37)](https://www.zotero.org/google-docs/?JSGhLx) | Not a systematic review with meta-analysis |
| Cleary, 2010[(38)](https://www.zotero.org/google-docs/?kbQLhe) | Does not adjust for the presence of a mental disorder |
| Cohen, 1994[(39)](https://www.zotero.org/google-docs/?T5jqnP) | Not a systematic review with meta-analysis |
| Conradt, 2019[(40)](https://www.zotero.org/google-docs/?7UibWa) | Not a systematic review with meta-analysis |
| Coughlin, 2015[(41)](https://www.zotero.org/google-docs/?HQJfON) | Does not adjust for the presence of a mental disorder |
| Crawford-Faucher, 2010[(42)](https://www.zotero.org/google-docs/?FJXlx8) | Not a systematic review with meta-analysis |
| Creeley, 2019[(43)](https://www.zotero.org/google-docs/?qJMMG9) | Not a systematic review with meta-analysis |
| Cuomo, 2018[(44)](https://www.zotero.org/google-docs/?tCkt22) | Not a systematic review with meta-analysis |
| Damkier, 2018[(45)](https://www.zotero.org/google-docs/?9fq0Hx) | Not a systematic review with meta-analysis |
| Davidson, 2021[(46)](https://www.zotero.org/google-docs/?WRGDb9) | Not a systematic review with meta-analysis |
| Deligiannidis, 2014[(47)](https://www.zotero.org/google-docs/?kpC5Pz) | Not a systematic review with meta-analysis |
| Desaunay, 2023[(48)](https://www.zotero.org/google-docs/?9LZ3iG) | Does not adjust for the presence of a mental disorder |
| DeVries, 2021[(49)](https://www.zotero.org/google-docs/?ZBuzf9) | Does not adjust for the presence of a mental disorder |
| Dideriksen, 2013[(50)](https://www.zotero.org/google-docs/?MBf0B4) | Not a systematic review with meta-analysis |
| Donnelly, 2007[(51)](https://www.zotero.org/google-docs/?hGOodr) | Not a systematic review with meta-analysis |
| Eke, 2016[(52)](https://www.zotero.org/google-docs/?SpBQRJ) | Does not adjust for the presence of a mental disorder |
| Ellfolk, 2010[(53)](https://www.zotero.org/google-docs/?u7DzXH) | Not a systematic review with meta-analysis |
| El-Marroun, 2014[(54)](https://www.zotero.org/google-docs/?VnSXfC) | Not a systematic review with meta-analysis |
| Ennis, 2015[(55)](https://www.zotero.org/google-docs/?cYb1zL) | Not a systematic review with meta-analysis |
| Etemadi-Aleagha, 2022[(56)](https://www.zotero.org/google-docs/?RpfL2z) | Not a systematic review with meta-analysis |
| Fenger-Gron, 2011[(57)](https://www.zotero.org/google-docs/?BRFNB7) | Not a systematic review with meta-analysis |
| Forinash, 2010[(58)](https://www.zotero.org/google-docs/?rBrUqp) | Not a systematic review with meta-analysis |
| Fortinguerra, 2009[(59)](https://www.zotero.org/google-docs/?OSq4t3) | Not a systematic review with meta-analysis |
| Fuchs, 2020[(60)](https://www.zotero.org/google-docs/?kyWHdP) | Not a systematic review with meta-analysis |
| Galbally, 2010[(61)](https://www.zotero.org/google-docs/?Oq2rTP) | Not a systematic review with meta-analysis |
| Galbally, 2012[(62)](https://www.zotero.org/google-docs/?IM4WI1) | Not a systematic review with meta-analysis |
| Galbally, 2014[(63)](https://www.zotero.org/google-docs/?DN3RST) | Not a systematic review with meta-analysis |
| Gao, 2017[(64)](https://www.zotero.org/google-docs/?vF0jbp) | Does not adjust for the presence of a mental disorder |
| Gentile, 2007[(65)](https://www.zotero.org/google-docs/?4gfG6T) | Not a systematic review with meta-analysis |
| Gentile, 2007[(66)](https://www.zotero.org/google-docs/?JcovY1) | Not a systematic review with meta-analysis |
| Gentile, 2008[(67)](https://www.zotero.org/google-docs/?jfUtFe) | Not a systematic review with meta-analysis |
| Gentile, 2009[(68)](https://www.zotero.org/google-docs/?SAXFfv) | Not a systematic review with meta-analysis |
| Gentile, 2010[(69)](https://www.zotero.org/google-docs/?MGDjzx) | Not a systematic review with meta-analysis |
| Gentile, 2010[(70)](https://www.zotero.org/google-docs/?FY8LFd) | Not a systematic review with meta-analysis |
| Gentile, 2011[(71)](https://www.zotero.org/google-docs/?QYmH9W) | Not a systematic review with meta-analysis |
| Gentile, 2011[(72)](https://www.zotero.org/google-docs/?cevaMB) | Not a systematic review with meta-analysis |
| Gentile, 2014[(73)](https://www.zotero.org/google-docs/?GGpOfN) | Not a systematic review with meta-analysis |
| Gentile, 2014[(74)](https://www.zotero.org/google-docs/?2TsCnj) | Not a systematic review with meta-analysis |
| Gentile, 2014[(75)](https://www.zotero.org/google-docs/?bSd8jq) | Not a systematic review with meta-analysis |
| Gentile, 2015[(76)](https://www.zotero.org/google-docs/?GHhFbP) | Not a systematic review with meta-analysis |
| Gentile, 2015[(77)](https://www.zotero.org/google-docs/?Ae6DHf) | Not a systematic review with meta-analysis |
| Gentile, 2017[(78)](https://www.zotero.org/google-docs/?kjZgVs) | Not a systematic review with meta-analysis |
| Gentile, 2017[(79)](https://www.zotero.org/google-docs/?kRDPER) | Not a systematic review with meta-analysis |
| Gimenez, 2019[(80)](https://www.zotero.org/google-docs/?EKAQdA) | Not a systematic review with meta-analysis |
| Goldberg, 1994[(81)](https://www.zotero.org/google-docs/?B7UnAO) | Not a systematic review with meta-analysis |
| Goracci, 2015[(82)](https://www.zotero.org/google-docs/?hDKtDc) | Not a systematic review with meta-analysis |
| Grigoriadis, 2013[(83)](https://www.zotero.org/google-docs/?2uL8Ek) | Does not adjust for the presence of a mental disorder |
| Grigoriadis, 2013[(84)](https://www.zotero.org/google-docs/?S2y2sR) | Does not adjust for the presence of a mental disorder |
| Grigoriadis, 2014[(85)](https://www.zotero.org/google-docs/?7lWtaI) | Does not adjust for the presence of a mental disorder |
| Grigoriadis, 2014[(85)](https://www.zotero.org/google-docs/?TbgDZa) | Does not adjust for the presence of a mental disorder |
| Grove, 2018[(86)](https://www.zotero.org/google-docs/?Z8OFvR) | Does not adjust for the presence of a mental disorder |
| Guan, 2018[(87)](https://www.zotero.org/google-docs/?h4U5mX) | Does not adjust for the presence of a mental disorder |
| Gumusoglu, 2022[(88)](https://www.zotero.org/google-docs/?geG9pB) | Does not adjust for the presence of a mental disorder |
| Gutierrez-Alvarez, 2005[(89)](https://www.zotero.org/google-docs/?jbRNex) | Does not investigate the association between any psychotropic medication use and any adverse outcome in pregnant individuals |
| Haan, 2022[(90)](https://www.zotero.org/google-docs/?dSf5YL) | Does not investigate the association between any psychotropic medication use and any adverse outcome in pregnant individuals |
| Haskey, 2017[(91)](https://www.zotero.org/google-docs/?Ulb1Wr) | Not a systematic review with meta-analysis |
| Hemels, 2005[(92)](https://www.zotero.org/google-docs/?X4bU4f) | Does not adjust for the presence of a mental disorder |
| Hemmati, 2022[(93)](https://www.zotero.org/google-docs/?hFLokj) | Does not adjust for the presence of a mental disorder |
| Hendrick, 2017[(94)](https://www.zotero.org/google-docs/?wtEgE1) | Not a systematic review with meta-analysis |
| Hillemacher, 2021[(95)](https://www.zotero.org/google-docs/?3Rbwal) | Not a systematic review with meta-analysis |
| Hjorth, 2019[(96)](https://www.zotero.org/google-docs/?2U2x0c) | Does not investigate the association between any psychotropic medication use and any adverse outcome in pregnant individuals |
| Huang, 2014[(97)](https://www.zotero.org/google-docs/?7KzSqO) | Meta-analysis is not the largest available for exposure, population, control, and outcome |
| Hulse, 1997[(98)](https://www.zotero.org/google-docs/?To5eem) | Does not adjust for the presence of a mental disorder |
| Huntington, 2004[(99)](https://www.zotero.org/google-docs/?ch1ilE) | Not a systematic review with meta-analysis |
| Iqbal, 2002[(100)](https://www.zotero.org/google-docs/?Qf6kLl) | Not a systematic review with meta-analysis |
| Jain, 2005[(101)](https://www.zotero.org/google-docs/?KwGKKQ) | Not a systematic review with meta-analysis |
| Jarde, 2016[(102)](https://www.zotero.org/google-docs/?eXUg0l) | Does not investigate the association between any psychotropic medication use and any adverse outcome in pregnant individuals |
| Jentink, 2010[(103)](https://www.zotero.org/google-docs/?H5pRGw) | Not a systematic review with meta-analysis |
| Jiang, 2016[(104)](https://www.zotero.org/google-docs/?r181ex) | Does not adjust for the presence of a mental disorder |
| Jiang, 2018[(105)](https://www.zotero.org/google-docs/?RDFw4d) | Does not adjust for the presence of a mental disorder |
| Jiang, 2019[(106)](https://www.zotero.org/google-docs/?St7ehq) | Does not adjust for the presence of a mental disorder |
| Jin, 2021[(107)](https://www.zotero.org/google-docs/?J9I3LR) | Does not adjust for the presence of a mental disorder |
| Jones, 2012[(108)](https://www.zotero.org/google-docs/?Sbu3OK) | Not a systematic review with meta-analysis |
| Jones, 2013[(109)](https://www.zotero.org/google-docs/?E7dRiu) | Not a systematic review with meta-analysis |
| Kang, 2017[(110)](https://www.zotero.org/google-docs/?iV5nT0) | Does not adjust for the presence of a mental disorder |
| Kaplan, 2016[(111)](https://www.zotero.org/google-docs/?PpXhWS) | Does not adjust for the presence of a mental disorder |
| Kautzky, 2021[(112)](https://www.zotero.org/google-docs/?8W8PdY) | Meta-analysis provides insufficient data for quantitative synthesis |
| Kinsella, 2022[(113)](https://www.zotero.org/google-docs/?MbBQQ3) | Does not adjust for the presence of a mental disorder |
| Kiplagat, 2020[(114)](https://www.zotero.org/google-docs/?NfNjIM) | Not a systematic review with meta-analysis |
| Knight, 2021[(115)](https://www.zotero.org/google-docs/?ygvlue) | Not a systematic review with meta-analysis |
| Kobayashi, 2016[(116)](https://www.zotero.org/google-docs/?TS4PAr) | Meta-analysis is not the largest available for exposure, population, control, and outcome |
| Kong, 2018[(117)](https://www.zotero.org/google-docs/?lvPwKg) | Not a systematic review with meta-analysis |
| Kucukgoncu, 2020[(118)](https://www.zotero.org/google-docs/?Dr5SUJ) | Does not adjust for the presence of a mental disorder |
| Kuller, 1996[(119)](https://www.zotero.org/google-docs/?nfzlJY) | Not a systematic review with meta-analysis |
| Lassen, 2016[(120)](https://www.zotero.org/google-docs/?iec4EE) | Not a systematic review with meta-analysis |
| Lattimore, 2005[(121)](https://www.zotero.org/google-docs/?l3ZRpu) | Does not adjust for the presence of a mental disorder |
| Lee, 2020[(122)](https://www.zotero.org/google-docs/?owm4Xi) | Does not investigate the association between any psychotropic medication use and any adverse outcome in pregnant individuals |
| Leshem, 2021[(123)](https://www.zotero.org/google-docs/?ptMW8H) | Does not adjust for the presence of a mental disorder |
| Leshem, 2021[(123)](https://www.zotero.org/google-docs/?GvdBGp) | Does not adjust for the presence of a mental disorder |
| Leung, 2021[(124)](https://www.zotero.org/google-docs/?6RlRaY) | Does not adjust for the presence of a mental disorder |
| Li, 2015[(125)](https://www.zotero.org/google-docs/?O2Tyu1) | Does not investigate the association between any psychotropic medication use and any adverse outcome in pregnant individuals |
| Lind, 2017[(126)](https://www.zotero.org/google-docs/?NFKJ2m) | Not a systematic review with meta-analysis |
| Link, 2020[(127)](https://www.zotero.org/google-docs/?sh3A2g) | Does not adjust for the presence of a mental disorder |
| Liu, 2022[(128)](https://www.zotero.org/google-docs/?ruzSpN) | Does not investigate the association between any psychotropic medication use and any adverse outcome in pregnant individuals |
| Longo, 2009[(129)](https://www.zotero.org/google-docs/?6YEOAW) | Not a systematic review with meta-analysis |
| Lopez-Yarto, 2012[(130)](https://www.zotero.org/google-docs/?DDRKIS) | Does not adjust for the presence of a mental disorder |
| Lorenzo, 2014[(131)](https://www.zotero.org/google-docs/?zQBmpA) | Not a systematic review with meta-analysis |
| Lorenzo, 2014[(131)](https://www.zotero.org/google-docs/?h7tryF) | Not a systematic review with meta-analysis |
| Malm, 2012[(132)](https://www.zotero.org/google-docs/?EiFQqk) | Not a systematic review with meta-analysis |
| Man, 2015[(133)](https://www.zotero.org/google-docs/?g8Xc6i) | Does not adjust for the presence of a mental disorder |
| Man, 2018[(134)](https://www.zotero.org/google-docs/?HOfmkK) | Does not adjust for the presence of a mental disorder |
| Masarwa, 2019[(135)](https://www.zotero.org/google-docs/?lDBXgI) | Does not adjust for the presence of a mental disorder |
| Maslova, 2010[(136)](https://www.zotero.org/google-docs/?iKzKNB) | Does not investigate the association between any psychotropic medication use and any adverse outcome in pregnant individuals |
| Matalon, 2002[(137)](https://www.zotero.org/google-docs/?Z8aoXR) | Does not adjust for the presence of a mental disorder |
| Mathew, 2022[(138)](https://www.zotero.org/google-docs/?ZbWLQO) | Not a systematic review with meta-analysis |
| McCauley-Elsom, 2010[(139)](https://www.zotero.org/google-docs/?AaaSt8) | Not a systematic review with meta-analysis |
| McDonagh, 2014[(140)](https://www.zotero.org/google-docs/?9HBoFX) | Does not adjust for the presence of a mental disorder |
| Medveczky, 2004[(141)](https://www.zotero.org/google-docs/?kGiry8) | Not a systematic review with meta-analysis |
| Mehta, 2017[(142)](https://www.zotero.org/google-docs/?d61c39) | Not a systematic review with meta-analysis |
| Mezzacappa, 2017[(143)](https://www.zotero.org/google-docs/?1mJPWU) | Meta-analysis is not the largest available for exposure, population, control, and outcome |
| Misri, 2000[(144)](https://www.zotero.org/google-docs/?th9XRn) | Not a systematic review with meta-analysis |
| Mitchell, 2018[(145)](https://www.zotero.org/google-docs/?x6cPgb) | Not a systematic review with meta-analysis |
| Molenaar, 2020[(146)](https://www.zotero.org/google-docs/?7QB0Mf) | Does not investigate the association between any psychotropic medication use and any adverse outcome in pregnant individuals |
| Monnelly, 2019[(147)](https://www.zotero.org/google-docs/?rHYnYF) | Does not adjust for the presence of a mental disorder |
| Montouris, 2005[(148)](https://www.zotero.org/google-docs/?tFhlgW) | Does not investigate the association between any psychotropic medication use and any adverse outcome in pregnant individuals |
| Munk-Olsen, 2018[(149)](https://www.zotero.org/google-docs/?PgwKnw) | Does not adjust for the presence of a mental disorder |
| Myles, 2013[(150)](https://www.zotero.org/google-docs/?14y1nR) | Does not adjust for the presence of a mental disorder |
| Myung, 2012[(151)](https://www.zotero.org/google-docs/?5t4Ifw) | Does not adjust for the presence of a mental disorder |
| Nelson, 2020[(152)](https://www.zotero.org/google-docs/?eCxu0G) | Does not adjust for the presence of a mental disorder |
| Newmark, 2019[(153)](https://www.zotero.org/google-docs/?4pgknk) | Not a systematic review with meta-analysis |
| Nikfar, 2012[(154)](https://www.zotero.org/google-docs/?rvSYPv) | Does not adjust for the presence of a mental disorder |
| Noormohammadi, 2016[(155)](https://www.zotero.org/google-docs/?AgaNFf) | Not a systematic review with meta-analysis |
| Ordean, 2023[(156)](https://www.zotero.org/google-docs/?Y6M87E) | Not a systematic review with meta-analysis |
| Orsolini, 2015[(157)](https://www.zotero.org/google-docs/?YNeAjf) | Not a systematic review with meta-analysis |
| Orsolini, 2021[(158)](https://www.zotero.org/google-docs/?9kfmdV) | Not a systematic review with meta-analysis |
| Pacchiarotti, 2016[(159)](https://www.zotero.org/google-docs/?Kgflzj) | Does not adjust for the presence of a mental disorder |
| Painuly, 2013[(160)](https://www.zotero.org/google-docs/?ZvMYqL) | Does not adjust for the presence of a mental disorder |
| Pariente, 2017[(161)](https://www.zotero.org/google-docs/?DcS9rj) | Does not adjust for the presence of a mental disorder |
| Pedersen, 2014[(162)](https://www.zotero.org/google-docs/?MHX9aE) | Not a systematic review with meta-analysis |
| Petersen, 2016[(163)](https://www.zotero.org/google-docs/?KBLwNX) | Not a systematic review with meta-analysis |
| Pinheiro, 2015[(164)](https://www.zotero.org/google-docs/?tANDPi) | Does not investigate the association between any psychotropic medication use and any adverse outcome in pregnant individuals |
| Poels, 2018[(165)](https://www.zotero.org/google-docs/?ZUcydH) | Not a systematic review with meta-analysis |
| Prady, 2018[(166)](https://www.zotero.org/google-docs/?CNVGi4) | Not a systematic review with meta-analysis |
| Previti, 2014[(167)](https://www.zotero.org/google-docs/?Iwertu) | Not a systematic review with meta-analysis |
| Rahimi, 2006[(168)](https://www.zotero.org/google-docs/?WToicI) | Does not adjust for the presence of a mental disorder |
| Rais, 2014[(169)](https://www.zotero.org/google-docs/?7KF3og) | Does not adjust for the presence of a mental disorder |
| Rausgaard, 2020[(170)](https://www.zotero.org/google-docs/?jBUwzq) | Not a systematic review with meta-analysis |
| Reising, 2019[(171)](https://www.zotero.org/google-docs/?q81Ns2) | Not a systematic review with meta-analysis |
| Rhee, 2015[(172)](https://www.zotero.org/google-docs/?ehFC1U) | Does not investigate the association between any psychotropic medication use and any adverse outcome in pregnant individuals |
| Ricks, 2017[(173)](https://www.zotero.org/google-docs/?lT8Fdx) | Not a systematic review with meta-analysis |
| Riggin, 2013[(174)](https://www.zotero.org/google-docs/?y2KpIA) | Does not adjust for the presence of a mental disorder |
| Rubinchik, 2005[(175)](https://www.zotero.org/google-docs/?GZ9DM6) | Not a systematic review with meta-analysis |
| Ruisch, 2018[(176)](https://www.zotero.org/google-docs/?FKnu4h) | Does not investigate the association between any psychotropic medication use and any adverse outcome in pregnant individuals |
| Ryan, 2023[(177)](https://www.zotero.org/google-docs/?9R8spi) | Not a systematic review with meta-analysis |
| Santone, 2009[(178)](https://www.zotero.org/google-docs/?rBq1Tj) | Not a systematic review with meta-analysis |
| Schmidt, 2022[(179)](https://www.zotero.org/google-docs/?tyAkZ0) | Does not investigate the association between any psychotropic medication use and any adverse outcome in pregnant individuals |
| Schmidt, 2023[(179)](https://www.zotero.org/google-docs/?1Ii01z) | Does not investigate the association between any psychotropic medication use and any adverse outcome in pregnant individuals |
| Schoretsanitis, 2020[(180)](https://www.zotero.org/google-docs/?oDJ5pi) | Does not investigate the association between any psychotropic medication use and any adverse outcome in pregnant individuals |
| Scialli, 2010[(181)](https://www.zotero.org/google-docs/?MonaP7) | Not a systematic review with meta-analysis |
| Seeman, 2012[(182)](https://www.zotero.org/google-docs/?3oUE3t) | Not a systematic review with meta-analysis |
| Sharma, 2012[(183)](https://www.zotero.org/google-docs/?oyr0pf) | Not a systematic review with meta-analysis |
| Shen, 2017[(184)](https://www.zotero.org/google-docs/?8KsqI9) | Does not adjust for the presence of a mental disorder |
| Simoncelli, 2010[(185)](https://www.zotero.org/google-docs/?TPWHXo) | Not a systematic review with meta-analysis |
| Suri, 2014[(186)](https://www.zotero.org/google-docs/?538Ymw) | Not a systematic review with meta-analysis |
| Tak, 2017[(187)](https://www.zotero.org/google-docs/?BRxgA5) | Not a systematic review with meta-analysis |
| Tanoshima, 2015[(188)](https://www.zotero.org/google-docs/?PEq1qE) | Does not adjust for the presence of a mental disorder |
| Taylor, 2021[(189)](https://www.zotero.org/google-docs/?WAeT5i) | Meta-analysis provides insufficient data for quantitative synthesis |
| Terrana, 2015[(190)](https://www.zotero.org/google-docs/?VmTmCU) | Does not adjust for the presence of a mental disorder |
| Thanigaivel, 2021[(191)](https://www.zotero.org/google-docs/?DC8zaC) | Does not adjust for the presence of a mental disorder |
| Thanigaivel, 2022[(191)](https://www.zotero.org/google-docs/?rQg0aW) | Not a systematic review with meta-analysis |
| Thormahlen, 2006[(192)](https://www.zotero.org/google-docs/?kxpTTy) | Not a systematic review with meta-analysis |
| Tillery, 2013[(193)](https://www.zotero.org/google-docs/?MsNcCH) | Not a systematic review with meta-analysis |
| Tosato, 2017[(194)](https://www.zotero.org/google-docs/?u9X1wT) | Not a systematic review with meta-analysis |
| Towers, 2020[(195)](https://www.zotero.org/google-docs/?xbYFL0) | Does not adjust for the presence of a mental disorder |
| Tuccori, 2009[(196)](https://www.zotero.org/google-docs/?Tzg9ye) | Not a systematic review with meta-analysis |
| Tuccori, 2009[(196)](https://www.zotero.org/google-docs/?dD9QCT) | Not a systematic review with meta-analysis |
| Tuccori, 2010[(197)](https://www.zotero.org/google-docs/?dqfhNs) | Not a systematic review with meta-analysis |
| Turner, 2019[(198)](https://www.zotero.org/google-docs/?4LdFQY) | Does not adjust for the presence of a mental disorder |
| Udechuku, 2010[(199)](https://www.zotero.org/google-docs/?0lTwRU) | Not a systematic review with meta-analysis |
| Uguz, 2016[(200)](https://www.zotero.org/google-docs/?UEAStE) | Not a systematic review with meta-analysis |
| Uguz, 2017[(201)](https://www.zotero.org/google-docs/?8RWKPa) | Not a systematic review with meta-analysis |
| Uguz, 2018[(202)](https://www.zotero.org/google-docs/?dAo7Gf) | Not a systematic review with meta-analysis |
| Uguz, 2019[(203)](https://www.zotero.org/google-docs/?zpFvbP) | Not a systematic review with meta-analysis |
| Uguz, 2019[(204)](https://www.zotero.org/google-docs/?QIMQKX) | Not a systematic review with meta-analysis |
| Uguz, 2020[(205)](https://www.zotero.org/google-docs/?DgH52p) | Not a systematic review with meta-analysis |
| Uguz, 2020[(206)](https://www.zotero.org/google-docs/?VtoC9B) | Not a systematic review with meta-analysis |
| Uguz, 2021[(207)](https://www.zotero.org/google-docs/?hjTrED) | Not a systematic review with meta-analysis |
| Uguz, 2021[(208)](https://www.zotero.org/google-docs/?prOJX3) | Not a systematic review with meta-analysis |
| Urato, 2014[(209)](https://www.zotero.org/google-docs/?lWC5lD) | Not a systematic review with meta-analysis |
| vanDriel, 2010[(210)](https://www.zotero.org/google-docs/?UOrsdw) | Not a systematic review with meta-analysis |
| Viswanathan, 2021[(211)](https://www.zotero.org/google-docs/?UCFRQ4) | Not a systematic review with meta-analysis |
| Vitale, 2016[(212)](https://www.zotero.org/google-docs/?6UaT4c) | Not a systematic review with meta-analysis |
| Viuff, 2016[(213)](https://www.zotero.org/google-docs/?BQHCJH) | Not a systematic review with meta-analysis |
| Vlenterie, 2021[(214)](https://www.zotero.org/google-docs/?qZaJWy) | Does not adjust for the presence of a mental disorder |
| Wang, 2015[(215)](https://www.zotero.org/google-docs/?t1w2SH) | Does not adjust for the presence of a mental disorder |
| Wang, 2021[(216)](https://www.zotero.org/google-docs/?zoBL2C) | Does not adjust for the presence of a mental disorder |
| Wang, 2021[(217)](https://www.zotero.org/google-docs/?ak5FcM) | Does not adjust for the presence of a mental disorder |
| Wang, 2021[(218)](https://www.zotero.org/google-docs/?fUO4jD) | Not a systematic review with meta-analysis |
| Wang, 2021[(219)](https://www.zotero.org/google-docs/?1J9NKu) | Does not adjust for the presence of a mental disorder |
| Wang, 2022[(220)](https://www.zotero.org/google-docs/?Y3kOI6) | Does not adjust for the presence of a mental disorder |
| Wen, 2004[(221)](https://www.zotero.org/google-docs/?sP0V3a) | Not a systematic review with meta-analysis |
| Wen, 2004[(222)](https://www.zotero.org/google-docs/?r5jyqR) | Not a systematic review with meta-analysis |
| Wesseloo, 2019[(223)](https://www.zotero.org/google-docs/?W0bt8F) | Not a systematic review with meta-analysis |
| Wisner, 1999[(224)](https://www.zotero.org/google-docs/?kV5U3M) | Not a systematic review with meta-analysis |
| Wisner, 1999[(224)](https://www.zotero.org/google-docs/?XHSSKh) | Not a systematic review with meta-analysis |
| Wurst, 2010[(225)](https://www.zotero.org/google-docs/?pDl8J3) | Does not adjust for the presence of a mental disorder |
| Yamaguchi, 2008[(226)](https://www.zotero.org/google-docs/?0jtLwq) | Not a systematic review with meta-analysis |
| Zedler, 2016[(227)](https://www.zotero.org/google-docs/?lTHudz) | Does not adjust for the presence of a mental disorder |
| Zhang, 2017[(228)](https://www.zotero.org/google-docs/?J4LZoZ) | Does not adjust for the presence of a mental disorder |
| Zhao, 2020[(229)](https://www.zotero.org/google-docs/?v8Uvb9) | Not a systematic review with meta-analysis |
| Zheng, 2022[(230)](https://www.zotero.org/google-docs/?1Q6r2Z) | Does not investigate the association between any psychotropic medication use and any adverse outcome in pregnant individuals |
| Zusman, 2022[(231)](https://www.zotero.org/google-docs/?MCmfVB) | Meta-analysis provides insufficient data for quantitative synthesis |
| Zwink, 2018[(232)](https://www.zotero.org/google-docs/?9pFC6B) | Does not adjust for the presence of a mental disorder |

**References**

.

[1. ‘t Jong GW, Einarson T, Koren G, Einarson A. Antidepressant use in pregnancy and persistent pulmonary hypertension of the newborn (PPHN): A systematic review. Reprod Toxicol. 2012 Nov 1;34(3):293–7.](https://www.zotero.org/google-docs/?g5hoFp)

[2. Addis A, Koren G. Safety of fluoxetine during the first trimester of pregnancy: a meta-analytical review of epidemiological studies. Psychol Med. 2000 Jan;30(1):89–94.](https://www.zotero.org/google-docs/?g5hoFp)

[3. Andalib S, Emamhadi MR, Yousefzadeh-Chabok S, Shakouri SK, Høilund-Carlsen PF, Vafaee MS, et al. Maternal SSRI exposure increases the risk of autistic offspring: A meta-analysis and systematic review. Eur Psychiatry. 2017 Sep;45:161–6.](https://www.zotero.org/google-docs/?g5hoFp)

[4. Andrade C. Antidepressant Exposure During Pregnancy and Risk of Autism in the Offspring, 1: Meta-Review of Meta-Analyses. J Clin Psychiatry. 2017 Sep 26;78(8):905.](https://www.zotero.org/google-docs/?g5hoFp)

[5. Andrade C. Adverse Pregnancy Outcomes Associated With Gestational Exposure to Antiepileptic Drugs. J Clin Psychiatry. 2018 Jul 31;79(4):619.](https://www.zotero.org/google-docs/?g5hoFp)

[6. Andrade C. Gestational Exposure to Benzodiazepines, 2: The Risk of Congenital Malformations Examined Through the Prism of Compatibility Intervals. J Clin Psychiatry. 2019 Oct 1;80(5):7813.](https://www.zotero.org/google-docs/?g5hoFp)

[7. Andrade C. Gestational Exposure to Benzodiazepines, 3: Clobazam and Major Congenital Malformations. J Clin Psychiatry. 2019 Nov 26;80(6):7816.](https://www.zotero.org/google-docs/?g5hoFp)

[8. Andrade C. Major Congenital Malformations Associated With Exposure to Second-Generation Antipsychotic Drugs During Pregnancy. J Clin Psychiatry. 2021 Oct 5;82(5):37288.](https://www.zotero.org/google-docs/?g5hoFp)

[9. Andrade C. Selective Serotonin Reuptake Inhibitor Use in Pregnancy and Risk of Postpartum Hemorrhage. J Clin Psychiatry. 2022 Apr 4;83(2):40446.](https://www.zotero.org/google-docs/?g5hoFp)

[10. Lithium during pregnancy: malformations, fetotoxicity and uncertain long-term effects. Prescrire Int. 2020;29(14):97–9.](https://www.zotero.org/google-docs/?g5hoFp)

[11. Ltd BPG. Lithium in pregnancy. Drug Ther Bull. 2021 Jan 1;59(1):5–5.](https://www.zotero.org/google-docs/?g5hoFp)

[12. Araujo JSA de, Delgado IF, Paumgartten FJR. Antenatal exposure to antidepressant drugs and the risk of neurodevelopmental and psychiatric disorders: a systematic review. Cad Saúde Pública. 2020 Jan 31;36:e00026619.](https://www.zotero.org/google-docs/?g5hoFp)

[13. Athar F, Ehsan M, Farooq M, Lo KB, Cheema HA, Ahmad S, et al. Adverse fetal and neonatal outcomes following in-utero exposure to oxcarbazepine: A systematic review and meta-analysis. Br J Clin Pharmacol. 2022;88(8):3600–9.](https://www.zotero.org/google-docs/?g5hoFp)

[14. Mitchell PB, Austin MPV. Psychotropic medications in pregnant women: treatment dilemmas. Med J Aust. 1998;169(8):428–31.](https://www.zotero.org/google-docs/?g5hoFp)

[15. Balalian AA, Graeve R, Richter M, Fink A, Kielstein H, Martins SS, et al. Prenatal exposure to opioids and neurodevelopment in infancy and childhood: A systematic review. Front Pediatr [Internet]. 2023 [cited 2024 Jan 4];11. Available from: https://www.frontiersin.org/articles/10.3389/fped.2023.1071889](https://www.zotero.org/google-docs/?g5hoFp)

[16. Baldacchino A, Arbuckle K, Petrie DJ, McCowan C. Neurobehavioral consequences of chronic intrauterine opioid exposure in infants and preschool children: a systematic review and meta-analysis. BMC Psychiatry. 2014 Apr 8;14(1):104.](https://www.zotero.org/google-docs/?g5hoFp)

[17. Banti S, Borri C, Camilleri V, Cortopassi C, Ramacciotti D, Rambelli C, et al. Perinatal mood and anxiety disorders. Clinical assessment and management. A review of current literature Disturbi dell’umore e disturbi d’ansia perinatali. Valutazione clinica e gestione del trattamento. Una revisione attuale della letteratura corrente. In 2009 [cited 2024 Jan 4]. Available from: https://www.semanticscholar.org/paper/Perinatal-mood-and-anxiety-disorders.-Clinical-and-Banti-Borri/00ab83f3c8e6ba4177625cc63bc32d1426e07ee3](https://www.zotero.org/google-docs/?g5hoFp)

[18. Bar-Oz B, Einarson T, Einarson A, Boskovic R, O’Brien L, Malm H, et al. Paroxetine and congenital malformations: Meta-Analysis and consideration of potential confounding factors. Clin Ther. 2007 May 1;29(5):918–26.](https://www.zotero.org/google-docs/?g5hoFp)

[19. Beex-Oosterhuis MM, Gool ARV, Heerdink ER, Kesteren C van, Marum RJ van. Clozapine Treatment During Pregnancy and the Postpartum Period: A Systematic Literature Review. J Clin Psychiatry. 2021 Dec 14;83(1):38713.](https://www.zotero.org/google-docs/?g5hoFp)

[20. Bellantuono C, Migliarese G, Gentile S. Serotonin reuptake inhibitors in pregnancy and the risk of major malformations: a systematic review. Hum Psychopharmacol Clin Exp. 2007 Apr;22(3):121–8.](https://www.zotero.org/google-docs/?g5hoFp)

[21. Bellantuono C, Bozzi F, Orsolini L, Catena-Dell’Osso M. The safety of escitalopram during pregnancy and breastfeeding: a comprehensive review. Hum Psychopharmacol Clin Exp. 2012;27(6):534–9.](https://www.zotero.org/google-docs/?g5hoFp)

[22. Bellantuono C, Vargas M, Mandarelli G, Nardi B, Martini MG. The safety of serotonin–noradrenaline reuptake inhibitors (SNRIs) in pregnancy and breastfeeding: a comprehensive review. Hum Psychopharmacol Clin Exp. 2015;30(3):143–51.](https://www.zotero.org/google-docs/?g5hoFp)

[23. Berwaerts K, Sienaert P, De Fruyt J. [Teratogenic effects of lamotrigine in women with bipolar disorder]. Tijdschr Voor Psychiatr. 2009;51(10):741–50.](https://www.zotero.org/google-docs/?g5hoFp)

[24. Besag FMC, Vasey MJ. Should Antidepressants be Avoided in Pregnancy? Drug Saf. 2023 Jan 1;46(1):1–17.](https://www.zotero.org/google-docs/?g5hoFp)

[25. Blanc J, Tosello B, Ekblad MO, Berlin I, Netter A. Nicotine Replacement Therapy during Pregnancy and Child Health Outcomes: A Systematic Review. Int J Environ Res Public Health. 2021 Jan;18(8):4004.](https://www.zotero.org/google-docs/?g5hoFp)

[26. Bolea-Alamanac BM, Green A, Verma G, Maxwell P, Davies SJC. Methylphenidate use in pregnancy and lactation: a systematic review of evidence. Br J Clin Pharmacol. 2014;77(1):96–101.](https://www.zotero.org/google-docs/?g5hoFp)

[27. Brogly SB, Saia KA, Walley AY, Du HM, Sebastiani P. Prenatal Buprenorphine Versus Methadone Exposure and Neonatal Outcomes: Systematic Review and Meta-Analysis. Am J Epidemiol. 2014 Oct 1;180(7):673–86.](https://www.zotero.org/google-docs/?g5hoFp)

[28. Bröms G, Hernandez-Diaz S, Huybrechts KF, Bateman BT, Kristiansen EB, Einarsdóttir K, et al. Atomoxetine in Early Pregnancy and the Prevalence of Major Congenital Malformations: A Multinational Study. J Clin Psychiatry. 2023 Jan 16;84(1):45077.](https://www.zotero.org/google-docs/?g5hoFp)

[29. Browne ML. Maternal Exposure to Caffeine and Risk of Congenital Anomalies: A Systematic Review. Epidemiology. 2006 May;17(3):324–31.](https://www.zotero.org/google-docs/?g5hoFp)

[30. Broy P, Bérard A. Gestational exposure to antidepressants and the risk of spontaneous abortion: a review. Curr Drug Deliv. 2010 Jan;7(1):76–92.](https://www.zotero.org/google-docs/?g5hoFp)

[31. Burt VK, Suri R, Altshuler L, Stowe Z, Hendrick VC, Muntean E. The Use of Psychotropic Medications During Breast-Feeding. Am J Psychiatry. 2001 Jul;158(7):1001–9.](https://www.zotero.org/google-docs/?g5hoFp)

[32. Byatt N, Deligiannidis KM, Freeman MP. Antidepressant use in pregnancy: a critical review focused on risks and controversies. Acta Psychiatr Scand. 2013;127(2):94–114.](https://www.zotero.org/google-docs/?g5hoFp)

[33. Camuñas Palacín A, Grigg J, Gilbert H, Worsley R, Gavrilidis E, Kulkarni J. Seguridad de los antipsicóticos atípicos en el embarazo. Psiquiatr Biológica. 2016 Jan 1;23(1):23–8.](https://www.zotero.org/google-docs/?g5hoFp)

[34. Chaudhry SA, Jong G, Koren G. The fetal safety of Levetiracetam: A systematic review. Reprod Toxicol. 2014 Jul 1;46:40–5.](https://www.zotero.org/google-docs/?g5hoFp)

[35. Chen LW, Wu Y, Neelakantan N, Chong MFF, Pan A, van Dam RM. Maternal caffeine intake during pregnancy is associated with risk of low birth weight: a systematic review and dose-response meta-analysis. BMC Med. 2014 Sep 19;12(1):174.](https://www.zotero.org/google-docs/?g5hoFp)

[36. Chen B, Zhang M, He Y, Si Y, Shi Y, Jiang K, et al. The association between caffeine exposure during pregnancy and risk of gestational hypertension/preeclampsia: A meta-analysis and systematical review. J Obstet Gynaecol Res. 2022;48(12):3045–55.](https://www.zotero.org/google-docs/?g5hoFp)

[37. Chisolm MS, Payne JL. Management of psychotropic drugs during pregnancy. BMJ. 2016 Jan 20;352:h5918.](https://www.zotero.org/google-docs/?g5hoFp)

[38. Cleary BJ, Donnelly J, Strawbridge J, Gallagher PJ, Fahey T, Clarke M, et al. Methadone dose and neonatal abstinence syndrome—systematic review and meta-analysis. Addiction. 2010;105(12):2071–84.](https://www.zotero.org/google-docs/?g5hoFp)

[39. Cohen LS, Friedman JM, Jefferson JW, Johnson EM, Weiner ML. A Reevaluation of Risk of In Utero Exposure to Lithium. JAMA. 1994 Jan 12;271(2):146–50.](https://www.zotero.org/google-docs/?g5hoFp)

[40. Conradt E, Flannery T, Aschner JL, Annett RD, Croen LA, Duarte CS, et al. Prenatal Opioid Exposure: Neurodevelopmental Consequences and Future Research Priorities. Pediatrics. 2019 Sep 1;144(3):e20190128.](https://www.zotero.org/google-docs/?g5hoFp)

[41. Coughlin CG, Blackwell KA, Bartley C, Hay M, Yonkers KA, Bloch MH. Obstetric and Neonatal Outcomes After Antipsychotic Medication Exposure in Pregnancy. Obstet Gynecol. 2015 May;125(5):1224.](https://www.zotero.org/google-docs/?g5hoFp)

[42. Crawford-Faucher A. Safety of Psychotropic Medications in Breastfeeding. Am Fam Physician. 2010 Jun 1;81(11):1369–70.](https://www.zotero.org/google-docs/?g5hoFp)

[43. Creeley CE, Denton LK. Use of Prescribed Psychotropics during Pregnancy: A Systematic Review of Pregnancy, Neonatal, and Childhood Outcomes. Brain Sci. 2019 Sep;9(9):235.](https://www.zotero.org/google-docs/?g5hoFp)

[44. Cuomo A, Goracci A, Fagiolini A. Aripiprazole use during pregnancy, peripartum and lactation. A systematic literature search and review to inform clinical practice. J Affect Disord. 2018 Mar 1;228:229–37.](https://www.zotero.org/google-docs/?g5hoFp)

[45. Damkier P, Videbech P. The Safety of Second-Generation Antipsychotics During Pregnancy: A Clinically Focused Review. CNS Drugs. 2018 Apr 1;32(4):351–66.](https://www.zotero.org/google-docs/?g5hoFp)

[46. Davidson AD, Bhat A, Chu F, Rice JN, Nduom NA, Cowley DS. A systematic review of the use of prazosin in pregnancy and lactation. Gen Hosp Psychiatry. 2021 Jul 1;71:134–6.](https://www.zotero.org/google-docs/?g5hoFp)

[47. Deligiannidis KM, Byatt N, Freeman MP. Pharmacotherapy for Mood Disorders in Pregnancy: A Review of Pharmacokinetic Changes and Clinical Recommendations for Therapeutic Drug Monitoring. J Clin Psychopharmacol. 2014 Apr;34(2):244.](https://www.zotero.org/google-docs/?g5hoFp)

[48. Desaunay P, Eude LG, Dreyfus M, Alexandre C, Fedrizzi S, Alexandre J, et al. Benefits and Risks of Antidepressant Drugs During Pregnancy: A Systematic Review of Meta-analyses. Pediatr Drugs. 2023 May 1;25(3):247–65.](https://www.zotero.org/google-docs/?g5hoFp)

[49. De Vries C, Gadzhanova S, Sykes MJ, Ward M, Roughead E. A Systematic Review and Meta-Analysis Considering the Risk for Congenital Heart Defects of Antidepressant Classes and Individual Antidepressants. Drug Saf. 2021 Mar 1;44(3):291–312.](https://www.zotero.org/google-docs/?g5hoFp)

[50. Dideriksen D, Pottegård A, Hallas J, Aagaard L, Damkier P. First Trimester In Utero Exposure to Methylphenidate. Basic Clin Pharmacol Toxicol. 2013;112(2):73–6.](https://www.zotero.org/google-docs/?g5hoFp)

[51. Donnelly A, Paton C. Safety of selective serotonin reuptake inhibitors in pregnancy. Psychiatr Bull. 2007 May;31(5):183–6.](https://www.zotero.org/google-docs/?g5hoFp)

[52. Eke A, Saccone G, Berghella V. Selective serotonin reuptake inhibitor (SSRI) use during pregnancy and risk of preterm birth: a systematic review and meta-analysis. BJOG Int J Obstet Gynaecol. 2016;123(12):1900–7.](https://www.zotero.org/google-docs/?g5hoFp)

[53. Ellfolk M, Malm H. Risks associated with in utero and lactation exposure to selective serotonin reuptake inhibitors (SSRIs). Reprod Toxicol. 2010 Sep 1;30(2):249–60.](https://www.zotero.org/google-docs/?g5hoFp)

[54. El Marroun H, White T, Verhulst FC, Tiemeier H. Maternal use of antidepressant or anxiolytic medication during pregnancy and childhood neurodevelopmental outcomes: a systematic review. Eur Child Adolesc Psychiatry. 2014 Oct 1;23(10):973–92.](https://www.zotero.org/google-docs/?g5hoFp)

[55. Ennis ZN, Damkier P. Pregnancy Exposure to Olanzapine, Quetiapine, Risperidone, Aripiprazole and Risk of Congenital Malformations. A Systematic Review. Basic Clin Pharmacol Toxicol. 2015;116(4):315–20.](https://www.zotero.org/google-docs/?g5hoFp)

[56. Etemadi-Aleagha A, Akhgari M. Psychotropic drug abuse in pregnancy and its impact on child neurodevelopment: A review. World J Clin Pediatr. 2022 Jan 9;11(1):1–13.](https://www.zotero.org/google-docs/?g5hoFp)

[57. Fenger-Grøn J, Thomsen M, Andersen KS, Nielsen RG. Paediatric outcomes following intrauterine exposure to serotonin reuptake inhibitors: a systematic review. Dan Med Bull. 2011 Sep;58(9):A4303.](https://www.zotero.org/google-docs/?g5hoFp)

[58. Forinash AB, Pitlick JM, Clark K, Alstat V. Nicotine Replacement Therapy Effect on Pregnancy Outcomes. Ann Pharmacother. 2010 Nov 1;44(11):1817–21.](https://www.zotero.org/google-docs/?g5hoFp)

[59. Fortinguerra F, Clavenna A, Bonati M. Psychotropic Drug Use During Breastfeeding: A Review of the Evidence. Pediatrics. 2009 Oct 1;124(4):e547–56.](https://www.zotero.org/google-docs/?g5hoFp)

[60. Fuchs C, Bridler R. Lithium während der Schwanger­schaft und in der postpartalen                    ­Phase bei Frauen mit einer bipolaren Störung – eine Metaanalyse                    zur Wirksamkeit und Sicherheit. Praxis. 2020 May;109(7):551–2.](https://www.zotero.org/google-docs/?g5hoFp)

[61. Galbally M, Roberts M, Buist A, Perinatal Psychotropic Review Group. Mood stabilizers in pregnancy: a systematic review. Aust N Z J Psychiatry. 2010 Nov;44(11):967–77.](https://www.zotero.org/google-docs/?g5hoFp)

[62. Galbally M, Lewis A, Gentile S, Walker S, Buist A. The biology of fetal exposure to serotonin reuptake inhibitors: Implications for neurodevelopment. In: Antidepressants: Pharmacology, Health Effects and Controversy. 2012.](https://www.zotero.org/google-docs/?g5hoFp)

[63. Galbally M, Snellen M, Power J. Antipsychotic drugs in pregnancy: a review of their maternal and fetal effects. Ther Adv Drug Saf. 2014 Apr 1;5(2):100–9.](https://www.zotero.org/google-docs/?g5hoFp)

[64. Gao SY, Wu QJ, Zhang TN, Shen ZQ, Liu CX, Xu X, et al. Fluoxetine and congenital malformations: a systematic review and meta-analysis of cohort studies. Br J Clin Pharmacol. 2017;83(10):2134–47.](https://www.zotero.org/google-docs/?g5hoFp)

[65. Gentile S. Serotonin Reuptake Inhibitor-Induced Perinatal Complications: Pediatr Drugs. 2007;9(2):97–106.](https://www.zotero.org/google-docs/?g5hoFp)

[66. Gentile S, Rossi A, Bellantuono C. SSRIs during breastfeeding: spotlight on milk-to-plasma ratio. Arch Womens Ment Health. 2007 Apr 1;10(2):39–51.](https://www.zotero.org/google-docs/?g5hoFp)

[67. Gentile S. Infant safety with antipsychotic therapy in breast-feeding: a systematic review. J Clin Psychiatry. 2008 Apr;69(4):666–73.](https://www.zotero.org/google-docs/?g5hoFp)

[68. Gentile S, Bellantuono C. Selective Serotonin Reuptake Inhibitor Exposure During Early Pregnancy and the Risk of Fetal Major Malformations: Focus on Paroxetine. J Clin Psychiatry. 2009 Feb 24;70(3):19161.](https://www.zotero.org/google-docs/?g5hoFp)

[69. Gentile S. On Categorizing Gestational, Birth, and Neonatal Complications Following Late Pregnancy Exposure to Antidepressants: The Prenatal Antidepressant Exposure Syndrome. CNS Spectr. 2010 Mar;15(3):167–85.](https://www.zotero.org/google-docs/?g5hoFp)

[70. Gentile S. Neurodevelopmental effects of prenatal exposure to psychotropic medications. Depress Anxiety. 2010;27(7):675–86.](https://www.zotero.org/google-docs/?g5hoFp)

[71. Gentile S, Galbally M. Prenatal exposure to antidepressant medications and neurodevelopmental outcomes: A systematic review. J Affect Disord. 2011 Jan 1;128(1):1–9.](https://www.zotero.org/google-docs/?g5hoFp)

[72. Gentile S. Selective serotonin reuptake inhibitor exposure during early pregnancy and the risk of birth defects. Acta Psychiatr Scand. 2011;123(4):266–75.](https://www.zotero.org/google-docs/?g5hoFp)

[73. Gentile S. Pregnancy exposure to second-generation antipsychotics and the risk of gestational diabetes. Expert Opin Drug Saf. 2014 Dec 1;13(12):1583–90.](https://www.zotero.org/google-docs/?g5hoFp)

[74. Gentile S. Risks of neurobehavioral teratogenicity associated with prenatal exposure to valproate monotherapy: a systematic review with regulatory repercussions. CNS Spectr. 2014 Aug;19(4):305–15.](https://www.zotero.org/google-docs/?g5hoFp)

[75. Gentile S. A safety evaluation of aripiprazole for treating schizophrenia during pregnancy and puerperium. Expert Opin Drug Saf. 2014 Dec 1;13(12):1733–42.](https://www.zotero.org/google-docs/?g5hoFp)

[76. Gentile S. Prenatal antidepressant exposure and the risk of autism spectrum disorders in children. Are we looking at the fall of Gods? J Affect Disord. 2015 Aug 15;182:132–7.](https://www.zotero.org/google-docs/?g5hoFp)

[77. Gentile S. Early pregnancy exposure to selective serotonin reuptake inhibitors, risks of major structural malformations, and hypothesized teratogenic mechanisms. Expert Opin Drug Metab Toxicol. 2015 Oct 3;11(10):1585–97.](https://www.zotero.org/google-docs/?g5hoFp)

[78. Gentile S, Fusco ML. Placental and fetal effects of antenatal exposure to antidepressants or untreated maternal depression. J Matern Fetal Neonatal Med. 2017 May 19;30(10):1189–99.](https://www.zotero.org/google-docs/?g5hoFp)

[79. Gentile S, Fusco ML. Neurodevelopmental outcomes in infants exposed in utero to antipsychotics: a systematic review of published data. CNS Spectr. 2017 Jun;22(3):273–81.](https://www.zotero.org/google-docs/?g5hoFp)

[80. Giménez A, Pacchiarotti I, Gil J, Murru A, Gomes SP, Pinzón JE, et al. Adverse outcomes during pregnancy and major congenital malformations in infants of patients with bipolar and schizoaffective disorders treated with antiepileptic drugs: A systematic review. Psychiatr Pol. 2019 Apr 30;53(2):223–44.](https://www.zotero.org/google-docs/?g5hoFp)

[81. Goldberg HL, Nissim R. Psychotropic Drugs in Pregnancy and Lactation. Int J Psychiatry Med. 1994 Jun 1;24(2):129–47.](https://www.zotero.org/google-docs/?g5hoFp)

[82. Goracci A, Valdagno M, Maltinti E, Sillari S, Fagiolini A. Benefici e potenziali rischi dell’utilizzo di antidepressivi in gravidanza: una revisione della letteratura. Riv Psichiatr. 2015 May 1;50(3):118–26.](https://www.zotero.org/google-docs/?g5hoFp)

[83. Grigoriadis S, VonderPorten EH, Mamisashvili L, Eady A, Tomlinson G, Dennis CL, et al. The Effect of Prenatal Antidepressant Exposure on Neonatal Adaptation: A Systematic Review and Meta-Analysis. J Clin Psychiatry. 2013 Apr 15;74(4):5650.](https://www.zotero.org/google-docs/?g5hoFp)

[84. Grigoriadis S, VonderPorten EH, Mamisashvili L, Roerecke M, Rehm J, Dennis CL, et al. Antidepressant Exposure During Pregnancy and Congenital Malformations: Is There an Association? A Systematic Review and Meta-Analysis of the Best Evidence. J Clin Psychiatry. 2013 Apr 15;74(4):903.](https://www.zotero.org/google-docs/?g5hoFp)

[85. Grigoriadis S, VonderPorten EH, Mamisashvili L, Tomlinson G, Dennis CL, Koren G, et al. Prenatal Exposure to Antidepressants and Persistent Pulmonary Hypertension of the Newborn: Systematic Review and Meta-analysis. Obstet Gynecol Surv. 2014 May;69(5):239.](https://www.zotero.org/google-docs/?g5hoFp)

[86. Grove K, Lewis AJ, Galbally M. Prenatal Antidepressant Exposure and Child Motor Development: A Meta-analysis. Pediatrics. 2018 Jul 1;142(1):e20180356.](https://www.zotero.org/google-docs/?g5hoFp)

[87. Guan HB, Wei Y, Wang LL, Qiao C, Liu CX. Prenatal Selective Serotonin Reuptake Inhibitor Use and Associated Risk for Gestational Hypertension and Preeclampsia: A Meta-Analysis of Cohort Studies. J Womens Health. 2018 Jun;27(6):791–800.](https://www.zotero.org/google-docs/?g5hoFp)

[88. Gumusoglu SB, Schickling BM, Vignato JA, Santillan DA, Santillan MK. Selective serotonin reuptake inhibitors and preeclampsia: A quality assessment and meta-analysis. Pregnancy Hypertens. 2022 Dec 1;30:36–43.](https://www.zotero.org/google-docs/?g5hoFp)

[89. Gutierrez PM, Davidson CL, Friese AH, Forster JE. Physical Activity, Suicide Risk Factors, and Suicidal Ideation in a Veteran Sample. Suicide Life Threat Behav. 2016 Jun;46(3):284–92.](https://www.zotero.org/google-docs/?g5hoFp)

[90. Haan E, Westmoreland KE, Schellhas L, Sallis HM, Taylor G, Zuccolo L, et al. Prenatal smoking, alcohol and caffeine exposure and offspring externalizing disorders: a systematic review and meta-analysis. Addiction. 2022;117(10):2602–13.](https://www.zotero.org/google-docs/?g5hoFp)

[91. Haskey C, Galbally M. Mood stabilizers in pregnancy and child developmental outcomes: A systematic review. Aust N Z J Psychiatry. 2017 Nov 1;51(11):1087–97.](https://www.zotero.org/google-docs/?g5hoFp)

[92. Hemels ME, Einarson A, Koren G, Lanctôt KL, Einarson TR. Antidepressant Use during Pregnancy and the Rates of Spontaneous Abortions: A Meta-Analysis. Ann Pharmacother. 2005 May;39(5):803–9.](https://www.zotero.org/google-docs/?g5hoFp)

[93. Hemmati Z, Conti AA, Baldacchino A. Ophthalmic outcomes in children exposed to opioid maintenance treatment in utero: A systematic review and meta-analysis. Neurosci Biobehav Rev. 2022 May 1;136:104601.](https://www.zotero.org/google-docs/?g5hoFp)

[94. Hendrick V, Suri R, Gitlin MJ, Ortiz-Portillo E. Bupropion Use During Pregnancy: A Systematic Review. Prim Care Companion CNS Disord. 2017 Sep 21;19(5):26729.](https://www.zotero.org/google-docs/?g5hoFp)

[95. Hillemacher T, Simen S, Rehme MK, Frieling H. Antipsychotika in der Schwangerschaft: eine systematische Übersichtsarbeit. Nervenarzt. 2021 May 1;92(5):494–500.](https://www.zotero.org/google-docs/?g5hoFp)

[96. Hjorth S, Bromley R, Ystrom E, Lupattelli A, Spigset O, Nordeng H. Use and validity of child neurodevelopment outcome measures in studies on prenatal exposure to psychotropic and analgesic medications – A systematic review. PLOS ONE. 2019 Jul 11;14(7):e0219778.](https://www.zotero.org/google-docs/?g5hoFp)

[97. Huang H, Coleman S, Bridge JA, Yonkers K, Katon W. A meta-analysis of the relationship between antidepressant use in pregnancy and the risk of preterm birth and low birth weight. Gen Hosp Psychiatry. 2014 Jan 1;36(1):13–8.](https://www.zotero.org/google-docs/?g5hoFp)

[98. Hulse GK, Milne E, English DR, Holman CD. The relationship between maternal use of heroin and methadone and infant birth weight. Addict Abingdon Engl. 1997 Nov;92(11):1571–9.](https://www.zotero.org/google-docs/?g5hoFp)

[99. Huntington J, Zantop V. Antidepressant Medications in Pregnancy. Am Fam Physician. 2004 Dec 1;70(11):2195–6.](https://www.zotero.org/google-docs/?g5hoFp)

[100. Iqbal MM, Sobhan T, Ryals T. Effects of Commonly Used Benzodiazepines on the Fetus, the Neonate, and the Nursing Infant. Psychiatr Serv. 2002 Jan;53(1):39–49.](https://www.zotero.org/google-docs/?g5hoFp)

[101. Jain S, Sharma V, Kaushal R. Towards automated real-time detection of misinformation on Twitter. In: 2016 International Conference on Advances in Computing, Communications and Informatics (ICACCI). 2016. p. 2015–20.](https://www.zotero.org/google-docs/?g5hoFp)

[102. Jarde A, Morais M, Kingston D, Giallo R, MacQueen GM, Giglia L, et al. Neonatal Outcomes in Women With Untreated Antenatal Depression Compared With Women Without Depression: A Systematic Review and Meta-analysis. JAMA Psychiatry. 2016 Aug 1;73(8):826–37.](https://www.zotero.org/google-docs/?g5hoFp)

[103. Jentink J, Dolk H, Loane MA, Morris JK, Wellesley D, Garne E, et al. Intrauterine exposure to carbamazepine and specific congenital malformations: systematic review and case-control study. BMJ. 2010 Dec 3;341:c6581.](https://www.zotero.org/google-docs/?g5hoFp)

[104. Jiang H yin, Xu L lian, Li Y cuan, Deng M, Peng C ting, Ruan B. Antidepressant use during pregnancy and risk of postpartum hemorrhage: A systematic review and meta-analysis. J Psychiatr Res. 2016 Dec 1;83:160–7.](https://www.zotero.org/google-docs/?g5hoFp)

[105. Jiang HY, Peng CT, Zhang X, Ruan B. Antidepressant use during pregnancy and the risk of attention-deficit/hyperactivity disorder in the children: a meta-analysis of cohort studies. BJOG Int J Obstet Gynaecol. 2018;125(9):1077–84.](https://www.zotero.org/google-docs/?g5hoFp)

[106. Jiang H yin, Zhang X, Jiang C ming, Fu H bin. Maternal and neonatal outcomes after exposure to ADHD medication during pregnancy: A systematic review and meta-analysis. Pharmacoepidemiol Drug Saf. 2019;28(3):288–95.](https://www.zotero.org/google-docs/?g5hoFp)

[107. Jin F, Qiao C. Association of maternal caffeine intake during pregnancy with low birth weight, childhood overweight, and obesity: a meta-analysis of cohort studies. Int J Obes. 2021 Feb;45(2):279–87.](https://www.zotero.org/google-docs/?g5hoFp)

[108. Jones HE, Finnegan LP, Kaltenbach K. Methadone and Buprenorphine for the Management of Opioid Dependence in Pregnancy. Drugs. 2012 Apr 1;72(6):747–57.](https://www.zotero.org/google-docs/?g5hoFp)

[109. Jones HE, Jansson LM, O’Grady KE, Kaltenbach K. The relationship between maternal methadone dose at delivery and neonatal outcome: Methodological and design considerations. Neurotoxicol Teratol. 2013 Sep 1;39:110–5.](https://www.zotero.org/google-docs/?g5hoFp)

[110. Kang HH, Ahn KH, Hong SC, Kwon BY, Lee EH, Lee JS, et al. Association of citalopram with congenital anomalies: A meta-analysis. Obstet Gynecol Sci. 2017 Mar 16;60(2):145–53.](https://www.zotero.org/google-docs/?g5hoFp)

[111. Kaplan YC, Keskin-Arslan E, Acar S, Sozmen K. Prenatal selective serotonin reuptake inhibitor use and the risk of autism spectrum disorder in children: A systematic review and meta-analysis. Reprod Toxicol. 2016 Dec 1;66:31–43.](https://www.zotero.org/google-docs/?g5hoFp)

[112. Kautzky A, Slamanig R, Unger A, Höflich A. Neonatal outcome and adaption after in utero exposure to antidepressants: A systematic review and meta-analysis. Acta Psychiatr Scand. 2022 Jan;145(1):6–28.](https://www.zotero.org/google-docs/?g5hoFp)

[113. Kinsella M, Halliday LOE, Shaw M, Capel Y, Nelson SM, Kearns RJ. Buprenorphine Compared with Methadone in Pregnancy: A Systematic Review and Meta-Analysis. Subst Use Misuse. 2022 Jul 29;57(9):1400–16.](https://www.zotero.org/google-docs/?g5hoFp)

[114. Kiplagat S, Coudray MS, Taskin T, Dawit R, Gbadamosi S, Madhivanan P. Methodological Evaluation of Antipsychotic Use During Pregnancy and Gestational Diabetes Mellitus. J Clin Psychopharmacol. 2020 Jun;40(3):319.](https://www.zotero.org/google-docs/?g5hoFp)

[115. Knight R, Wittkowski A, Bromley RL. Neurodevelopmental outcomes in children exposed to newer antiseizure medications: A systematic review. Epilepsia. 2021;62(8):1765–79.](https://www.zotero.org/google-docs/?g5hoFp)

[116. Kobayashi T, Matsuyama T, Takeuchi M, Ito S. Autism spectrum disorder and prenatal exposure to selective serotonin reuptake inhibitors: A systematic review and meta-analysis. Reprod Toxicol. 2016 Oct 1;65:170–8.](https://www.zotero.org/google-docs/?g5hoFp)

[117. Kong L, Zhou T, Wang B, Gao Z, Wang C. The risks associated with the use of lamotrigine during pregnancy. Int J Psychiatry Clin Pract. 2018 Jan 2;22(1):2–5.](https://www.zotero.org/google-docs/?g5hoFp)

[118. Kucukgoncu S, Guloksuz S, Celik K, Bahtiyar MO, Luykx JJ, Rutten BPF, et al. Antipsychotic Exposure in Pregnancy and the Risk of Gestational Diabetes: A Systematic Review and Meta-analysis. Schizophr Bull. 2020 Feb 26;46(2):311–8.](https://www.zotero.org/google-docs/?g5hoFp)

[119. Kuller JA, Katz VL, McMahon MJ, Wells SR, Bashford RA. Pharmacologic treatment of psychiatric disease in pregnancy and lactation: Fetal and neonatal effects. Obstet Gynecol. 1996 May 1;87(5, Part 1):789–94.](https://www.zotero.org/google-docs/?g5hoFp)

[120. Lassen D, Ennis ZN, Damkier P. First-Trimester Pregnancy Exposure to Venlafaxine or Duloxetine and Risk of Major Congenital Malformations: A Systematic Review. Basic Clin Pharmacol Toxicol. 2016;118(1):32–6.](https://www.zotero.org/google-docs/?g5hoFp)

[121. Lattimore KA, Donn SM, Kaciroti N, Kemper AR, Neal CR, Vazquez DM. Selective Serotonin Reuptake Inhibitor (SSRI) Use during Pregnancy and Effects on the Fetus and Newborn: A Meta-Analysis. J Perinatol. 2005 Sep 1;25(9):595–604.](https://www.zotero.org/google-docs/?g5hoFp)

[122. Lee SJ, Bora S, Austin NC, Westerman A, Henderson JMT. Neurodevelopmental Outcomes of Children Born to Opioid-Dependent Mothers: A Systematic Review and Meta-Analysis. Acad Pediatr. 2020 Apr 1;20(3):308–18.](https://www.zotero.org/google-docs/?g5hoFp)

[123. Leshem R, Bar-Oz B, Diav-Citrin O, Gbaly S, Soliman J, Renoux C, et al. Selective Serotonin Reuptake Inhibitors (SSRIs) and Serotonin Norepinephrine Reuptake Inhibitors (SNRIs) During Pregnancy and the Risk for Autism spectrum disorder (ASD) and Attention deficit hyperactivity disorder (ADHD) in the Offspring: A True Effect or a Bias? A Systematic Review & Meta-Analysis. Curr Neuropharmacol. 19(6):896–906.](https://www.zotero.org/google-docs/?g5hoFp)

[124. Leung MTY, Wong KH, Ho PWH, Ip P, Wei L, Wong ICK, et al. Gestational exposure to antidepressants and risk of seizure in offspring: A systematic review and meta-analysis. Neurosci Biobehav Rev. 2021 Dec 1;131:345–59.](https://www.zotero.org/google-docs/?g5hoFp)

[125. Li J, Zhao H, Song JM, Zhang J, Tang YL, Xin CM. A meta-analysis of risk of pregnancy loss and caffeine and coffee consumption during pregnancy. Int J Gynecol Obstet. 2015;130(2):116–22.](https://www.zotero.org/google-docs/?g5hoFp)

[126. Lind JN, Interrante JD, Ailes EC, Gilboa SM, Khan S, Frey MT, et al. Maternal Use of Opioids During Pregnancy and Congenital Malformations: A Systematic Review. Pediatrics. 2017 Jun 1;139(6):e20164131.](https://www.zotero.org/google-docs/?g5hoFp)

[127. Link HM, Jones H, Miller L, Kaltenbach K, Seligman N. Buprenorphine-naloxone use in pregnancy: a systematic review and metaanalysis. Am J Obstet Gynecol MFM. 2020 Aug 1;2(3):100179.](https://www.zotero.org/google-docs/?g5hoFp)

[128. Liu ZF, Sylivris A, Gordon M, Sundram S. The association between tryptophan levels and postpartum mood disorders: a systematic review and meta-analysis. BMC Psychiatry. 2022 Aug 8;22(1):539.](https://www.zotero.org/google-docs/?g5hoFp)

[129. Longo B, Forinash AB, Murphy JA. Drug Information Rounds Levetiracetam Use in Pregnancy. Ann Pharmacother. 2009 Oct 1;43(10):1692–5.](https://www.zotero.org/google-docs/?g5hoFp)

[130. Lopez-Yarto M, Ruiz-Mirazo E, Holloway AC, Taylor VH, McDonald SD. Do psychiatric medications, especially antidepressants, adversely impact maternal metabolic outcomes? J Affect Disord. 2012 Dec 10;141(2):120–9.](https://www.zotero.org/google-docs/?g5hoFp)

[131. Lorenzo L, Einarson A. Antidepressant use in pregnancy: an evaluation of adverse outcomes excluding malformation. Isr J Psychiatry Relat Sci. 2014;51(2):94–104.](https://www.zotero.org/google-docs/?g5hoFp)

[132. Malm H. Prenatal Exposure to Selective Serotonin Reuptake Inhibitors and Infant Outcome. Ther Drug Monit. 2012 Dec;34(6):607.](https://www.zotero.org/google-docs/?g5hoFp)

[133. Man KKC, Tong HHY, Wong LYL, Chan EW, Simonoff E, Wong ICK. Exposure to selective serotonin reuptake inhibitors during pregnancy and risk of autism spectrum disorder in children: A systematic review and meta-analysis of observational studies. Neurosci Biobehav Rev. 2015 Feb 1;49:82–9.](https://www.zotero.org/google-docs/?g5hoFp)

[134. Man KKC, Chan EW, Ip P, Coghill D, Simonoff E, Chan PKL, et al. Prenatal antidepressant exposure and the risk of attention-deficit hyperactivity disorder in children: A systematic review and meta-analysis. Neurosci Biobehav Rev. 2018 Mar 1;86:1–11.](https://www.zotero.org/google-docs/?g5hoFp)

[135. Masarwa R, Bar-Oz B, Gorelik E, Reif S, Perlman A, Matok I. Prenatal exposure to selective serotonin reuptake inhibitors and serotonin norepinephrine reuptake inhibitors and risk for persistent pulmonary hypertension of the newborn: a systematic review, meta-analysis, and network meta-analysis. Am J Obstet Gynecol. 2019 Jan 1;220(1):57.e1-57.e13.](https://www.zotero.org/google-docs/?g5hoFp)

[136. Maslova E, Bhattacharya S, Lin SW, Michels KB. Caffeine consumption during pregnancy and risk of preterm birth: a meta-analysis1234. Am J Clin Nutr. 2010 Nov 1;92(5):1120–32.](https://www.zotero.org/google-docs/?g5hoFp)

[137. Matalon S, Schechtman S, Goldzweig G, Ornoy A. The teratogenic effect of carbamazepine: a meta-analysis of 1255 exposures. Reprod Toxicol. 2002 Jan;16(1):9–17.](https://www.zotero.org/google-docs/?g5hoFp)

[138. Mathew S, Bichenapally S, Khachatryan V, Muazzam A, Hamal C, Velugoti LSDR, et al. Role of Serotoninergic Antidepressants in the Development of Autism Spectrum Disorders: A Systematic Review. Cureus [Internet]. 2022 Aug 28 [cited 2024 Jan 4];14(8). Available from: https://www.cureus.com/articles/108354-role-of-serotoninergic-antidepressants-in-the-development-of-autism-spectrum-disorders-a-systematic-review](https://www.zotero.org/google-docs/?g5hoFp)

[139. Mccauley-Elsom K, Gurvich C, Elsom SJ, Kulkarni J. Antipsychotics in pregnancy. J Psychiatr Ment Health Nurs. 2010;17(2):97–104.](https://www.zotero.org/google-docs/?g5hoFp)

[140. McDonagh MS, Matthews A, Phillipi C, Romm J, Peterson K, Thakurta S, et al. Depression Drug Treatment Outcomes in Pregnancy and the Postpartum Period: A Systematic Review and Meta-analysis. Obstet Gynecol. 2014 Sep;124(3):526.](https://www.zotero.org/google-docs/?g5hoFp)

[141. Medveczky E, Puhó E, Czeizel EA. The use of drugs in mothers of offspring with neural-tube defects. Pharmacoepidemiol Drug Saf. 2004;13(7):443–55.](https://www.zotero.org/google-docs/?g5hoFp)

[142. Mehta TM, Van Lieshout RJ. A review of the safety of clozapine during pregnancy and lactation. Arch Womens Ment Health. 2017 Feb 1;20(1):1–9.](https://www.zotero.org/google-docs/?g5hoFp)

[143. Mezzacappa A, Lasica PA, Gianfagna F, Cazas O, Hardy P, Falissard B, et al. Risk for Autism Spectrum Disorders According to Period of Prenatal Antidepressant Exposure: A Systematic Review and Meta-analysis. JAMA Pediatr. 2017 Jun 1;171(6):555–63.](https://www.zotero.org/google-docs/?g5hoFp)

[144. Misri S, Burgmann A, Kostaras D. Are SSRIs safe for pregnant and breastfeeding women? Can Fam Physician Med Fam Can. 2000 Mar;46:626–8, 631–3.](https://www.zotero.org/google-docs/?g5hoFp)

[145. Mitchell J, Goodman J. Comparative effects of antidepressant medications and untreated major depression on pregnancy outcomes: a systematic review. Arch Womens Ment Health. 2018 Oct 1;21(5):505–16.](https://www.zotero.org/google-docs/?g5hoFp)

[146. Molenaar NM, Bais B, Lambregtse-van den Berg MP, Mulder CL, Howell EA, Fox NS, et al. The international prevalence of antidepressant use before, during, and after pregnancy: A systematic review and meta-analysis of timing, type of prescriptions and geographical variability. J Affect Disord. 2020 Mar 1;264:82–9.](https://www.zotero.org/google-docs/?g5hoFp)

[147. Monnelly VJ, Hamilton R, Chappell FM, Mactier H, Boardman JP. Childhood neurodevelopment after prescription of maintenance methadone for opioid dependency in pregnancy: a systematic review and meta-analysis. Dev Med Child Neurol. 2019;61(7):750–60.](https://www.zotero.org/google-docs/?g5hoFp)

[148. Montouris G. Safety of the newer antiepileptic drug oxcarbazepine during pregnancy. Curr Med Res Opin. 2005 May 1;21(5):693–701.](https://www.zotero.org/google-docs/?g5hoFp)

[149. Munk-Olsen T, Liu X, Viktorin A, Brown HK, Di Florio A, D’Onofrio BM, et al. Maternal and infant outcomes associated with lithium use in pregnancy: an international collaborative meta-analysis of six cohort studies. Lancet Psychiatry. 2018 Aug 1;5(8):644–52.](https://www.zotero.org/google-docs/?g5hoFp)

[150. Myles N, Newall H, Ward H, Large M. Systematic meta-analysis of individual selective serotonin reuptake inhibitor medications and congenital malformations. Aust N Z J Psychiatry. 2013 Nov 1;47(11):1002–12.](https://www.zotero.org/google-docs/?g5hoFp)

[151. Myung SK, Ju W, Jung HS, Park CH, Oh SW, Seo H, et al. Efficacy and safety of pharmacotherapy for smoking cessation among pregnant smokers: a meta-analysis. BJOG Int J Obstet Gynaecol. 2012;119(9):1029–39.](https://www.zotero.org/google-docs/?g5hoFp)

[152. Nelson LF, Yocum VK, Patel KD, Qeadan F, Hsi A, Weitzen S. Cognitive Outcomes of Young Children After Prenatal Exposure to Medications for Opioid Use Disorder: A Systematic Review and Meta-analysis. JAMA Netw Open. 2020 Mar 18;3(3):e201195.](https://www.zotero.org/google-docs/?g5hoFp)

[153. Newmark RL, Bogen DL, Wisner KL, Isaac M, Ciolino JD, Clark CT. Risk-Benefit assessment of infant exposure to lithium through breast milk: a systematic review of the literature. Int Rev Psychiatry. 2019 Apr 3;31(3):295–304.](https://www.zotero.org/google-docs/?g5hoFp)

[154. Nikfar S, Rahimi R, Hendoiee N, Abdollahi M. Increasing the risk of spontaneous abortion and major malformations in newborns following use of serotonin reuptake inhibitors during pregnancy: A systematic review and updated meta-analysis. DARU J Pharm Sci. 2012 Nov 1;20(1):75.](https://www.zotero.org/google-docs/?g5hoFp)

[155. Noormohammadi A, Forinash A, Yancey A, Crannage E, Campbell K, Shyken J. Buprenorphine Versus Methadone for Opioid Dependence in Pregnancy. Ann Pharmacother. 2016 Aug 1;50(8):666–72.](https://www.zotero.org/google-docs/?g5hoFp)

[156. Ordean A, Tubman-Broeren M. Safety and Efficacy of Buprenorphine-Naloxone in Pregnancy: A Systematic Review of the Literature. Pathophysiology. 2023 Mar;30(1):27–36.](https://www.zotero.org/google-docs/?g5hoFp)

[157. Orsolini L, Bellantuono C. Serotonin reuptake inhibitors and breastfeeding: a systematic review. Hum Psychopharmacol Clin Exp. 2015;30(1):4–20.](https://www.zotero.org/google-docs/?g5hoFp)

[158. Orsolini L, Sceusa F, Pompili S, Mauro A, Salvi V, Volpe U. Severe and persistent mental illness (SPMI) in pregnancy and breastfeeding: focus on second-generation long acting injectable antipsychotics. Expert Opin Drug Saf. 2021 Oct 3;20(10):1207–24.](https://www.zotero.org/google-docs/?g5hoFp)

[159. Pacchiarotti I, León-Caballero J, Murru A, Verdolini N, Furio MA, Pancheri C, et al. Mood stabilizers and antipsychotics during breastfeeding: Focus on bipolar disorder. Eur Neuropsychopharmacol. 2016 Oct 1;26(10):1562–78.](https://www.zotero.org/google-docs/?g5hoFp)

[160. Painuly N, Painuly R, Heun R, Sharan P. Risk of cardiovascular malformations after exposure to paroxetine in pregnancy: meta-analysis. The Psychiatrist. 2013 Jun;37(6):198–203.](https://www.zotero.org/google-docs/?g5hoFp)

[161. Pariente G, Leibson T, Shulman T, Adams-Webber T, Barzilay E, Nulman I. Pregnancy Outcomes Following In Utero Exposure to Lamotrigine: A Systematic Review and Meta-Analysis. CNS Drugs. 2017 Jun 1;31(6):439–50.](https://www.zotero.org/google-docs/?g5hoFp)

[162. Pedersen LH. Meta-analysis: antidepressant exposure during pregnancy is associated with poor neonatal adaptation. BMJ Evid-Based Med. 2014 Apr 1;19(2):76–76.](https://www.zotero.org/google-docs/?g5hoFp)

[163. Petersen I, McCrea RL, Sammon CJ, Osborn DP, Evans SJ, Cowen PJ, et al. Risks and benefits of psychotropic medication in pregnancy: cohort studies based on UK electronic primary care health records. Health Technol Assess. 2016 Apr 1;20(23):1–176.](https://www.zotero.org/google-docs/?g5hoFp)

[164. Pinheiro E, Bogen DL, Hoxha D, Ciolino JD, Wisner KL. Sertraline and breastfeeding: review and meta-analysis. Arch Womens Ment Health. 2015 Apr 1;18(2):139–46.](https://www.zotero.org/google-docs/?g5hoFp)

[165. Poels EMP, Bijma HH, Galbally M, Bergink V. Lithium during pregnancy and after delivery: a review. Int J Bipolar Disord. 2018 Dec 2;6(1):26.](https://www.zotero.org/google-docs/?g5hoFp)

[166. Prady SL, Hanlon I, Fraser LK, Mikocka-Walus A. A systematic review of maternal antidepressant use in pregnancy and short- and long-term offspring’s outcomes. Arch Womens Ment Health. 2018 Apr 1;21(2):127–40.](https://www.zotero.org/google-docs/?g5hoFp)

[167. Previti G, Pawlby S, Chowdhury S, Aguglia E, Pariante CM. Neurodevelopmental outcome for offspring of women treated for antenatal depression: a systematic review. Arch Womens Ment Health. 2014 Dec 1;17(6):471–83.](https://www.zotero.org/google-docs/?g5hoFp)

[168. Rahimi R, Nikfar S, Abdollahi M. Pregnancy outcomes following exposure to serotonin reuptake inhibitors: a meta-analysis of clinical trials. Reprod Toxicol. 2006 Nov;22(4):571–5.](https://www.zotero.org/google-docs/?g5hoFp)

[169. Rais TB, Rais A. Association Between Antidepressants Use During Pregnancy and Autistic Spectrum Disorders: A Meta-analysis. Innov Clin Neurosci. 2014 May;11(5–6):18–22.](https://www.zotero.org/google-docs/?g5hoFp)

[170. Rausgaard NLK, Ibsen IO, Jørgensen JS, Lamont RF, Ravn P. Management and monitoring of opioid use in pregnancy. Acta Obstet Gynecol Scand. 2020 Jan;99(1):7–15.](https://www.zotero.org/google-docs/?g5hoFp)

[171. Reising VA, Bergren MD, Bennett A. Care and Treatment Recommendations for Pregnant Women with Opioid Use Disorder. MCN Am J Matern Nurs. 2019 Aug;44(4):212.](https://www.zotero.org/google-docs/?g5hoFp)

[172. Rhee J, Kim R, Kim Y, Tam M, Lai Y, Keum N, et al. Maternal Caffeine Consumption during Pregnancy and Risk of Low Birth Weight: A Dose-Response Meta-Analysis of Observational Studies. PLOS ONE. 2015 Jul 20;10(7):e0132334.](https://www.zotero.org/google-docs/?g5hoFp)

[173. Ricks N, Comer L, Liu F, DeGrande H, Adeniran O. Substance Use and Preconception Care: A Review of the Literature. Int J Womens Health Reprod Sci. 2017 Jan 1;5(1):3–10.](https://www.zotero.org/google-docs/?g5hoFp)

[174. Riggin L, Frankel Z, Moretti M, Pupco A, Koren G. MOTHERISK ROUNDS: The Fetal Safety of Fluoxetine: A Systematic Review and Meta-Analysis. J Obstet Gynaecol Can. 2013 Apr 1;35(4):362–9.](https://www.zotero.org/google-docs/?g5hoFp)

[175. Rubinchik SM, Kablinger AS, Gardner JS. Medications for panic disorder and generalized anxiety disorder during pregnancy. Prim Care Companion J Clin Psychiatry. 2005;7(3):100–5.](https://www.zotero.org/google-docs/?g5hoFp)

[176. Ruisch IH, Dietrich A, Glennon JC, Buitelaar JK, Hoekstra PJ. Maternal substance use during pregnancy and offspring conduct problems: A meta-analysis. Neurosci Biobehav Rev. 2018 Jan 1;84:325–36.](https://www.zotero.org/google-docs/?g5hoFp)

[177. Ryan KS, Prewitt KC, Hayer S, Hedges MA, Benson AE, Lo JO. Opioid Use in Pregnancy: A Review. Obstet Gynecol Surv. 2023 Jan;78(1):35.](https://www.zotero.org/google-docs/?g5hoFp)

[178. Santone G, Ricchi G, Rocchetti D, Tofani S, Bellantuono C. Is the exposure to antidepressant drugs in early pregnancy a risk factor for spontaneous abortion? A review of available evidences. Epidemiol Psychiatr Sci. 2009 Sep;18(3):240–7.](https://www.zotero.org/google-docs/?g5hoFp)

[179. Schmidt CT, Deligiannidis KM, Kittel-Schneider S, Frodl T, Spigset O, Paulzen M, et al. Transfer of anticonvulsants and lithium into amniotic fluid, umbilical cord blood & breast milk: A systematic review & combined analysis. Prog Neuropsychopharmacol Biol Psychiatry. 2023 Jun 8;124:110733.](https://www.zotero.org/google-docs/?g5hoFp)

[180. Schoretsanitis G, Spigset O, Stingl JC, Deligiannidis KM, Paulzen M, Westin AA. The impact of pregnancy on the pharmacokinetics of antidepressants: a systematic critical review and meta-analysis. Expert Opin Drug Metab Toxicol. 2020 May 3;16(5):431–40.](https://www.zotero.org/google-docs/?g5hoFp)

[181. Scialli AR. Paroxetine exposure during pregnancy and cardiac malformations. Birt Defects Res A Clin Mol Teratol. 2010;88(3):175–7.](https://www.zotero.org/google-docs/?g5hoFp)

[182. Seeman MV. Clinical interventions for women with schizophrenia: pregnancy. Acta Psychiatr Scand. 2013;127(1):12–22.](https://www.zotero.org/google-docs/?g5hoFp)

[183. Sharma V, MBBS, Pope CJ. Pregnancy and Bipolar Disorder: A Systematic Review. J Clin Psychiatry. 2012 Aug 21;73(11):15206.](https://www.zotero.org/google-docs/?g5hoFp)

[184. Shen ZQ, Gao SY, Li SX, Zhang TN, Liu CX, Lv HC, et al. Sertraline use in the first trimester and risk of congenital anomalies: a systemic review and meta-analysis of cohort studies. Br J Clin Pharmacol. 2017;83(4):909–22.](https://www.zotero.org/google-docs/?g5hoFp)

[185. Simoncelli M, Martin BZ, Berard A. Antidepressant Use During Pregnancy: A Critical Systematic Review of the Literature. Curr Drug Saf. 2010 Apr 1;5(2):153–70.](https://www.zotero.org/google-docs/?g5hoFp)

[186. Suri R, Lin AS, Cohen LS, Altshuler LL. Acute and Long-Term Behavioral Outcome of Infants and Children Exposed in Utero to Either Maternal Depression or Antidepressants: A Review of the Literature. J Clin Psychiatry. 2014 Oct 28;75(10):371.](https://www.zotero.org/google-docs/?g5hoFp)

[187. Tak CR, Job KM, Schoen-Gentry K, Campbell SC, Carroll P, Costantine M, et al. The impact of exposure to antidepressant medications during pregnancy on neonatal outcomes: a review of retrospective database cohort studies. Eur J Clin Pharmacol. 2017 Sep 1;73(9):1055–69.](https://www.zotero.org/google-docs/?g5hoFp)

[188. Tanoshima M, Kobayashi T, Tanoshima R, Beyene J, Koren G, Ito S. Risks of congenital malformations in offspring exposed to valproic acid in utero: A systematic review and cumulative meta-analysis. Clin Pharmacol Ther. 2015;98(4):417–41.](https://www.zotero.org/google-docs/?g5hoFp)

[189. Taylor L, Claire R, Campbell K, Coleman-Haynes T, Leonardi-Bee J, Chamberlain C, et al. Fetal safety of nicotine replacement therapy in pregnancy: systematic review and meta-analysis. Addiction. 2021;116(2):239–77.](https://www.zotero.org/google-docs/?g5hoFp)

[190. Terrana N, Koren G, Pivovarov J, Etwel F, Nulman I. Pregnancy Outcomes Following In Utero Exposure to Second-Generation Antipsychotics: A Systematic Review and Meta-Analysis. J Clin Psychopharmacol. 2015 Oct;35(5):559.](https://www.zotero.org/google-docs/?g5hoFp)

[191. Thanigaivel R, Bretag-Norris R, Amos A, McDermott B. A systematic review of maternal and infant outcomes after clozapine continuation in pregnancy. Int J Psychiatry Clin Pract. 2022 Jun 1;26(2):178–82.](https://www.zotero.org/google-docs/?g5hoFp)

[192. Thormahlen GM. Paroxetine Use During Pregnancy: Is it Safe? Ann Pharmacother. 2006 Oct 1;40(10):1834–7.](https://www.zotero.org/google-docs/?g5hoFp)

[193. Tillery EE, Tonet RL, Trahan CA. Oh baby! A review of mood stabilizers for bipolar disorder in the child-bearing woman. Ment Health Clin. 2013 Aug 1;3(2):61–70.](https://www.zotero.org/google-docs/?g5hoFp)

[194. Tosato S, Albert U, Tomassi S, Iasevoli F, Carmassi C, Ferrari S, et al. A Systematized Review of Atypical Antipsychotics in Pregnant Women: Balancing Between Risks of Untreated Illness and Risks of Drug-Related Adverse Effects. J Clin Psychiatry. 2017 May 24;78(5):1643.](https://www.zotero.org/google-docs/?g5hoFp)

[195. Towers CV, Terry P, Rackley B, Hennessy M, Visconti K. Fetal Outcomes with Detoxification from Opioid Drugs during Pregnancy: A Systematic Review. Am J Perinatol. 2020 Jun;37(07):679–88.](https://www.zotero.org/google-docs/?g5hoFp)

[196. Tuccori M, Testi A, Antonioli L, Fornai M, Montagnani S, Ghisu N, et al. Safety concerns associated with the use of serotonin reuptake inhibitors and other serotonergic/noradrenergic antidepressants during pregnancy: A review. Clin Ther. 2009 Jan 1;31:1426–53.](https://www.zotero.org/google-docs/?g5hoFp)

[197. Tuccori M, Montagnani S, Testi A, Ruggiero E, Mantarro S, Scollo C, et al. Use of Selective Serotonin Reuptake Inhibitors during Pregnancy and Risk of Major and Cardiovascular Malformations: An Update. Postgrad Med. 2010 Jul 1;122(4):49–65.](https://www.zotero.org/google-docs/?g5hoFp)

[198. Turner E, Jones M, Vaz LR, Coleman T. Systematic Review and Meta-Analysis to Assess the Safety of Bupropion and Varenicline in Pregnancy. Nicotine Tob Res. 2019 Jul 17;21(8):1001–10.](https://www.zotero.org/google-docs/?g5hoFp)

[199. Udechuku A, Nguyen T, Hill R, Szego K. Antidepressants in pregnancy: a systematic review. Aust N Z J Psychiatry. 2010 Nov;44(11):978–96.](https://www.zotero.org/google-docs/?g5hoFp)

[200. Uguz F, Sharma V. Mood stabilizers during breastfeeding: a systematic review of the recent literature. Bipolar Disord. 2016;18(4):325–33.](https://www.zotero.org/google-docs/?g5hoFp)

[201. Uguz F. Is There Any Association Between Use of Antidepressants and Preeclampsia or Gestational Hypertension?: A Systematic Review of Current Studies. J Clin Psychopharmacol. 2017 Feb;37(1):72.](https://www.zotero.org/google-docs/?g5hoFp)

[202. Uguz F. Maternal Antidepressant Use During Pregnancy and the Risk of Attention-Deficit/Hyperactivity Disorder in Children: A Systematic Review of the Current Literature. J Clin Psychopharmacol. 2018 Jun;38(3):254.](https://www.zotero.org/google-docs/?g5hoFp)

[203. Uguz F. The Use of Antidepressant Medications During Pregnancy and the Risk of Neonatal Seizures: A Systematic Review. J Clin Psychopharmacol. 2019 Oct;39(5):479.](https://www.zotero.org/google-docs/?g5hoFp)

[204. Uguz F. Antipsychotic Use During Pregnancy and the Risk of Gestational Diabetes Mellitus: A Systematic Review. J Clin Psychopharmacol. 2019 Apr;39(2):162.](https://www.zotero.org/google-docs/?g5hoFp)

[205. Uguz F. Selective serotonin reuptake inhibitors and the risk of congenital anomalies: a systematic review of current meta-analyses. Expert Opin Drug Saf. 2020 Dec 1;19(12):1595–604.](https://www.zotero.org/google-docs/?g5hoFp)

[206. Uguz F. Pharmacological prevention of mood episodes in women with bipolar disorder during the perinatal period: A systematic review of current literature. Asian J Psychiatry. 2020 Aug 1;52:102145.](https://www.zotero.org/google-docs/?g5hoFp)

[207. Uguz F. Neonatal and Childhood Outcomes in Offspring of Pregnant Women Using Antidepressant Medications: A Critical Review of Current Meta-Analyses. J Clin Pharmacol. 2021;61(2):146–58.](https://www.zotero.org/google-docs/?g5hoFp)

[208. Uguz F. The Relationship Between Maternal Antidepressants and Neonatal Hypoglycemia: A Systematic Review. Alpha Psychiatry. 2021 Sep;22(5):224–9.](https://www.zotero.org/google-docs/?g5hoFp)

[209. Urato AC. Antidepressant exposure in utero is associated with an increased risk of cardiovascular malformation. BMJ Evid-Based Med. 2014 Apr 1;19(2):72–72.](https://www.zotero.org/google-docs/?g5hoFp)

[210. van Driel JJ, Wennink MB. Intra-uteriene blootstelling aan SSRI’s: geen reden voor routinematige controle op hypoglykemie. NED TIJDSCHR GENEESKD.](https://www.zotero.org/google-docs/?g5hoFp)

[211. Viswanathan M, Middleton JC, Stuebe AM, Berkman ND, Goulding AN, McLaurin‐Jiang S, et al. Maternal, Fetal, and Child Outcomes of Mental Health Treatments in Women: A Meta‐Analysis of Pharmacotherapy. Psychiatr Res Clin Pract. 2021 Sep;3(3):123–40.](https://www.zotero.org/google-docs/?g5hoFp)

[212. Vitale SG, Laganà AS, Muscatello MRA, La Rosa VL, Currò V, Pandolfo G, et al. Psychopharmacotherapy in Pregnancy and Breastfeeding. Obstet Gynecol Surv. 2016 Dec;72(12):721.](https://www.zotero.org/google-docs/?g5hoFp)

[213. Viuff ACF, Pedersen LH, Kyng K, Staunstrup NH, Børglum A, Henriksen TB. Antidepressant medication during pregnancy and epigenetic changes in umbilical cord blood: a systematic review. Clin Epigenetics. 2016 Sep 7;8(1):94.](https://www.zotero.org/google-docs/?g5hoFp)

[214. Vlenterie R, van Gelder MMHJ, Anderson HR, Andersson L, Broekman BFP, Dubnov-Raz G, et al. Associations Between Maternal Depression, Antidepressant Use During Pregnancy, and Adverse Pregnancy Outcomes: An Individual Participant Data Meta-analysis. Obstet Gynecol. 2021 Oct;138(4):633.](https://www.zotero.org/google-docs/?g5hoFp)

[215. Wang S, Yang L, Wang L, Gao L, Xu B, Xiong Y. Selective Serotonin Reuptake Inhibitors (SSRIs) and the Risk of Congenital Heart Defects: A Meta‐Analysis of Prospective Cohort Studies. J Am Heart Assoc. 2015 May 19;4(5):e001681.](https://www.zotero.org/google-docs/?g5hoFp)

[216. Wang J, Cosci F. Neonatal Withdrawal Syndrome following Late in utero Exposure to Selective Serotonin Reuptake Inhibitors: A Systematic Review and Meta-Analysis of Observational Studies. Psychother Psychosom. 2021 May 10;90(5):299–307.](https://www.zotero.org/google-docs/?g5hoFp)

[217. Wang Z, Wong ICK, Man KKC, Alfageh BH, Mongkhon P, Brauer R. The use of antipsychotic agents during pregnancy and the risk of gestational diabetes mellitus: a systematic review and meta-analysis. Psychol Med. 2021 Apr;51(6):1028–37.](https://www.zotero.org/google-docs/?g5hoFp)

[218. Wang Z, Man KKC, Ma T, Howard LM, Wei L, Wong ICK, et al. Association between antipsychotic use in pregnancy and the risk of gestational diabetes: Population-based cohort studies from the United Kingdom and Hong Kong and an updated meta-analysis. Schizophr Res. 2021 Mar 1;229:55–62.](https://www.zotero.org/google-docs/?g5hoFp)

[219. Wang Z, Brauer R, Man KKC, Alfageh B, Mongkhon P, Wong ICK. Prenatal exposure to antipsychotic agents and the risk of congenital malformations in children: A systematic review and meta-analysis. Br J Clin Pharmacol. 2021;87(11):4101–23.](https://www.zotero.org/google-docs/?g5hoFp)

[220. Wang X, Wang Y, Tang B, Feng X. Opioid exposure during pregnancy and the risk of congenital malformation: a meta-analysis of cohort studies. BMC Pregnancy Childbirth. 2022 May 11;22(1):401.](https://www.zotero.org/google-docs/?g5hoFp)

[221. Wen SW, Walker M. The Use of Selective Serotonin Reuptake Inhibitors in Pregnancy. J Obstet Gynaecol Can. 2004 Sep 1;26(9):819–22.](https://www.zotero.org/google-docs/?g5hoFp)

[222. Wen SW, Walker M. Risk of Fetal Exposure to Tricyclic Antidepressants. J Obstet Gynaecol Can. 2004 Oct;26(10):887–92.](https://www.zotero.org/google-docs/?g5hoFp)

[223. Wesseloo R, Bergink V. Maternale en neonatale uitkomsten na lithiumgebruik tijdens de zwangerschap: Internationale meta-analyse. Tijdschr Voor Psychiatr [Internet]. 2019 Feb [cited 2024 Jan 4];61(2). Available from: https://scholars.mssm.edu/en/publications/maternale-en-neonatale-uitkomsten-na-lithiumgebruik-tijdens-de-zw](https://www.zotero.org/google-docs/?g5hoFp)

[224. Wisner KL. Pharmacologic Treatment of Depression During Pregnancy. JAMA. 1999 Oct 6;282(13):1264.](https://www.zotero.org/google-docs/?g5hoFp)

[225. Wurst KE, Poole C, Ephross SA, Olshan AF. First trimester paroxetine use and the prevalence of congenital, specifically cardiac, defects: A meta-analysis of epidemiological studies. Birt Defects Res A Clin Mol Teratol. 2010;88(3):159–70.](https://www.zotero.org/google-docs/?g5hoFp)

[226. Yamaguchi ET, Cardoso MMSC, Torres MLA, Andrade AG de. Drogas de abuso e gravidez. Arch Clin Psychiatry São Paulo. 2008;35:44–7.](https://www.zotero.org/google-docs/?g5hoFp)

[227. Zedler BK, Mann AL, Kim MM, Amick HR, Joyce AR, Murrelle EL, et al. Buprenorphine compared with methadone to treat pregnant women with opioid use disorder: a systematic review and meta-analysis of safety in the mother, fetus and child. Addiction. 2016;111(12):2115–28.](https://www.zotero.org/google-docs/?g5hoFp)

[228. Zhang TN, Gao SY, Shen ZQ, Li D, Liu CX, Lv HC, et al. Use of selective serotonin-reuptake inhibitors in the first trimester and risk of cardiovascular-related malformations: a meta-analysis of cohort studies. Sci Rep. 2017 Feb 21;7(1):43085.](https://www.zotero.org/google-docs/?g5hoFp)

[229. Zhao L, Cheng C, Bouchard L. Medication-Assisted Treatment for Opioid Use Disorder in Pregnancy: Practical Applications and Clinical Impact. Obstet Gynecol Surv. 2020 Mar;75(3):175.](https://www.zotero.org/google-docs/?g5hoFp)

[230. Zheng L, Yang H, Dallmann A. Antidepressants and Antipsychotics in Human Pregnancy: Transfer Across the Placenta and Opportunities for Modeling Studies. J Clin Pharmacol. 2022;62(S1):S115–28.](https://www.zotero.org/google-docs/?g5hoFp)

[231. Zusman EZ, Lavu A, Pawliuk C, Pawluski J, Hutchison SM, Platt RW, et al. Associations Between Prenatal Exposure to Serotonergic Medications and Biobehavioral Stress Regulation: Protocol for a Systematic Review and Meta-analysis. JMIR Res Protoc. 2022 Mar 28;11(3):e33363.](https://www.zotero.org/google-docs/?g5hoFp)

[232. Zwink N, Jenetzky E. Maternal drug use and the risk of anorectal malformations: systematic review and meta-analysis. Orphanet J Rare Dis. 2018 May 10;13(1):75.](https://www.zotero.org/google-docs/?g5hoFp)
