# Supplementary material for: Safety of psychotropic medications in pregnancy: an umbrella review
Source: Mol Psychiatry. 2024 Sep 12;30(1):327–35. doi: 10.1038/s41380-024-02697-0 (PMC11649568; doi:10.1038/s41380-024-02697-0)
Supplement: Supplementary file 3 — Supplementary material 3 [file 41380_2024_2697_MOESM3_ESM.docx]

**Supplementary material 3**

**Safety of psychotropic medications in pregnancy: an umbrella review**

Nicholas Fabiano MD^1^, Stanley Wong MD^1,2^, Arnav Gupta MD^3,4^, Jason Tran MD^2^, Nishaant Bhambra MD^5^, Kevin Min BA^6^, Elena Dragioti PhD^7,8^, Corrado Barbui MD^9^, Jess G Fiedorowicz MD PhD^,10,11,12,13^, Corentin J. Gosling PhD^14,15,16^, Samuele Cortese MD PhD^16,17,18,19,20^, Jasmine Gandhi MD^10,12^, Gayatri Saraf MD^10,12,21^, Risa Shorr MLS^22^, Simone N Vigod MD MSc^23^, Benicio N Frey MD PhD^24,25^, Richard Delorme MD PhD^26^, Marco Solmi MD PhD^1,11,12,13,27,#^

1. SCIENCES Lab, Department of Psychiatry, University of Ottawa, Ottawa, ON, Canada
2. Department of Psychiatry, University of Toronto, Toronto, ON, Canada
3. Department of Medicine, University of Calgary, Calgary, AB, Canada
4. College of Public Health, Kent State University, Kent OH, United States
5. Department of Family Medicine, University of Ottawa, Ottawa, ON, Canada
6. Faculty of Medicine, University of Ottawa, Ottawa, ON, Canada
7. Research Laboratory Psychology of Patients, Families & Health Professionals, Department of Nursing, School of Health Sciences, University of Ioannina, Ioannina, Greece
8. Pain and Rehabilitation Centre and Department of Health, Medicine and Caring Sciences, Linköping University, Linköping, Sweden
9. WHO Collaborating Centre for Research and Training in Mental Health and Service Evaluation, Department of Neuroscience, Biomedicine and Movement Sciences, Section of Psychiatry, University of Verona, Verona, Italy
10. Department of Psychiatry, University of Ottawa, Ottawa, ON, Canada
11. Department of Mental Health, The Ottawa Hospital, Ottawa, ON, Canada
12. Ottawa Hospital Research Institute (OHRI) Clinical Epidemiology Program, University of Ottawa, Ottawa, ON, Canada
13. School of Epidemiology and Public Health, Faculty of Medicine, University of Ottawa, Ottawa, ON, Canada
14. DysCo Laboratory, F9200, Université Paris Nanterre, Nanterre, France
15. Laboratory of Psychopathology and Health Process, F92000, Université Paris Cité, Paris, France
16. Centre for Innovation in Mental Health, School of Psychology, Faculty of Environmental and Life Sciences, University of Southampton, Southampton, UK
17. Clinical and Experimental Sciences (CNS and Psychiatry), Faculty of Medicine, University of Southampton, Southampton, UK
18. Solent NHS Trust, Southampton, UK
19. Hassenfeld Children’s Hospital at NYU Langone, New York University Child Study Center, New York City, New York, USA
20. DiMePRe-J-Department of Precision and Regenerative Medicine-Jonic Area, University of Bari “Aldo Moro”, Bari, Italy
21. The Royal's Institute of Mental Health Research, Ottawa, ON, Canada
22. Library Services, The Ottawa Hospital, Ottawa, ON, Canada
23. Department of Psychiatry, Women's College Hospital and University of Toronto, Toronto, Ontario, Canada
24. Department of Psychiatry and Behavioural Neurosciences, McMaster University, Hamilton, Ontario, Canada
25. Women's Health Concerns Clinic, St. Joseph's Healthcare Hamilton, ON, Canada
26. Child and Adolescent Psychiatry Department, Robert Debré Hospital, APHP, University of Paris Cité, Paris, France
27. Department of Child and Adolescent Psychiatry, Charité Universitätsmedizin, Berlin, Germany

**# Corresponding author**

Marco Solmi, MD, PhD

University of Ottawa, Psychiatry Department - 501 Smyth Road, Ottawa, ON, Canada – +1-613-791-5555 - [msolmi@toh.ca](mailto:msolmi@toh.ca)

eTable 7. Findings across meta-analyses of adverse health outcomes of psychotropic medication use during pregnancy.

| Author, year | Trimester | Psychotropic medication | Outcome | Primary Study Type | Sample size | | Effect size | | | Heterogeneity | Significance | | | Biases | | Class and quality of evidence | |
| --- | --- | --- | --- | --- | --- | --- | --- | --- | --- | --- | --- | --- | --- | --- | --- | --- | --- |
|  |  |  |  |  | (k) | n/No | Metric | eOR  (95%CI) | ES  (95%CI) | I^2^/tau2 | p | PI sign | LS sign | SSE | ESB | CE | Q |
| Depression | | | | | | | | | | | | | | | | | |
| Xing, 2020 | Any | Antidepressant | Preterm birth | PC, RC | 10 | 16630/736843 | RR | 1.647 (1.343, 2.02) | 1.647 (1.343, 2.02) | 72.854/0.054 | 0.00000162 | N | Y | N | Y | III | L |
| Zhao, 2018 | Any | SSRI | Small for gestational age | PC, RC, CC | 10 | 90589/1927094 | RR | 1.502 (1.187, 1.901) | 1.502 (1.187, 1.901) | 66.584/0.071 | 0.000704 | N | N | Y | Y | III | L |
| Zhao, 2018 | Any | SSRI | Low birth weight | PC, RC | 10 | 41565/1381745 | RR | 1.38 (1.124, 1.694) | 1.38 (1.124, 1.694) | 57.39/0.04 | 0.00206 | N | N | N | N | IV | L |
| Kautzky, 2022 | Any | Antidepressant | Respiratory problems | PC, RC | 3 | 33/419 | OR | 2.16 (0.128, 36.386) | 2.16 (0.128, 36.386) | 33.724/0.438 | 0.361 | N | N | N | N | ns | L |
| Kautzky, 2022 | Any | Antidepressant | NICU admission | PC, RC | 7 | 96/579 | OR | 1.815 (0.936, 3.52) | 1.815 (0.936, 3.52) | 0/0 | 0.0697 | N | N | Y | N | ns | L |
| Kautzky, 2022 | Any | Antidepressant | Low 5-minute APGAR | PC, RC | 3 | 33/421 | OR | 1.391 (0.049, 39.652) | 1.391 (0.049, 39.652) | 50.333/0.845 | 0.713 | N | N | N | N | ns | L |
| Xing, 2020 | Any | Antidepressant | Low birth weight | PC, RC | 6 | 499/399694 | RR | 1.37 (0.949, 1.98) | 1.37 (0.949, 1.98) | 35.244/0.039 | 0.0789 | N | Y | N | N | ns | L |
| Kautzky, 2022 | Any | Antidepressant | Low 1-minute APGAR | PC, RC | 3 | 53/421 | OR | 1.045 (0.14, 7.812) | 1.045 (0.14, 7.812) | 52.491/0.422 | 0.934 | N | N | N | N | ns | L |
| Ross, 2013 | Any | Antidepressant | Birth weight | RC | 3 | 15299/1082231 | SMD | NA | -0.129  (-0.395, 0.136) | 76.96/0.01 | 0.171 | N | Y | N | N | ns | L |
| Ross, 2013 | Any | Antidepressant | Gestational age | PC, RC | 3 | 541/5655 | SMD | NA | -0.38 (-0.813, 0.054) | 52.76/0.013 | 0.0638 | N | Y | N | Y | ns | L |
| Wang, 2023 | Any | Antidepressant | Gestational diabetes | RC, CC | 3 | 21138/239447 | RR | 1.131 (0.973, 1.316) | 1.131 (0.973, 1.316) | 0/0 | 0.072 | N | N | N | N | ns | CL |
| Chang, 2020 | Any | SSRI | Preterm birth | PC | 4 | 42/17977 | RR | 1.459 (1.246, 1.708) | 1.459 (1.246, 1.708) | 0/0 | 0.0047 | Y | Y | N | N | IV | CL |
| Depression or anxiety | | | | | | | | | | | | | | | | | |
| Berard, 2016 | First | Paroxetine | Major congenital malformation | PC, RC, CC | 15 | 88282/2061842 | OR | 1.236 (1.093, 1.398) | 1.236 (1.093, 1.398) | 10.934/0.003 | 0.000738 | Y | N | N | N | III | L |
| Berard, 2016 | First | Paroxetine | Cardiac malformation | PC, RC, CC | 18 | 32683/2379469 | OR | 1.277 (1.108, 1.472) | 1.277 (1.108, 1.472) | 0/0 | 0.000758 | Y | N | N | N | III | L |
| Affective disorder | | | | | | | | | | | | | | | | | |
| Morales, 2018 | Any | Antidepressant | Autism | PC, RC, CC | 6 | 7747/1046603 | RR | 1.178 (0.835, 1.663) | 1.178 (0.835, 1.663) | 51.138/0.048 | 0.276 | N | N | N | N | ns | CL |
| Mental disorder | | | | | | | | | | | | | | | | | |
| Huybrechts, 2014 | Any | Antidepressant | Preterm birth | RC, PC | 11 | 12978/236750 | OR | 1.618 (1.239, 2.115) | 1.618 (1.239, 2.115) | 50.383/0.085 | 0.000417 | N | N | N | N | III | L |
| Grigoriadis, 2020 | Any | Benzodiazepine | Low birth weight | PC | 3 | 314/4071 | OR | 2.066 (0.608, 7.022) | 2.066 (0.608, 7.022) | 51.783/0.16 | 0.125 | N | Y | N | N | ns | L |
| Brown, 2017 | First | SSRI | Autism | PC, CC | 4 | 7246/652603 | OR | 1.62 (0.913, 2.877) | 1.62 (0.913, 2.877) | 36.657/0.041 | 0.0753 | N | Y | N | N | ns | L |
| Brown, 2017 | Any | SSRI | Autism | PC, CC | 5 | 7277/653657 | OR | 1.462 (0.966, 2.213) | 1.462 (0.966, 2.213) | 39.64/0.043 | 0.0639 | N | N | N | N | ns | L |
| Grigoriadis, 2020 | Any | Benzodiazepine | Preterm birth | PC | 3 | 426/4345 | OR | 1.409  (0.697, 2.849) | 1.409  (0.697, 2.849) | 0/0 | 0.171 | N | N | N | N | ns | L |
| Gao, 2018 | Any | Paroxetine | Cardiac malformation | PC | 3 | 7468/248805 | RR | 1.269 (0.605, 2.659) | 1.269 (0.605, 2.659) | 72.219/0.067 | 0.3 | N | N | N | N | ns | L |
| Gao, 2018 | Any | Paroxetine | Major congenital malformation | PC | 2 | 1122/16724 | RR | 1.17 (0.361, 3.788) | 1.17 (0.361, 3.788) | 0 | 0.339 | NA | N | NA | NA | ns | L |
| Gao, 2018 | Any | Citalopram | Major congenital malformation | PC | 2 | 1089/17470 | RR | 1.163 (0.137, 9.896) | 1.163 (0.137, 9.896) | 66.221/0.038 | 0.535 | N | N | N | N | ns | L |
| Lou, 2022 | First | SNRI | Cardiac malformation | PC, RC | 5 | 8827/222419 | RR | 1.12 (0.822, 1.526) | 1.12 (0.822, 1.526) | 0/0 | 0.367 | N | N | N | N | ns | L |
| Gao, 2018 | Any | Sertraline | Major congenital malformation | PC | 2 | 1152/16281 | RR | 1.119 (0.724, 1.729) | 1.119 (0.724, 1.729) | 0/0 | 0.188 | NA | N | NA | NA | ns | L |
| Gao, 2018 | Any | Sertraline | Cardiac malformation | PC | 3 | 7445/248362 | RR | 1.115 (0.915, 1.359) | 1.115 (0.915, 1.359) | 0/0 | 0.141 | N | N | N | N | ns | L |
| Gao, 2018 | Any | SSRI | Cardiac malformation | PC, RC | 6 | 19307/1607563 | RR | 1.092 (0.834, 1.431) | 1.092 (0.834, 1.431) | 43.804/0.02 | 0.439 | N | Y | N | N | ns | L |
| Gao, 2018 | Any | Citalopram | Cardiac malformation | PC | 2 | 594/32209 | RR | 1.083 (0.505, 2.32) | 1.083 (0.505, 2.32) | 0/0 | 0.411 | NA | N | NA | NA | ns | L |
| Grigoriadis, 2019 | Any | Benzodiazepine | Congenital malformation | PC | 3 | 3992/132308 | OR | 1.074 (0.634, 1.819) | 1.074 (0.634, 1.819) | 0/0 | 0.618 | N | N | Y | N | ns | L |
| Lou, 2022 | First | SNRI | Congenital malformation | PC, RC | 3 | 538/420827 | OR | 1.07 (0.964, 1.186) | 1.07 (0.964, 1.186) | 0/0 | 0.108 | N | N | N | N | ns | L |
| Gao, 2018 | Any | SSRI | Major congenital malformation | PC, RC | 4 | 32253/1728237 | RR | 1.066 (0.96, 1.183) | 1.066 (0.96, 1.183) | 0/0 | 0.149 | N | N | N | N | ns | L |
| Lou, 2022 | First | SNRI | Major congenital malformation | PC, RC | 2 | 410/25676 | OR | 1.017 (0.512, 2.018) | 1.017 (0.512, 2.018) | 0/0 | 0.81 | NA | N | NA | NA | ns | L |
| Gao, 2018 | Any | Fluoxetine | Cardiac malformation | PC | 3 | 7392/250794 | RR | 0.941 (0.411, 2.152) | 0.941 (0.411, 2.152) | 42.884/0.048 | 0.782 | N | N | N | N | ns | L |
| Gao, 2018 | Any | Fluoxetine | Major congenital malformation | PC | 2 | 1063/18713 | RR | 0.84 (0.616, 1.144) | 0.84 (0.616, 1.144) | 0/0 | 0.0882 | NA | N | NA | NA | ns | L |
| Halvorsen, 2019 | Third | SSRI | ADHD | CC | 2 | 2674/45948 | OR | 0.689 (0.001, 370.773) | 0.689 (0.001, 370.773) | 67.174/0.34 | 0.34 | NA | N | NA | NA | ns | CL |
| Halvorsen, 2019 | Second and third | SSRI | Autism | RC | 2 | 3135/816568 | HR | 2.156 (1.926, 2.413) | 2.156 (1.926, 2.413) | 0/0 | 0.00737 | NA | N | NA | NA | IV | CL |
| Halvorsen, 2019 | First | SSRI | Autism | CC | 4 | 7093/62954 | OR | 1.874 (1.04, 3.376) | 1.874 (1.04, 3.376) | 31.126/0.042 | 0.0426 | N | Y | N | N | IV | CL |
| Zhou, 2018 | Second and third | Antidepressant | Autism | RC | 4 | 8909/1501185 | HR | 1.783 (1.26, 2.523) | 1.783 (1.26, 2.523) | 42.66/0.024 | 0.0131 | N | Y | N | N | IV | CL |
| Halvorsen, 2019 | Second | SSRI | Autism | CC | 4 | 7057/57162 | OR | 1.725 (1.048, 2.84) | 1.725 (1.048, 2.84) | 7.351/0.01 | 0.0401 | N | Y | N | N | IV | CL |
| Zhou, 2018 | First | SSRI | Autism | RC | 3 | 20259/2262861 | OR | 1.669 (1.503, 1.854) | 1.669 (1.503, 1.854) | 0/0 | 0.00225 | Y | Y | N | N | IV | CL |
| Poels, 2018 | Any | Antipsychotic | Neuromotor deficit | PC, CC | 2 | 26/259 | RR | 1.633 (1.143, 2.333) | 1.633 (1.143, 2.333) | 0/0 | 0.0364 | NA | N | NA | NA | IV | CL |
| Halvorsen, 2019 | Any | SSRI | Autism | CC | 5 | 11811/113241 | OR | 1.595 (1.151, 2.211) | 1.595 (1.151, 2.211) | 15.038/0.012 | 0.0165 | N | Y | N | N | IV | CL |
| Kaplan, 2017 | Any | SSRI | Autism | PC, RC | 4 | 9692/1352844 | HR | 1.583 (1.147, 2.183) | 1.583 (1.147, 2.183) | 20.46/0.008 | 0.02 | N | Y | N | N | IV | CL |
| Zhou, 2018 | First | Antidepressant | Autism | RC | 5 | 11924/2015067 | OR | 1.494 (1.028, 2.171) | 1.494 (1.028, 2.171) | 61.877/0.042 | 0.0407 | N | Y | N | N | IV | CL |
| Halvorsen, 2019 | Any | SSRI | Autism | PC, RC | 4 | 2310/1178689 | HR | 1.275 (1.125, 1.445) | 1.275 (1.125, 1.445) | 0/0 | 0.0085 | Y | N | N | N | IV | CL |
| Halvorsen, 2019 | First | SSRI | ADHD | CC | 2 | 2674/45948 | OR | 1.748 (0.793, 3.853) | 1.748 (0.793, 3.853) | 0/0 | 0.0707 | NA | N | NA | NA | ns | CL |
| Grigoriadis, 2022 | Any | Benzodiazepine | Preterm birth | PC, RC | 3 | 3541/82208 | OR | 1.722 (0.177, 16.764) | 1.722 (0.177, 16.764) | 68.211/0.602 | 0.412 | N | N | N | N | ns | CL |
| Halvorsen, 2019 | Third | SSRI | Autism | CC | 4 | 7053/53739 | OR | 1.592  (0.684, 3.706) | 1.592  (0.684, 3.706) | 62.575/0.232 | 0.178 | N | Y | N | N | ns | CL |
| Halvorsen, 2019 | First | SSRI | ADHD | RC | 2 | 38546/1770655 | HR | 1.527  (0.107, 21.746) | 1.527  (0.107, 21.746) | 69.676/0.063 | 0.292 | NA | Y | NA | NA | ns | CL |
| Halvorsen, 2019 | Any | SSRI | Mental retardation | RC | 2 | 4058/224267 | HR | 1.408 (0.684, 2.895) | 1.408 (0.684, 2.895) | 0/0 | 0.105 | NA | N | NA | NA | ns | CL |
| Halvorsen, 2019 | Any | SSRI | ADHD | PC, RC | 3 | 14006/1114927 | HR | 1.325 (0.801, 2.192) | 1.325 (0.801, 2.192) | 53.038/0.021 | 0.138 | N | N | N | N | ns | CL |
| Halvorsen, 2019 | Any | SSRI | ADHD | CC | 2 | 2674/45948 | OR | 1.268 (0.028, 58.311) | 1.268 (0.028, 58.311) | 64.17/0.118 | 0.575 | NA | N | NA | NA | ns | CL |
| Zhou, 2018 | Any | Antidepressant | Autism | PC, RC | 7 | 26301/2673972 | RR | 1.136 (0.881, 1.465) | 1.136 (0.881, 1.465) | 60.837/0.043 | 0.265 | N | N | N | N | ns | CL |
| Halvorsen, 2019 | Second | SSRI | ADHD | CC | 2 | 2674/45948 | OR | 1.114 (0.081, 15.228) | 1.114 (0.081, 15.228) | 0/0 | 0.693 | NA | N | NA | NA | ns | CL |
| Halvorsen, 2019 | Second and third | SSRI | ADHD | RC | 3 | 11091/997131 | HR | 1.097 (0.542, 2.222) | 1.097 (0.542, 2.222) | 20.453/0.017 | 0.629 | N | N | N | N | ns | CL |
| Grigoriadis, 2022 | First | Hypnotic benzodiazepine | Congenital malformation | PC, RC | 3 | 9984/374366 | OR | 1.012 (0.437, 2.343) | 1.012 (0.437, 2.343) | 0/0 | 0.957 | N | N | N | N | ns | CL |
| Bipolar disorder | | | | | | | | | | | | | | | | | |
| Fornaro, 2020 | Any | Lithium | Cardiac malformation | PC, RC | 4 | 15691/1345591 | OR | 1.835 (1.211, 2.782) | 1.835 (1.211, 2.782) | 0/0 | 0.0188 | Y | Y | N | N | IV | H |
| Fornaro, 2020 | Any | Lithium | Congenital malformation | PC, RC | 3 | 951/22011 | OR | 1.944 (1.193, 3.167) | 1.944 (1.193, 3.167) | 0/0 | 0.0279 | N | Y | N | N | IV | H |
| Fornaro, 2020 | Any | Lithium | Preterm birth | PC, RC | 5 | 2143/22718 | OR | 1.906 (1.001, 3.63) | 1.906 (1.001, 3.63) | 52.981/0.15 | 0.0498 | N | Y | N | N | IV | H |
| Fornaro, 2020 | Any | Lithium | Spontaneous abortion | PC | 2 | 78/541 | OR | 1.54 (0.034, 69.725) | 1.54 (0.034, 69.725) | 0/0 | 0.387 | NA | N | NA | NA | ns | H |
| Fornaro, 2020 | First | Lithium | Cardiac mlformation | PC, RC | 4 | 15691/1345519 | OR | 1.883 (1.261, 2.813) | 1.883 (1.261, 2.813) | 0/0 | 0.0152 | Y | Y | N | N | IV | H |
| Fornaro, 2020 | First | Lithium | Congenital malformation | PC, RC | 4 | 984/22225 | OR | 1.966 (1.384, 2.791) | 1.966 (1.384, 2.791) | 0/0 | 0.00872 | N | Y | N | N | IV | H |
| Fornaro, 2020 | First | Lithium | Preterm birth | PC | 2 | 78/476 | OR | 1.268 (0.419, 3.84) | 1.268 (0.419, 3.84) | 0/0 | 0.224 | NA | N | NA | NA | ns | H |
| Fornaro, 2020 | First | Lithium | Spontaneous abortion | PC | 2 | 78/541 | OR | 1.54 (0.034, 69.725) | 1.54 (0.034, 69.725) | 0/0 | 0.387 | NA | N | NA | NA | ns | H |
| Opioid use disorder | | | | | | | | | | | | | | | | | |
| Andersen, 2020 | Any | Opioid maintenance therapy | Psychomotor tests | PC, RC | 3 | 80/268 | SMD | NA | 0.25 (-1.022, 1.523) | 15.544/0.046 | 0.486 | N | N | N | N | ns | L |
| Andersen, 2020 | Any | Opioid maintenance therapy | Cognition | PC, RC | 5 | 107/415 | SMD | NA | -0.238 (-0.609, 0.134) | 0/0 | 0.15 | N | N | Y | N | ns | L |

Legend. n=cases; N=population; CE=class of evidence (convincing (I), highly suggestive (II), suggestive (III), weak (IV)); eOR=equivalent odds ratio; NR=not reported; CI=confidence interval; ES=effect size; I2=percentage of variation across effect sizes that is due to heterogeneity rather than change; tau= tau-squared heterogeneity; k=number of studies for each factor; LS=largest study with significant effect; Q=quality measured with AMSTAR 2; SSRI=selective serotonin reuptake inhibitor; NICU=neonatal intensive care unit; APGAR=appearance, pulse, grimace, activity, and respiration; ADHD=attention deficit/hyperactivity disorder; CL=critically low; L=low; H=high.
