# Supplementary material for: Safety of psychotropic medications in pregnancy: an umbrella review
Source: Mol Psychiatry. 2024 Sep 12;30(1):327–35. doi: 10.1038/s41380-024-02697-0 (PMC11649568; doi:10.1038/s41380-024-02697-0)
Supplement: Supplementary file 4 — Supplementary material 4 [file 41380_2024_2697_MOESM4_ESM.docx]

**Supplementary material 4**

**Safety of psychotropic medications in pregnancy: an umbrella review**

Nicholas Fabiano MD^1^, Stanley Wong MD^1,2^, Arnav Gupta MD^3,4^, Jason Tran MD^2^, Nishaant Bhambra MD^5^, Kevin Min BA^6^, Elena Dragioti PhD^7,8^, Corrado Barbui MD^9^, Jess G Fiedorowicz MD PhD^,10,11,12,13^, Corentin J. Gosling PhD^14,15,16^, Samuele Cortese MD PhD^16,17,18,19,20^, Jasmine Gandhi MD^10,12^, Gayatri Saraf MD^10,12,21^, Risa Shorr MLS^22^, Simone N Vigod MD MSc^23^, Benicio N Frey MD PhD^24,25^, Richard Delorme MD PhD^26^, Marco Solmi MD PhD^1,11,12,13,27,#^

1. SCIENCES Lab, Department of Psychiatry, University of Ottawa, Ottawa, ON, Canada
2. Department of Psychiatry, University of Toronto, Toronto, ON, Canada
3. Department of Medicine, University of Calgary, Calgary, AB, Canada
4. College of Public Health, Kent State University, Kent OH, United States
5. Department of Family Medicine, University of Ottawa, Ottawa, ON, Canada
6. Faculty of Medicine, University of Ottawa, Ottawa, ON, Canada
7. Research Laboratory Psychology of Patients, Families & Health Professionals, Department of Nursing, School of Health Sciences, University of Ioannina, Ioannina, Greece
8. Pain and Rehabilitation Centre and Department of Health, Medicine and Caring Sciences, Linköping University, Linköping, Sweden
9. WHO Collaborating Centre for Research and Training in Mental Health and Service Evaluation, Department of Neuroscience, Biomedicine and Movement Sciences, Section of Psychiatry, University of Verona, Verona, Italy
10. Department of Psychiatry, University of Ottawa, Ottawa, ON, Canada
11. Department of Mental Health, The Ottawa Hospital, Ottawa, ON, Canada
12. Ottawa Hospital Research Institute (OHRI) Clinical Epidemiology Program, University of Ottawa, Ottawa, ON, Canada
13. School of Epidemiology and Public Health, Faculty of Medicine, University of Ottawa, Ottawa, ON, Canada
14. DysCo Laboratory, F9200, Université Paris Nanterre, Nanterre, France
15. Laboratory of Psychopathology and Health Process, F92000, Université Paris Cité, Paris, France
16. Centre for Innovation in Mental Health, School of Psychology, Faculty of Environmental and Life Sciences, University of Southampton, Southampton, UK
17. Clinical and Experimental Sciences (CNS and Psychiatry), Faculty of Medicine, University of Southampton, Southampton, UK
18. Solent NHS Trust, Southampton, UK
19. Hassenfeld Children’s Hospital at NYU Langone, New York University Child Study Center, New York City, New York, USA
20. DiMePRe-J-Department of Precision and Regenerative Medicine-Jonic Area, University of Bari “Aldo Moro”, Bari, Italy
21. The Royal's Institute of Mental Health Research, Ottawa, ON, Canada
22. Library Services, The Ottawa Hospital, Ottawa, ON, Canada
23. Department of Psychiatry, Women's College Hospital and University of Toronto, Toronto, Ontario, Canada
24. Department of Psychiatry and Behavioural Neurosciences, McMaster University, Hamilton, Ontario, Canada
25. Women's Health Concerns Clinic, St. Joseph's Healthcare Hamilton, ON, Canada
26. Child and Adolescent Psychiatry Department, Robert Debré Hospital, APHP, University of Paris Cité, Paris, France
27. Department of Child and Adolescent Psychiatry, Charité Universitätsmedizin, Berlin, Germany

**# Corresponding author**

Marco Solmi, MD, PhD

University of Ottawa, Psychiatry Department - 501 Smyth Road, Ottawa, ON, Canada – +1-613-791-5555 - [msolmi@toh.ca](mailto:msolmi@toh.ca)

eTable 8. Individual studies included in the meta-analysis included in the umbrella review on adverse health outcomes of psychotropic medication use during pregnancy.

| **Single Study** | **Study Design** | **Disorder examined** | **Psychotropic medication** | **Trimester of exposure** | **Adverse health outcome(s)** | **Variables adjustment** | **NOS** |
| --- | --- | --- | --- | --- | --- | --- | --- |
| Ankarfeldt,2021[(1)](https://www.zotero.org/google-docs/?sfmmCD) | RC | Mental disorder | Antidepressant | First | Congenital malformation, major congenital malformation, cardiac malformation | Data source, birth year of the offspring, maternal age, previous spontaneous abortions, previous stillbirths, smoking during pregnancy, psychiatric hospitalizations, psychiatric outpatient visits, household income, and highest completed education, comorbidities or comedication | 9 |
| Berard,2017[(2)](https://www.zotero.org/google-docs/?3iLZKN) | PC | Mental disorder | Antidepressant | First | Congenital malformation, major congenital malformation, cardiac malformation | Maternal age, welfare status, diabetes, hypertension, asthma and other medication uses | 9 |
| Huybrechts,2020[(3)](https://www.zotero.org/google-docs/?aruJq9) | RC | Mental disorder | Antidepressant | First | Congenital malformation, cardiac malformation | Maternal demographic characteristics, medical indications for duloxetine, comorbid medical conditions, obstetric characteristics/ conditions, maternal drug treatment, and measures of healthcare utilization | 9 |
| Huybrechts,2014[(4)](https://www.zotero.org/google-docs/?4pZLZQ) | RC | Mental disorder | Antidepressant | First | Cardiac malformation | Propensity scores matched | 8 |
| Kolding,2021[(5)](https://www.zotero.org/google-docs/?HioVsw) | RC | Mental disorder | Antidepressant | First | Cardiac malformation | Ethnicity, civil status, parity, age, bmi, smoking, exposure to teratogens, use of other psychotropic drugs, depression, diabetes, anti-hypertensives, anti-diabetics | 9 |
| Casper,2003[(6)](https://www.zotero.org/google-docs/?tLME5k) | RC | Depression | Antidepressant | Any | NICU admission | APGAR scores at birth | 6 |
| Suri,2004[(7)](https://www.zotero.org/google-docs/?GOBdaL) | PC | Depression | Antidepressant | Any | NICU admission | Gestational age | 6 |
| Suri,2007[(8)](https://www.zotero.org/google-docs/?ODUhI7) | PC | Depression | Antidepressant | Any | NICU admission, preterm birth | NA | 6 |
| Salisbury,2016[(9)](https://www.zotero.org/google-docs/?Th8xvc) | PC | Depression | Antidepressant | Any | NICU admission, respiratory problems | Demographic variables, depression severity | 8 |
| Yang,2017[(10)](https://www.zotero.org/google-docs/?BdO9bv) | RC | Depression | Antidepressant | Any | NICU admission, low 1-minute APGAR, low 5-minute APGAR | Preterm birth, mother’s race, age at study intake | 8 |
| Frayne,2017[(11)](https://www.zotero.org/google-docs/?H6hZPv) | RC | Depression | Antidepressant | Any | NICU admission, low 1-minute APGAR, low 5-minute APGAR, respiratory problems | Smoking, substance use, obesity, antenatal complications of preeclampsia and gestational diabetes | 9 |
| Gungor,2019[(12)](https://www.zotero.org/google-docs/?WEGlYq) | RC | Depression | Antidepressant | Any | NICU admission | NA | 6 |
| Wisner,2009[(13)](https://www.zotero.org/google-docs/?Mn1iX8) | PC | Depression | Antidepressant | Any | low 1-minute APGAR, low 5-minute APGAR, respiratory problems | Maternal age and race | 7 |
| Simon,2002[(14)](https://www.zotero.org/google-docs/?p2UxS7) | RC | Depression | Antidepressant | Any | Preterm birth, low birth weight | Maternal tobacco use, other substance use, race, and number of prior births | 6 |
| Sivojelezova,2005[(15)](https://www.zotero.org/google-docs/?dbEXiB) | PC | Depression | Antidepressant | Any | Preterm birth | Maternal age, gestational age and disease | 6 |
| Oberlander,2006[(16)](https://www.zotero.org/google-docs/?bzKAL0) | RC | Depression | Antidepressant | Any | Preterm birth | Propensity score matching | 6 |
| Lund,2009[(17)](https://www.zotero.org/google-docs/?q9V6uj) | PC | Depression | Antidepressant | Any | Preterm birth, low birth weight, gestational age | Maternal age, body mass index, smoking, a previous pregnancy | 7 |
| Hayes,2012[(18)](https://www.zotero.org/google-docs/?gc1mAd) | RC | Depression | Antidepressant | Any | Preterm birth | Maternal age, race, smoking, education, parity | 6 |
| Grzeskowiak,2012[(19)](https://www.zotero.org/google-docs/?xABIIo) | RC | Depression | Antidepressant | Any | Preterm birth, low birth weight, small for gestational age | Maternal age, SES, smoking, race, asthma, preexisting diabetes, alcohol abuse, substance abuse, hypertension, parity, epilepsy, thyroid disorder, anxiolytic use | 6 |
| El Marroun,2012[(20)](https://www.zotero.org/google-docs/?7Zp3oe) | PC | Depression | Antidepressant | Any | Preterm birth, low birth weight | Maternal age at intake, sex of the child, maternal education, ethnicity, maternal smoking and drinking habits, bmi, parity, and maternal benzodiazepine use | 7 |
| Sahingoz,2014[(21)](https://www.zotero.org/google-docs/?5U2BBa) | PC | Depression | Antidepressant | Any | Preterm birth, low birth weight | NA | 6 |
| Cantarutti,2016[(22)](https://www.zotero.org/google-docs/?MGBAtJ) | PC | Depression | Antidepressant | Any | Preterm birth, low birth weight | Maternal age, nationality, marital status, education, employment, previous miscarriages, parity, and medical conditions | 8 |
| Viktorin,2016[(23)](https://www.zotero.org/google-docs/?Un6HmO) | PC | Depression | Antidepressant | Any | Preterm birth | Mother's education, mother's BMI, parity, mother's age at pregnancy, mother's previous psychiatric history, mother's smoking status at the first visit to maternal care | 9 |
| Yonkers,2012[(24)](https://www.zotero.org/google-docs/?PlvCl5) | PC | Depression | Antidepressant | Any | Preterm birth | Age, race/ethnicity, educational level, smoking, heavy drinking, and illicit drug use during pregnancy | 8 |
| Freeman,2018[(25)](https://www.zotero.org/google-docs/?JbIOJx) | PC | Mental disorder | Benzodiazepine | Any | Preterm birth, low birth weight | Propensity score | 8 |
| Yonkers,2017[(26)](https://www.zotero.org/google-docs/?wTQHRw) | PC | Mental disorder | Benzodiazepine | Any | Preterm birth, low birth weight | Panic disorder, GAD, SSRI use, possible confounders (age, race/ethnicity, educational level, smoking, heavy drinking, illicit drug use, MDE, PTSD during pregnancy, plus the interaction of MDE with panic disorder, GAD, or PTSD and SSRI use, BMI and previous pre-term birth | 8 |
| Sutter-Dallay,2015[(27)](https://www.zotero.org/google-docs/?e3aofq) | PC | Mental disorder | Benzodiazepine | Any | Preterm birth, low birth weight | 3 other types of drugs (antidepressants, antipsychotics, and mood stabilizers), maternal age and education level, parity, presence of partner, maternal psychiatric diagnosis, unit type (adult/child psychiatry unit), and smoking prenatally | 7 |
| Chasnoff,1986[(28)](https://www.zotero.org/google-docs/?TAEliI) | PC | Opioid use disorder | Opioid maintenance therapy | Any | Cognition, psychomotor tests | NA | 6 |
| Davis,1988[(29)](https://www.zotero.org/google-docs/?gmhVYZ) | RC | Opioid use disorder | Opioid maintenance therapy | Any | Cognition, psychomotor tests | NA | 5 |
| Lifschitz,1985[(30)](https://www.zotero.org/google-docs/?HeQGC9) | PC | Opioid use disorder | Opioid maintenance therapy | Any | Cognition | NA | 6 |
| Wilson,1981[(31)](https://www.zotero.org/google-docs/?sKESDi) | PC | Opioid use disorder | Opioid maintenance therapy | Any | Cognition, psychomotor tests | NA | 6 |
| Wilson,1989[(32)](https://www.zotero.org/google-docs/?o7CclH) | PC | Opioid use disorder | Opioid maintenance therapy | Any | Cognition | NA | 6 |
| Diav-Citrin,2014[(33)](https://www.zotero.org/google-docs/?YxZq4o) | PC | Bipolar disorder | Mood stabilizer | First | Spontaneous abortion, preterm birth, low birth weight, congenital malformation, cardiac malformation | Pregnancy order, parity, previous miscarriage, previous elective termination of pregnancy, smoking 10 or more cigarettes per day, gestational age at initial contact, maternal age, bipolar disorder | 8 |
| Jacobson,1992[(34)](https://www.zotero.org/google-docs/?LtO6Gm) | PC | Bipolar disorder | Mood stabilizer | First | Spontaneous abortion, preterm birth, low birth weight, congenital malformation | NA | 8 |
| Munk-Olsen,2018[(35)](https://www.zotero.org/google-docs/?dPW7dm) | RC | Bipolar disorder | Mood stabilizer | Any, first | Preterm birth, low birth weight, congenital malformation | Maternal age at delivery (in years), primiparity, calendar year of birth, and treatment with any other psychotropic medication during pregnancy | 9 |
| Forsberg,2018[(36)](https://www.zotero.org/google-docs/?7WTNcm) | RC | Bipolar disorder | Mood stabilizer | Any | Preterm birth | Maternal level of education and social problems | 8 |
| Frayne,2018[(37)](https://www.zotero.org/google-docs/?WxXcCB) | RC | Bipolar disorder | Mood stabilizer | Any | Preterm birth | NA | 9 |
| Troyer,1993[(38)](https://www.zotero.org/google-docs/?ukaotZ) | RC | Bipolar disorder | Mood stabilizer | Any | Preterm birth | NA | 8 |
| Kallen,1983[(39)](https://www.zotero.org/google-docs/?RKYRwi) | RC | Bipolar disorder | Mood stabilizer | First | Congenital malformation, cardiac malformation | NA | 8 |
| Patorno,2017[(40)](https://www.zotero.org/google-docs/?9aNbe0) | RC | Bipolar disorder | Mood stabilizer | First | Cardiac malformation | Propensity score | 8 |
| Oberlander,2008[(41)](https://www.zotero.org/google-docs/?DYNCv0) | RC | Depression | Benzodiazepine, antidepressant | Any | Congenital malformation, major congenital malformation, cardiac malformation | Age, prenatal care visits, number of depression diagnoses, number of visits to psychiatrist, and number of visits to a physician in the year before last menstrual period, pregnancy diseases/complications diagnosed>60 days before birth, depression in the 1st trimester, and a dummy variable indicating mother filled a prescription after knowing she was pregnant | 6 |
| Ban,2014[(42)](https://www.zotero.org/google-docs/?yh1Hna) | RC | Depression | Benzodiazepine, antidepressant | Any, first | Congenital malformation, major congenital malformation, cardiac malformation | Maternal age, calendar year of birth, maternal smoking, BMI, SES | 7 |
| Milkovich,1974[(43)](https://www.zotero.org/google-docs/?cKkLhI) | PC | Mental disorder | Benzodiazepine | Any | Congenital malformation | NA | 9 |
| Clements,2015[(44)](https://www.zotero.org/google-docs/?NkkRaF) | CC | Mental disorder | Antidepressant | Any, first, second and third | Autism | Maternal age, ethnicity, median income, insurance type, child sex, birth year, maternal depression, matched on child sex, birth year, birth facility, ethnicity, insurance type, preterm versus full term | 8 |
| Croen,2011[(45)](https://www.zotero.org/google-docs/?HHWP0M) | CC | Mental disorder | Antidepressant | Any, first, second and third | Autism | Maternal age, ethnicity, education, child sex, birth weight, birth year, birth facility, maternal mental illness, matched on child sex, birth year, birth facility | 8 |
| Rai,2013[(46)](https://www.zotero.org/google-docs/?eMTfZX) | CC | Mental disorder | Antidepressant | Any, first | Autism | Parental age, maternal parity, income, education, occupation, maternal birth country, maternal mental illness, matched on child sex, birth month, birth year | 8 |
| Hviid,2013[(47)](https://www.zotero.org/google-docs/?kejdJW) | RC | Mental disorder | Antidepressant | Any, first | Autism | Maternal age, parity, country of origin, birth place, residence, education, employment, smoking, maternal mental illness, other maternal drug use | 9 |
| Boukhris,2016[(48)](https://www.zotero.org/google-docs/?liNLwk) | PC | Mental disorder | Antidepressant | Any | Autism | Maternal age at conception, living alone, social assistance, education, infant sex, infant year of birth, history of maternal psychiatric conditions, history of chronic physical conditions | 8 |
| Jimenez-Solem,2012[(49)](https://www.zotero.org/google-docs/?yxgaAO) | RC | Depression | Antidepressant | First | Cardiac malformation, major congenital malformation | Maternal age, parity, income, education, smoking and year of conception | 6 |
| Nordeng,2012[(50)](https://www.zotero.org/google-docs/?ahppUe) | PC | Depression | Antidepressant | First | Cardiac malformation, major congenital malformation, preterm birth, low birth weight | Maternal depression, age, parity, prepregnancy bmi, and of use of psychotropic drugs during pregnancy | 6 |
| Colvin,2011[(51)](https://www.zotero.org/google-docs/?V50LdX) | RC | Depression | Antidepressant | First | Cardiac malformation, major congenital malformation, low birth weight | Previous preterm birth, smoke, SEIFA, parity, maternal age | 6 |
| Malm,2011[(52)](https://www.zotero.org/google-docs/?9ZmW5h) | RC | Depression | Antidepressant | First | Cardiac malformation, major congenital malformation | Maternal age at the end of pregnancy, parity, year of pregnancy ending, marital status, smoking, other reimbursed psychiatric drug purchases, entitlement for special reimbursement for pre-pregnancy, diabetes | 6 |
| Reis,2010[(53)](https://www.zotero.org/google-docs/?i5ApzP) | RC | Depression | Antidepressant | First | Cardiac malformation, major congenital malformation, birth weight, small for gestational age | Delivery year, maternal age, parity, smoking, BMI | 7 |
| Diav-Citrin,2008[(54)](https://www.zotero.org/google-docs/?LQ3hn4) | PC | Depression | Antidepressant | First | Cardiac malformation, major congenital malformation | Type of exposure, gestational age at a call, maternal age, smoking, previous miscarriages, teratology information service origin, benzodiazepine use, other psychiatric drugs, multifetal gestation, SSRI dose | 5 |
| Alwan,2007[(55)](https://www.zotero.org/google-docs/?k3JtMt) | RC | Depression | Antidepressant | First | Cardiac malformation, major congenital malformation | Maternal race/ethnic group, maternal obesity, maternal smoking, and family income | 7 |
| Berard,2007[(56)](https://www.zotero.org/google-docs/?7bqHaY) | CC | Depression | Antidepressant | First | Cardiac malformation, major congenital malformation | Gestational and maternal age at the time of delivery, mean number of prenatal visits, visits to an obstetrician during pregnancy, pregnancy in the year before this pregnancy, diabetes, hypertension, and depression in the year before or during the pregnancy, place of residence, living alone, welfare status, calendar year, mean number of physician visits in the year before pregnancy, visits to a psychiatrists before or during pregnancy, ed visits or hospitalization in the year before or during pregnancy, number of medications (excluding antidepressants) in the year before or during pregnancy, number of different prescribers in the year before and during pregnancy | 7 |
| Cole,2007[(57)](https://www.zotero.org/google-docs/?iINbfe) | RC | Depression | Antidepressant | First | Cardiac malformation, major congenital malformation | Maternal age, infant sex, year of delivery,comorbid conditions during pregnancy (including gestational diabetes, hypertension, and cancer), dispensings of prescription drugs known or suspected to be teratogenic (ace inhibitors, aminoglycosides,androgens, anticholinergic drugs, busulfan, carbamazepine, cyclophosphamide, danazol, diethylstilbestrol,etretinate, fluconazole, indomethacin, isotretinoin,lithium, methimazole, methotrexate, misoprostol, oral corticosteroids, paramethadione, penicillamine, phenytoin, propylthiouracil, and tetracycline), and measures of health care utilization preceding pregnancy. | 6 |
| Davis,2007[(58)](https://www.zotero.org/google-docs/?QOAEIZ) | RC | Depression | Antidepressant | First | Cardiac malformation, major congenital malformation | NA | 5 |
| Louik,2007[(59)](https://www.zotero.org/google-docs/?oIGW1H) | RC | Depression | Antidepressant | First | Cardiac malformation, major congenital malformation | Maternal age, maternal race or ethnic group (self-reported), maternal education, year of last menstrual period/study centre (a composite variable), parity, first-trimester smoking status, first-trimester alcohol consumption, any family history of a birth defect, history of a cardiac defect in a first-degree relative, prepregnancy body-mass index, seizures, diabetes mellitus, hypertension, infertility, any use of folic acid, and first-trimester use of folic acid | 6 |
| Kulin,1998[(60)](https://www.zotero.org/google-docs/?oq0VHW) | PC | Depression | Antidepressant | First | Cardiac malformation, major congenital malformation | NR | 4 |
| Gavin,2009[(61)](https://www.zotero.org/google-docs/?C6LAoJ) | PC | Mental disorder | Antidepressant | Any | Preterm birth | Maternal race, age, parity, Medicaid status, smoking, and BMI | 7 |
| Mulder,2011[(62)](https://www.zotero.org/google-docs/?yk6Trj) | PC | Mental disorder | Antidepressant | Any | Preterm birth | Birth weight, mode of delivery, SES, marital status | 7 |
| Yonker,2012[(24)](https://www.zotero.org/google-docs/?BupCkS) | PC | Mental disorder | Antidepressant | Any | Preterm birth | Age, education, race, smoking, illicit drug use, number of lifetime hospitalizations, age of depressive onset, number of prior depressive episodes, PTSD, GAD, panic disorder in pregnancy, and suicidal thoughts in pregnancy | 8 |
| Wen,2006[(63)](https://www.zotero.org/google-docs/?HPZwKi) | RC | Depression | Antidepressant | Any | Birth weight, small for gestational age, low birth weight | Maternal age, receipt of provincial social assistance, drug dependence, parity, multigestation | 7 |
| Lewis,2010[(64)](https://www.zotero.org/google-docs/?aPsek2) | PC | Depression | Antidepressant | Any | Gestational age | NA | 5 |
| Malm,2015[(65)](https://www.zotero.org/google-docs/?MupwYq) | PC | Mental disorder | Antidepressant | Any | Major congenital malformation | Sex, birth period, maternal age at delivery, place of residence, marital status, parity, smoking, socioeconomic status, purchase of anxiolytics, sedative-hypnotics, or antiepileptic drugs, pre-pregnancy diabetes, and other chronic diseases | 7 |
| Jordan,2016[(66)](https://www.zotero.org/google-docs/?QDooo6) | PC | Mental disorder | Antidepressant | Any | Cardiac malformation | Smoking, SES | 6 |
| Berard,2015[(67)](https://www.zotero.org/google-docs/?Crl0bd) | PC | Mental disorder | Antidepressant | Any | Cardiac malformation | Maternal age, welfare status, diabetes, hypertension, asthma, and other medication use. | 7 |
| Pedersen,2010[(68)](https://www.zotero.org/google-docs/?DpsrRM) | PC | Mental disorder | Antidepressant | Any | Cardiac malformation | Age, calendar year, income, marriage status, tobacco smoking. | 7 |
| Chambers,1996[(69)](https://www.zotero.org/google-docs/?9pW6Yl) | PC | Depression | Antidepressant | Any | Small for gestational age | Multiparity; previous spontaneous abortion; preeclampsia, eclampsia, and hypertension; smoking status; maternal age; socioeconomic status; race; average dose of fluoxetine; gestational diabetes; use of other psychotherapeutic drugs; alcohol use; and evidence of maternal or neonatal infection near delivery | 8 |
| Toh,2009[(70)](https://www.zotero.org/google-docs/?iDv6UH) | RC | Depression | Antidepressant | Any | Small for gestational age | Region, maternal age, race/ethnicity, marital status, family income, age at menarche, diabetes mellitus, cigarette smoking,  prepregnancy body mass index, treatment with non-ssri antidepressants, number of fetuses, gravidity, and history of fertility | 5 |
| Calderon-Margalit,2009[(71)](https://www.zotero.org/google-docs/?CVRfwv) | PC | Depression | Antidepressant | Any | Small for gestational age, low birth weight | Maternal age, race, marital status, education, smoking during pregnancy, preeclampsia, parity, singleton pregnancy | 7 |
| Ramos,2010[(72)](https://www.zotero.org/google-docs/?XSaqIa) | CC | Depression | Antidepressant | Any | Small for gestational age | Number of different meds other than antidepressants, number of emergency department visits or hospitalizaitons, BMI, maternal age, race, welfare status, area of residence, parity, income, marital status, maternal weight gain, tobacco, alcohol, illicit drug use, caffeine intake, pre or gestational diabetes, pre or gestational hypertension, asthma | 6 |
| Jensen,2013[(73)](https://www.zotero.org/google-docs/?nAexcs) | RC | Depression | Antidepressant | Any | Small for gestational age | Maternal age, smoking status, social status, calendar year, sex of newborn, and use of antiepileptics,  antipsychotics, and other types of medication | 7 |
| El Marroun,2011[(20)](https://www.zotero.org/google-docs/?OIvuoC) | RC | Depression | Antidepressant | Any | Low birth weight | BMI, educational level, maternal smoking habits, maternal age, ethnicity, fetal sex, parity, and maternal use of benzodiazepines, but not maternal drinking habits and cannabis use | 7 |
| Klieger-Grossmann,2012[(74)](https://www.zotero.org/google-docs/?KLSQUK) | PC | Depression | Antidepressant | Any | Low birth weight | NA | 6 |
| Johnson,2012[(75)](https://www.zotero.org/google-docs/?9sUD8r) | PC | Mental disorder | Antipsychotic | Any | Neuromotor deficit | Gestational age | 7 |
| Peng,2013[(76)](https://www.zotero.org/google-docs/?o2pcru) | PC | Mental disorder | Antipsychotic | Any | Neuromotor deficit | NA | 7 |
| Brown,2017[(77)](https://www.zotero.org/google-docs/?t12WOb) | RC | Affective disorder | Antidepressant | Any | Autism | Gender, gestational age at delivery, maternal age, maternal psychiatric history, maternal physical history, pre-pregnancy related/delivery, severity of depression, parity, maternal income, drugs other than antidepressants | 8 |
| Harrington,2014[(78)](https://www.zotero.org/google-docs/?Btmn4k) | CC | Affective disorder | Antidepressant | First, second and third | Autism | Gender, year of birth, gestational age at delivery, education, maternal psychiatric history, alcohol or substance misuse, ethnicity or country of origin, drugs other than antidepressants | 4 |
| Malm,2016[(79)](https://www.zotero.org/google-docs/?D2CZJk) | PC | Affective disorder | Antidepressant | Any, first | Autism, ADHD | Gender, year of birth, birth weight, gestational age at delivery, maternal age, marital status, maternal psychiatric history, maternal physical histrory, pre-pregnancy related/delivery, smoking status, alcohol or substance misuse, parity, ethnicity or country of origin, residence, employment status, drugs other than antidepressants | 6 |
| Viktorin,2017[(23)](https://www.zotero.org/google-docs/?attZeW) | RC | Affective disorder | Antidepressant | Any | Autism | Maternal/paternal psychotropic medication use during pregnancy, maternal/paternal psychiatric diagnosis, birth year, maternal/paternal age at delivery, offspring sex | 8 |
| Sorensen,2013[(80)](https://www.zotero.org/google-docs/?8oTu0L) | RC | Mental disorder | Antidepressant | Any | Autism | Gender, year of birth, gestational age at delivery, maternal age, education, maternal psychiatric history, smoking status, parity, ethnicity or country of origin, residence, employment status | 8 |
| Rai,2017[(81)](https://www.zotero.org/google-docs/?Q2otBi) | PC | Mental disorder | Antidepressant | Any | Autism | Gender, year of birth, birth weight, gestational age at delivery, maternal age, education, maternal psychiatric history, paternal psychiatric, smoking, alcohol/substance misuse, parity, ethnicity/country of origin, employment status | 6 |
| Sujan,2017[(82)](https://www.zotero.org/google-docs/?jzQ3Zk) | RC | Mental disorder | Antidepressant | First, second and third | Autism, ADHD | Year of birth, maternal age, education, maternal psychiatric history, parity, ethnicity or country of origin | 8 |
| Liu,2017[(83)](https://www.zotero.org/google-docs/?siKzOw) | RC | Mental disorder | Antidepressant | Any | Mental retardation, autism | Maternal age at delivery, primiparity, maternal psychiatric history at delivery, in-patient and out-patient psychiatric treatment from 2 years before pregnancy until delivery, dispensing of other psychotropic prescriptions during pregnancy, dispensing of antiepileptic prescriptions during pregnancy, number of non-psychiatric hospital visits during pregnancy, smoking during pregnancy, place of residence, marital status, highest education, income, calendar year of delivery, and paternal psychiatric history at time of delivery | 8 |
| Gidaya,2014[(84)](https://www.zotero.org/google-docs/?zASJY7) | CC | Mental disorder | Antidepressant | Any, first, second and third | Autism | Parental age, socioeconomic status, maternal depression, other SSRI indications, duration of SSRI use, parental psychiatric history, parental history of  autoimmune disease, child sex, gestational age, birthweight, parity, obstetric complications | 5 |
| Laugesen,2013[(85)](https://www.zotero.org/google-docs/?K79pLV) | RC | Mental disorder | Antidepressant | Any | ADHD | Maternal age at birth, birth order, smoking status, marital status, maternal diagnosis of depression, maternal psychiatric diagnoses other than depression, paternal psychiatric diagnoses, maternal diseases (epilepsy, infections during pregnancy), maternal medication use during pregnancy (anxiolytics, hypnotics, sedatives), maternal BMI, sex of the child, calendar period of birth, birth weight in grams, gestational age, APGAR score at 5 minutes | 7 |
| Man,2017[(86)](https://www.zotero.org/google-docs/?141jvI) | RC | Mental disorder | Antidepressant | Any, first, second and third | ADHD | Child sex, spontaneous vaginal delivery, multiple pregnancy, birth trauma, timing of apgar score<7, birthweight, ga, mean maternal age at delivery, maternal underlying conditions,use of psychiatric drugs prepregnancy, parity, median household income | 8 |
| Figueroa,2010[(87)](https://www.zotero.org/google-docs/?ytdDlm) | CC | Mental disorder | Antidepressant | Any, first, second and third | ADHD | Gender of child, urban metropolitan area, mother teenager, mother older than 35 years, year at birth, age at last claim, age when last eligible, premature, full-term small for gestational age, fetal suffering/asphyxia, maternal psychiatric diagnosis or organic disorders, maternal drug or alcohol problem, cigarette smoking, prescription of other medication (psychotropic, antipsychotic), maternal mental health-related visit to health services. | 6 |
| Dandijnou,2019[(88)](https://www.zotero.org/google-docs/?gSW1B0) | CC | Depression | Antidepressant | Any | Gestational diabetes | First day of gestation, receipt of social assistance during pregnancy, physician-based diagnoses of maternal diseases, medication use, history of antidepressant use and health service utilization | 5 |
| Wartko,2019[(89)](https://www.zotero.org/google-docs/?sOVWIT) | RC | Depression | Antidepressant | Any | Gestational diabetes | Year of delivery, maternal age, infant sex, parity, maternal ethnicity, maternal education, medicaid, isolated gestational proteinuria, chronic hypertension, pre-pregnancy weight, gestational diabetes, medical diagnoses, medical fills during the year prior to pregnancy onset, mental health care use during the year prior to pregnancy onset, medical fills more than a year prior to pregnancy onset, mental health care more than a year prior to pregnancy onset | 7 |
| Lupatelli,2021[(90)](https://www.zotero.org/google-docs/?V5jjha) | RC | Depression | Antidepressant | Any | Gestational diabetes | Parity and marital status, maternal education, gross yearly income, smoking, alcohol use, and physical activity in the 3-month period before pregnancy, BMI at the time of pregnancy start, self-reported use in the 6-month period before pregnancy of non-steroidal anti-inflammatory medication, depressive and anxiety symptoms | 8 |
| Juric,2009[(91)](https://www.zotero.org/google-docs/?YX7aPf) | PC | Mental disorder | Benzodiazepine | First | Congenital malformation, preterm birth | Psychiatric diagnosis, demographic characteristics and concomitant psychotropic exposures | 8 |
| Diav-Citrin,1999[(92)](https://www.zotero.org/google-docs/?iF2PAd) | PC | Mental disorder | Benzodiazepine | First | Congenital malformation, preterm birth | Age, cigarette smoking, alcohol consumption, gravidity, history of previous miscarriages and pregnancy terminations, and weight gain during pregnancy | 4 |
| Huitfeldt,2020[(93)](https://www.zotero.org/google-docs/?EqYp1E) | RC | Mental disorder | Benzodiazepine | Any | Congenital malformation, preterm birth | BMI before conception, smoking, illicit drug use, alcohol intake, planned pregnancy, income, ongoing or completed education, adverse life events, sleeping and mental health issues, anxiety,and lifetime history of major depression | 7 |

Legend. NICU=neonatal intensive care unit; APGAR=appearance, pulse, grimace, activity, and respiration; ADHD=attention deficit/hyperactivity disorder; SES=socioeconomic status; BMI=body mass index; GAD=generalized anxiety disorder; SSRI=Selective serotonin reuptake inhibitor; MDE=major depressive episode; PTSD=post-traumatic stress disorder; SEIFA=Socio-Economic Indexes for Australia; PC=prospective cohort, RC=retrospective cohort, CC=case-control.

**References**

[1. Ankarfeldt MZ, Petersen J, Andersen JT, Li H, Motsko SP, Fast T, et al. Exposure to duloxetine during pregnancy and risk of congenital malformations and stillbirth: A nationwide cohort study in Denmark and Sweden. PLOS Med. 2021 Nov 22;18(11):e1003851.](https://www.zotero.org/google-docs/?5oSicH)

[2. Bérard A, Zhao JP, Sheehy O. Antidepressant use during pregnancy and the risk of major congenital malformations in a cohort of depressed pregnant women: an updated analysis of the Quebec Pregnancy Cohort. BMJ Open. 2017 Jan 1;7(1):e013372.](https://www.zotero.org/google-docs/?5oSicH)

[3. Huybrechts KF, Bateman BT, Pawar A, Bessette LG, Mogun H, Levin R, et al. Maternal and fetal outcomes following exposure to duloxetine in pregnancy: cohort study. BMJ. 2020 Feb 19;368:m237.](https://www.zotero.org/google-docs/?5oSicH)

[4. Huybrechts KF, Palmsten K, Avorn J, Cohen LS, Holmes LB, Franklin JM, et al. Antidepressant use in pregnancy and the risk of cardiac defects. N Engl J Med. 2014 Jun 19;370(25):2397–407.](https://www.zotero.org/google-docs/?5oSicH)

[5. Kolding L, Ehrenstein V, Pedersen L, Sandager P, Petersen O, Uldbjerg N, et al. Antidepressant use in pregnancy and severe cardiac malformations: Danish register-based study. BJOG Int J Obstet Gynaecol. 2021;128(12):1949–57.](https://www.zotero.org/google-docs/?5oSicH)

[6. Casper RC, Fleisher BE, Lee-Ancajas JC, Gilles A, Gaylor E, DeBattista A, et al. Follow-up of children of depressed mothers exposed or not exposed to antidepressant drugs during pregnancy. J Pediatr. 2003 Apr;142(4):402–8.](https://www.zotero.org/google-docs/?5oSicH)

[7. Suri R, Altshuler L, Hendrick V, Rasgon N, Lee E, Mintz J. The impact of depression and fluoxetine treatment on obstetrical outcome. Arch Women’s Ment Health. 2004 Jul 1;7(3):193–200.](https://www.zotero.org/google-docs/?5oSicH)

[8. Suri R, Altshuler L, Hellemann G, Burt VK, Aquino A, Mintz J. Effects of antenatal depression and antidepressant treatment on gestational age at birth and risk of preterm birth. Am J Psychiatry. 2007 Aug;164(8):1206–13.](https://www.zotero.org/google-docs/?5oSicH)

[9. Salisbury AL, O’Grady KE, Battle CL, Wisner KL, Anderson GM, Stroud LR, et al. The Roles of Maternal Depression, Serotonin Reuptake Inhibitor Treatment, and Concomitant Benzodiazepine Use on Infant Neurobehavioral Functioning Over the First Postnatal Month. Am J Psychiatry. 2016 Feb 1;173(2):147–57.](https://www.zotero.org/google-docs/?5oSicH)

[10. Yang A, Ciolino JD, Pinheiro E, Rasmussen-Torvik LJ, Sit DKY, Wisner KL. Neonatal Discontinuation Syndrome in Serotonergic Antidepressant-Exposed Neonates. J Clin Psychiatry. 2017 May;78(5):605–11.](https://www.zotero.org/google-docs/?5oSicH)

[11. Frayne J, Nguyen T, Bennett K, Allen S, Hauck Y, Liira H. The effects of gestational use of antidepressants and antipsychotics on neonatal outcomes for women with severe mental illness. Aust N Z J Obstet Gynaecol. 2017 Oct;57(5):526–32.](https://www.zotero.org/google-docs/?5oSicH)

[12. Güngör BB, Öztürk N, Atar AÖ, Aydın N. Comparison of the groups treated with mirtazapine and selective serotonine reuptake inhibitors with respect to birth outcomes and severity of psychiatric disorder. Psychiatry Clin Psychopharmacol. 2019 Oct 2;29(4):822–31.](https://www.zotero.org/google-docs/?5oSicH)

[13. Wisner KL, Sit DKY, Hanusa BH, Moses-Kolko EL, Bogen DL, Hunker DF, et al. Major depression and antidepressant treatment: impact on pregnancy and neonatal outcomes. Am J Psychiatry. 2009 May;166(5):557–66.](https://www.zotero.org/google-docs/?5oSicH)

[14. Simon GE, Cunningham ML, Davis RL. Outcomes of prenatal antidepressant exposure. Am J Psychiatry. 2002;159(12):2055–61.](https://www.zotero.org/google-docs/?5oSicH)

[15. Sivojelezova A, Shuhaiber S, Sarkissian L, Einarson A, Koren G. Citalopram use in pregnancy: Prospective comparative evaluation of pregnancy and fetal outcome. Am J Obstet Gynecol. 2005 Dec 1;193(6):2004–9.](https://www.zotero.org/google-docs/?5oSicH)

[16. Oberlander TF, Warburton W, Misri S, Aghajanian J, Hertzman C. Neonatal Outcomes After Prenatal Exposure to Selective Serotonin Reuptake Inhibitor Antidepressants and Maternal Depression Using Population-Based Linked Health Data. Arch Gen Psychiatry. 2006 Aug 1;63(8):898–906.](https://www.zotero.org/google-docs/?5oSicH)

[17. Lund N, Pedersen LH, Henriksen TB. Selective Serotonin Reuptake Inhibitor Exposure In Utero and Pregnancy Outcomes. Arch Pediatr Adolesc Med. 2009 Oct 5;163(10):949–54.](https://www.zotero.org/google-docs/?5oSicH)

[18. Hayes RM, Wu P, Shelton RC, Cooper WO, Dupont WD, Mitchel E, et al. Maternal antidepressant use and adverse outcomes: a cohort study of 228,876 pregnancies. Am J Obstet Gynecol. 2012 Jul 1;207(1):49.e1-49.e9.](https://www.zotero.org/google-docs/?5oSicH)

[19. Grzeskowiak LE, Gilbert AL, Morrison JL. Neonatal outcomes after late-gestation exposure to selective serotonin reuptake inhibitors. J Clin Psychopharmacol. 2012;32(5):615–21.](https://www.zotero.org/google-docs/?5oSicH)

[20. El Marroun H, Jaddoe VWV, Hudziak JJ, Roza SJ, Steegers EAP, Hofman A, et al. Maternal Use of Selective Serotonin Reuptake Inhibitors, Fetal Growth, and Risk of Adverse Birth Outcomes. Arch Gen Psychiatry. 2012 Jul 1;69(7):706–14.](https://www.zotero.org/google-docs/?5oSicH)

[21. Şahingöz M, Yuksel G, Karsidag C, Uguz F, Sonmez EO, Annagur BB, et al. Birth weight and preterm birth in babies of pregnant women with major depression in relation to treatment with antidepressants. J Clin Psychopharmacol. 2014;34(2):226–9.](https://www.zotero.org/google-docs/?5oSicH)

[22. Cantarutti A, Merlino L, Monzani E, Giaquinto C, Corrao G. Is the Risk of Preterm Birth and Low Birth Weight Affected by the Use of Antidepressant Agents during Pregnancy? A Population-Based Investigation. PLOS ONE. 2016 Dec 15;11(12):e0168115.](https://www.zotero.org/google-docs/?5oSicH)

[23. Viktorin A, Lichtenstein P, Lundholm C, Almqvist C, D’Onofrio BM, Larsson H, et al. Selective serotonin re-uptake inhibitor use during pregnancy: association with offspring birth size and gestational age. Int J Epidemiol. 2016 Feb 1;45(1):170–7.](https://www.zotero.org/google-docs/?5oSicH)

[24. Yonkers KA, Norwitz ER, Smith MV, Lockwood CJ, Gotman N, Luchansky E, et al. Depression and serotonin reuptake inhibitor treatment as risk factors for preterm birth. Epidemiology. 2012;23(5):677–85.](https://www.zotero.org/google-docs/?5oSicH)

[25. Freeman MP, Góez-Mogollón L, McInerney KA, Davies AC, Church TR, Sosinsky AZ, et al. Obstetrical and neonatal outcomes after benzodiazepine exposure during pregnancy: Results from a prospective registry of women with psychiatric disorders. Gen Hosp Psychiatry. 2018 Jul 1;53:73–9.](https://www.zotero.org/google-docs/?5oSicH)

[26. Yonkers KA, Gilstad-Hayden K, Forray A, Lipkind HS. Association of Panic Disorder, Generalized Anxiety Disorder, and Benzodiazepine Treatment During Pregnancy With Risk of Adverse Birth Outcomes. JAMA Psychiatry. 2017 Nov 1;74(11):1145–52.](https://www.zotero.org/google-docs/?5oSicH)

[27. Sutter-Dallay AL, Bales M, Pambrun E, Glangeaud-Freudenthal NMC, Wisner KL, Verdoux H. Impact of prenatal exposure to psychotropic drugs on neonatal outcome in infants of mothers with serious psychiatric illnesses. J Clin Psychiatry. 2015 Jul;76(7):967–73.](https://www.zotero.org/google-docs/?5oSicH)

[28. Chasnoff IJ, Burns KA, Burns WJ, Schnoll SH. Prenatal drug exposure: Effects on neonatal and infant growth and development. Neurobehav Toxicol Teratol. 1986;8(4):357–62.](https://www.zotero.org/google-docs/?5oSicH)

[29. Davis DD, Templer DI. Neurobehavioral functioning in children exposed to narcotics in Utero. Addict Behav. 1988 Jan 1;13(3):275–83.](https://www.zotero.org/google-docs/?5oSicH)

[30. Lifschitz MH, Wilson GS, Smith EO, Desmond MM. Factors Affecting Head Growth and Intellectual Function in Children of Drug Addicts. Pediatrics. 1985 Feb 1;75(2):269–74.](https://www.zotero.org/google-docs/?5oSicH)

[31. Wilson GS, Desmond MM, Wait RB. Follow-up of methadone-treated and untreated narcotic-dependent women and their infants: Health, developmental, and social implications. J Pediatr. 1981 May 1;98(5):716–22.](https://www.zotero.org/google-docs/?5oSicH)

[32. Wilson GS. Clinical Studies of Infants and Children Exposed Prenatally to Heroin. Ann N Y Acad Sci. 1989;562(1):183–94.](https://www.zotero.org/google-docs/?5oSicH)

[33. Diav-Citrin O, Shechtman S, Tahover E, Finkel-Pekarsky V, Arnon J, Kennedy D, et al. Pregnancy Outcome Following In Utero Exposure to Lithium: A Prospective, Comparative, Observational Study. Am J Psychiatry. 2014 Jul;171(7):785–94.](https://www.zotero.org/google-docs/?5oSicH)

[34. Jacobson SJ, Ceolin L, Kaur P, Pastuszak A, Einarson T, Koren G, et al. Prospective multicentre study of pregnancy outcome after lithium exposure during first trimester. The Lancet. 1992 Feb 29;339(8792):530–3.](https://www.zotero.org/google-docs/?5oSicH)

[35. Munk-Olsen T, Liu X, Viktorin A, Brown HK, Di Florio A, D’Onofrio BM, et al. Maternal and infant outcomes associated with lithium use in pregnancy: an international collaborative meta-analysis of six cohort studies. Lancet Psychiatry. 2018 Aug 1;5(8):644–52.](https://www.zotero.org/google-docs/?5oSicH)

[36. Forsberg L, Adler M, Römer Ek I, Ljungdahl M, Navér L, Gustafsson L, et al. Maternal mood disorders and lithium exposure in utero were not associated with poor cognitive development during childhood. Acta Paediatr. 2018;107(8):1379–88.](https://www.zotero.org/google-docs/?5oSicH)

[37. Frayne J, Nguyen T, Mok T, Hauck Y, Liira H. Lithium exposure during pregnancy: outcomes for women who attended a specialist antenatal clinic. J Psychosom Obstet Gynecol. 2018 Jul 3;39(3):211–9.](https://www.zotero.org/google-docs/?5oSicH)

[38. Troyer WA, Pereira GR, Lannon RA, Belik J, Yoder MC. Association of maternal lithium exposure and premature delivery. J Perinatol Off J Calif Perinat Assoc. 1993;13(2):123–7.](https://www.zotero.org/google-docs/?5oSicH)

[39. Källén B, Tandberg A. Lithium and pregnancy. Acta Psychiatr Scand. 1983;68(2):134–9.](https://www.zotero.org/google-docs/?5oSicH)

[40. Patorno E, Huybrechts KF, Bateman BT, Cohen JM, Desai RJ, Mogun H, et al. Lithium Use in Pregnancy and the Risk of Cardiac Malformations. N Engl J Med. 2017 Jun 8;376(23):2245–54.](https://www.zotero.org/google-docs/?5oSicH)

[41. Oberlander TF, Warburton W, Misri S, Riggs W, Aghajanian J, Hertzman C. Major congenital malformations following prenatal exposure to serotonin reuptake inhibitors and benzodiazepines using population-based health data. Birth Defects Res B Dev Reprod Toxicol. 2008 Feb;83(1):68–76.](https://www.zotero.org/google-docs/?5oSicH)

[42. Ban L, West J, Gibson JE, Fiaschi L, Sokal R, Doyle P, et al. First trimester exposure to anxiolytic and hypnotic drugs and the risks of major congenital anomalies: a United Kingdom population-based cohort study. PloS One. 2014;9(6):e100996.](https://www.zotero.org/google-docs/?5oSicH)

[43. Milkovich L, van den Berg BJ. Effects of prenatal meprobamate and chlordiazepoxide hydrochloride on human embryonic and fetal development. N Engl J Med. 1974 Dec 12;291(24):1268–71.](https://www.zotero.org/google-docs/?5oSicH)

[44. Clements CC, Castro VM, Blumenthal SR, Rosenfield HR, Murphy SN, Fava M, et al. Prenatal antidepressant exposure is associated with risk for attention-deficit hyperactivity disorder but not autism spectrum disorder in a large health system. Mol Psychiatry. 2015 Jun;20(6):727–34.](https://www.zotero.org/google-docs/?5oSicH)

[45. Croen LA, Grether JK, Yoshida CK, Odouli R, Hendrick V. Antidepressant use during pregnancy and childhood autism spectrum disorders. Arch Gen Psychiatry. 2011 Nov;68(11):1104–12.](https://www.zotero.org/google-docs/?5oSicH)

[46. Rai D, Lee BK, Dalman C, Golding J, Lewis G, Magnusson C. Parental depression, maternal antidepressant use during pregnancy, and risk of autism spectrum disorders: population based case-control study. BMJ. 2013 Apr 19;346:f2059.](https://www.zotero.org/google-docs/?5oSicH)

[47. Hviid A, Melbye M, Pasternak B. Use of selective serotonin reuptake inhibitors during pregnancy and risk of autism. N Engl J Med. 2013 Dec 19;369(25):2406–15.](https://www.zotero.org/google-docs/?5oSicH)

[48. Boukhris T, Sheehy O, Mottron L, Bérard A. Antidepressant Use During Pregnancy and the Risk of Autism Spectrum Disorder in Children. JAMA Pediatr. 2016 Feb;170(2):117–24.](https://www.zotero.org/google-docs/?5oSicH)

[49. Jimenez-Solem E, Andersen JT, Petersen M, Broedbaek K, Jensen JK, Afzal S, et al. Exposure to selective serotonin reuptake inhibitors and the risk of congenital malformations: a nationwide cohort study. BMJ Open. 2012;2(3):e001148.](https://www.zotero.org/google-docs/?5oSicH)

[50. Nordeng H, van Gelder MMHJ, Spigset O, Koren G, Einarson A, Eberhard-Gran M. Pregnancy outcome after exposure to antidepressants and the role of maternal depression: results from the Norwegian Mother and Child Cohort Study. J Clin Psychopharmacol. 2012 Apr;32(2):186–94.](https://www.zotero.org/google-docs/?5oSicH)

[51. Colvin L, Slack-Smith L, Stanley FJ, Bower C. Dispensing patterns and pregnancy outcomes for women dispensed selective serotonin reuptake inhibitors in pregnancy. Birt Defects Res A Clin Mol Teratol. 2011 Mar;91(3):142–52.](https://www.zotero.org/google-docs/?5oSicH)

[52. Malm H, Artama M, Gissler M, Ritvanen A. Selective serotonin reuptake inhibitors and risk for major congenital anomalies. Obstet Gynecol. 2011 Jul;118(1):111–20.](https://www.zotero.org/google-docs/?5oSicH)

[53. Reis M, Källén B. Delivery outcome after maternal use of antidepressant drugs in pregnancy: an update using Swedish data. Psychol Med. 2010 Oct;40(10):1723–33.](https://www.zotero.org/google-docs/?5oSicH)

[54. Diav-Citrin O, Shechtman S, Weinbaum D, Wajnberg R, Avgil M, Di Gianantonio E, et al. Paroxetine and fluoxetine in pregnancy: a prospective, multicentre, controlled, observational study. Br J Clin Pharmacol. 2008 Nov;66(5):695–705.](https://www.zotero.org/google-docs/?5oSicH)

[55. Alwan S, Reefhuis J, Rasmussen SA, Olney RS, Friedman JM, National Birth Defects Prevention Study. Use of selective serotonin-reuptake inhibitors in pregnancy and the risk of birth defects. N Engl J Med. 2007 Jun 28;356(26):2684–92.](https://www.zotero.org/google-docs/?5oSicH)

[56. Bérard A, Ramos E, Rey E, Blais L, St-André M, Oraichi D. First trimester exposure to paroxetine and risk of cardiac malformations in infants: the importance of dosage. Birth Defects Res B Dev Reprod Toxicol. 2007 Feb;80(1):18–27.](https://www.zotero.org/google-docs/?5oSicH)

[57. Cole JA, Ephross SA, Cosmatos IS, Walker AM. Paroxetine in the first trimester and the prevalence of congenital malformations. Pharmacoepidemiol Drug Saf. 2007 Oct;16(10):1075–85.](https://www.zotero.org/google-docs/?5oSicH)

[58. Davis RL, Rubanowice D, McPhillips H, Raebel MA, Andrade SE, Smith D, et al. Risks of congenital malformations and perinatal events among infants exposed to antidepressant medications during pregnancy. Pharmacoepidemiol Drug Saf. 2007 Oct;16(10):1086–94.](https://www.zotero.org/google-docs/?5oSicH)

[59. Louik C, Lin AE, Werler MM, Hernández-Díaz S, Mitchell AA. First-trimester use of selective serotonin-reuptake inhibitors and the risk of birth defects. N Engl J Med. 2007 Jun 28;356(26):2675–83.](https://www.zotero.org/google-docs/?5oSicH)

[60. Kulin NA, Pastuszak A, Sage SR, Schick-Boschetto B, Spivey G, Feldkamp M, et al. Pregnancy outcome following maternal use of the new selective serotonin reuptake inhibitors: a prospective controlled multicenter study. JAMA. 1998 Feb 25;279(8):609–10.](https://www.zotero.org/google-docs/?5oSicH)

[61. Gavin AR, Holzman C, Siefert K, Tian Y. Maternal Depressive Symptoms, Depression, and Psychiatric Medication Use in Relation to Risk of Preterm Delivery. Womens Health Issues. 2009 Sep 1;19(5):325–34.](https://www.zotero.org/google-docs/?5oSicH)

[62. Mulder EJH, Ververs FF, de Heus R, Visser GHA. Selective Serotonin Reuptake Inhibitors Affect Neurobehavioral Development in the Human Fetus. Neuropsychopharmacology. 2011 Sep;36(10):1961–71.](https://www.zotero.org/google-docs/?5oSicH)

[63. Wen SW, Yang Q, Garner P, Fraser W, Olatunbosun O, Nimrod C, et al. Selective serotonin reuptake inhibitors and adverse pregnancy outcomes. Am J Obstet Gynecol. 2006 Apr;194(4):961–6.](https://www.zotero.org/google-docs/?5oSicH)

[64. Lewis AJ, Galbally M, Opie G, Buist A. Neonatal growth outcomes at birth and one month postpartum following in utero exposure to antidepressant medication. Aust N Z J Psychiatry. 2010 May;44(5):482–7.](https://www.zotero.org/google-docs/?5oSicH)

[65. Malm H, Sourander A, Gissler M, Gyllenberg D, Hinkka-Yli-Salomäki S, McKeague IW, et al. Pregnancy Complications Following Prenatal Exposure to SSRIs or Maternal Psychiatric Disorders: Results From Population-Based National Register Data. Am J Psychiatry. 2015 Dec;172(12):1224–32.](https://www.zotero.org/google-docs/?5oSicH)

[66. Jordan S, Morris JK, Davies GI, Tucker D, Thayer DS, Luteijn JM, et al. Selective Serotonin Reuptake Inhibitor (SSRI) Antidepressants in Pregnancy and Congenital Anomalies: Analysis of Linked Databases in Wales, Norway and Funen, Denmark. PLOS ONE. 2016 Dec 1;11(12):e0165122.](https://www.zotero.org/google-docs/?5oSicH)

[67. Bérard A, Zhao JP, Sheehy O. Sertraline use during pregnancy and the risk of major malformations. Am J Obstet Gynecol. 2015 Jun 1;212(6):795.e1-795.e12.](https://www.zotero.org/google-docs/?5oSicH)

[68. Pedersen LH, Henriksen TB, Vestergaard M, Olsen J, Bech BH. Selective serotonin reuptake inhibitors in pregnancy and congenital malformations: population based cohort study. BMJ. 2009 Sep 23;339:b3569.](https://www.zotero.org/google-docs/?5oSicH)

[69. Chambers CD, Johnson KA, Dick LM, Felix RJ, Jones KL. Birth outcomes in pregnant women taking fluoxetine. N Engl J Med. 1996;335(14):1010–5.](https://www.zotero.org/google-docs/?5oSicH)

[70. Toh S, Mitchell AA, Louik C, Werler MM, Chambers CD, Hernández-Díaz S. Antidepressant use during pregnancy and the risk of preterm delivery and fetal growth restriction. J Clin Psychopharmacol. 2009;29(6):555–60.](https://www.zotero.org/google-docs/?5oSicH)

[71. Calderon-Margalit R, Qiu C, Ornoy A, Siscovick DS, Williams MA. Risk of preterm delivery and other adverse perinatal outcomes in relation to maternal use of psychotropic medications during pregnancy. Am J Obstet Gynecol. 2009 Dec 1;201(6):579.e1-579.e8.](https://www.zotero.org/google-docs/?5oSicH)

[72. Ramos É, St-André M, Bérard A. Association between antidepressant use during pregnancy and infants born small for gestational age. Can J Psychiatry. 2010;55(10):643–52.](https://www.zotero.org/google-docs/?5oSicH)

[73. Jensen HM, Grøn R, Lidegaard Ø, Pedersen LH, Andersen PK, Kessing LV. The effects of maternal depression and use of antidepressants during pregnancy on risk of a child small for gestational age. Psychopharmacology (Berl). 2013 Jul 1;228(2):199–205.](https://www.zotero.org/google-docs/?5oSicH)

[74. Klieger-Grossmann C, Weitzner B, Panchaud A, Pistelli A, Einarson T, Koren G, et al. Pregnancy Outcomes Following Use of Escitalopram: A Prospective Comparative Cohort Study. J Clin Pharmacol. 2012;52(5):766–70.](https://www.zotero.org/google-docs/?5oSicH)

[75. Johnson KC, LaPrairie JL, Brennan PA, Stowe ZN, Newport DJ. Prenatal Antipsychotic Exposure and Neuromotor Performance During Infancy. Arch Gen Psychiatry. 2012 Aug;69(8):787–94.](https://www.zotero.org/google-docs/?5oSicH)

[76. Peng M, Gao K, Ding Y, Ou J, Calabrese JR, Wu R, et al. Effects of prenatal exposure to atypical antipsychotics on postnatal development and growth of infants: a case-controlled, prospective study. Psychopharmacology (Berl). 2013 Aug;228(4):577–84.](https://www.zotero.org/google-docs/?5oSicH)

[77. Brown HK, Ray JG, Wilton AS, Lunsky Y, Gomes T, Vigod SN. Association Between Serotonergic Antidepressant Use During Pregnancy and Autism Spectrum Disorder in Children. JAMA. 2017 Apr 18;317(15):1544–52.](https://www.zotero.org/google-docs/?5oSicH)

[78. Harrington RA, Lee LC, Crum RM, Zimmerman AW, Hertz-Picciotto I. Prenatal SSRI use and offspring with autism spectrum disorder or developmental delay. Pediatrics. 2014 May;133(5):e1241-1248.](https://www.zotero.org/google-docs/?5oSicH)

[79. Malm H, Brown AS, Gissler M, Gyllenberg D, Hinkka-Yli-Salomäki S, McKeague IW, et al. Gestational Exposure to Selective Serotonin Reuptake Inhibitors and Offspring Psychiatric Disorders: A National Register-Based Study. J Am Acad Child Adolesc Psychiatry. 2016 May;55(5):359–66.](https://www.zotero.org/google-docs/?5oSicH)

[80. Sørensen MJ, Grønborg TK, Christensen J, Parner ET, Vestergaard M, Schendel D, et al. Antidepressant exposure in pregnancy and risk of autism spectrum disorders. Clin Epidemiol. 2013;5:449–59.](https://www.zotero.org/google-docs/?5oSicH)

[81. Rai D, Lee BK, Dalman C, Newschaffer C, Lewis G, Magnusson C. Antidepressants during pregnancy and autism in offspring: population based cohort study. BMJ. 2017 Jul 19;358:j2811.](https://www.zotero.org/google-docs/?5oSicH)

[82. Sujan AC, Rickert ME, Öberg AS, Quinn PD, Hernández-Díaz S, Almqvist C, et al. Associations of Maternal Antidepressant Use During the First Trimester of Pregnancy With Preterm Birth, Small for Gestational Age, Autism Spectrum Disorder, and Attention-Deficit/Hyperactivity Disorder in Offspring. JAMA. 2017 Apr 18;317(15):1553–62.](https://www.zotero.org/google-docs/?5oSicH)

[83. Liu X, Agerbo E, Ingstrup KG, Musliner K, Meltzer-Brody S, Bergink V, et al. Antidepressant use during pregnancy and psychiatric disorders in offspring: Danish nationwide register based cohort study. BMJ. 2017 Sep 6;358:j3668.](https://www.zotero.org/google-docs/?5oSicH)

[84. Gidaya NB, Lee BK, Burstyn I, Yudell M, Mortensen EL, Newschaffer CJ. In utero exposure to selective serotonin reuptake inhibitors and risk for autism spectrum disorder. J Autism Dev Disord. 2014 Oct;44(10):2558–67.](https://www.zotero.org/google-docs/?5oSicH)

[85. Laugesen K, Olsen MS, Telén Andersen AB, Frøslev T, Sørensen HT. In utero exposure to antidepressant drugs and risk of attention deficit hyperactivity disorder: a nationwide Danish cohort study. BMJ Open. 2013 Sep 20;3(9):e003507.](https://www.zotero.org/google-docs/?5oSicH)

[86. Man KKC, Chan EW, Ip P, Coghill D, Simonoff E, Chan PKL, et al. Prenatal antidepressant use and risk of attention-deficit/hyperactivity disorder in offspring: population based cohort study. BMJ. 2017 May 31;357:j2350.](https://www.zotero.org/google-docs/?5oSicH)

[87. Figueroa R. Use of antidepressants during pregnancy and risk of attention-deficit/hyperactivity disorder in the offspring. J Dev Behav Pediatr JDBP. 2010 Oct;31(8):641–8.](https://www.zotero.org/google-docs/?5oSicH)

[88. Dandjinou M, Sheehy O, Bérard A. Antidepressant use during pregnancy and the risk of gestational diabetes mellitus: a nested case-control study. BMJ Open. 2019 Oct 1;9(9):e025908.](https://www.zotero.org/google-docs/?5oSicH)

[89. Wartko PD, Weiss NS, Enquobahrie DA, Chan KCG, Stephenson-Famy A, Mueller BA, et al. Antidepressant continuation in pregnancy and risk of gestational diabetes. Pharmacoepidemiol Drug Saf. 2019 Sep;28(9):1194–203.](https://www.zotero.org/google-docs/?5oSicH)

[90. Lupattelli A, Barone-Adesi F, Nordeng H. Association between antidepressant use in pregnancy and gestational diabetes mellitus: Results from the Norwegian Mother, Father and Child Cohort Study. Pharmacoepidemiol Drug Saf. 2022 Feb;31(2):247–56.](https://www.zotero.org/google-docs/?5oSicH)

[91. Juric S, Newport DJ, Ritchie JC, Galanti M, Stowe ZN. Zolpidem (Ambien) in pregnancy: placental passage and outcome. Arch Womens Ment Health. 2009 Dec;12(6):441–6.](https://www.zotero.org/google-docs/?5oSicH)

[92. Diav-Citrin O, Okotore B, Lucarelli K, Koren G. Zopiclone use during pregnancy. Can Fam Physician Med Fam Can. 2000 Jan;46:63–4.](https://www.zotero.org/google-docs/?5oSicH)

[93. Huitfeldt A, Sundbakk LM, Skurtveit S, Handal M, Nordeng H. Associations of Maternal Use of Benzodiazepines or Benzodiazepine-like Hypnotics During Pregnancy With Immediate Pregnancy Outcomes in Norway. JAMA Netw Open. 2020 Jun 1;3(6):e205860.](https://www.zotero.org/google-docs/?5oSicH)
