# Supplementary material for: Safety of psychotropic medications in pregnancy: an umbrella review
Source: Mol Psychiatry. 2024 Sep 12;30(1):327–35. doi: 10.1038/s41380-024-02697-0 (PMC11649568; doi:10.1038/s41380-024-02697-0)
Supplement: Supplementary file 5 — Supplementary material 5 [file 41380_2024_2697_MOESM5_ESM.docx]

**Supplementary material 5**

**Safety of psychotropic medications in pregnancy: an umbrella review**

Nicholas Fabiano MD^1^, Stanley Wong MD^1,2^, Arnav Gupta MD^3,4^, Jason Tran MD^2^, Nishaant Bhambra MD^5^, Kevin Min BA^6^, Elena Dragioti PhD^7,8^, Corrado Barbui MD^9^, Jess G Fiedorowicz MD PhD^,10,11,12,13^, Corentin J. Gosling PhD^14,15,16^, Samuele Cortese MD PhD^16,17,18,19,20^, Jasmine Gandhi MD^10,12^, Gayatri Saraf MD^10,12,21^, Risa Shorr MLS^22^, Simone N Vigod MD MSc^23^, Benicio N Frey MD PhD^24,25^, Richard Delorme MD PhD^26^, Marco Solmi MD PhD^1,11,12,13,27,#^

1. SCIENCES Lab, Department of Psychiatry, University of Ottawa, Ottawa, ON, Canada
2. Department of Psychiatry, University of Toronto, Toronto, ON, Canada
3. Department of Medicine, University of Calgary, Calgary, AB, Canada
4. College of Public Health, Kent State University, Kent OH, United States
5. Department of Family Medicine, University of Ottawa, Ottawa, ON, Canada
6. Faculty of Medicine, University of Ottawa, Ottawa, ON, Canada
7. Research Laboratory Psychology of Patients, Families & Health Professionals, Department of Nursing, School of Health Sciences, University of Ioannina, Ioannina, Greece
8. Pain and Rehabilitation Centre and Department of Health, Medicine and Caring Sciences, Linköping University, Linköping, Sweden
9. WHO Collaborating Centre for Research and Training in Mental Health and Service Evaluation, Department of Neuroscience, Biomedicine and Movement Sciences, Section of Psychiatry, University of Verona, Verona, Italy
10. Department of Psychiatry, University of Ottawa, Ottawa, ON, Canada
11. Department of Mental Health, The Ottawa Hospital, Ottawa, ON, Canada
12. Ottawa Hospital Research Institute (OHRI) Clinical Epidemiology Program, University of Ottawa, Ottawa, ON, Canada
13. School of Epidemiology and Public Health, Faculty of Medicine, University of Ottawa, Ottawa, ON, Canada
14. DysCo Laboratory, F9200, Université Paris Nanterre, Nanterre, France
15. Laboratory of Psychopathology and Health Process, F92000, Université Paris Cité, Paris, France
16. Centre for Innovation in Mental Health, School of Psychology, Faculty of Environmental and Life Sciences, University of Southampton, Southampton, UK
17. Clinical and Experimental Sciences (CNS and Psychiatry), Faculty of Medicine, University of Southampton, Southampton, UK
18. Solent NHS Trust, Southampton, UK
19. Hassenfeld Children’s Hospital at NYU Langone, New York University Child Study Center, New York City, New York, USA
20. DiMePRe-J-Department of Precision and Regenerative Medicine-Jonic Area, University of Bari “Aldo Moro”, Bari, Italy
21. The Royal's Institute of Mental Health Research, Ottawa, ON, Canada
22. Library Services, The Ottawa Hospital, Ottawa, ON, Canada
23. Department of Psychiatry, Women's College Hospital and University of Toronto, Toronto, Ontario, Canada
24. Department of Psychiatry and Behavioural Neurosciences, McMaster University, Hamilton, Ontario, Canada
25. Women's Health Concerns Clinic, St. Joseph's Healthcare Hamilton, ON, Canada
26. Child and Adolescent Psychiatry Department, Robert Debré Hospital, APHP, University of Paris Cité, Paris, France
27. Department of Child and Adolescent Psychiatry, Charité Universitätsmedizin, Berlin, Germany

**# Corresponding author**

Marco Solmi, MD, PhD

University of Ottawa, Psychiatry Department - 501 Smyth Road, Ottawa, ON, Canada – +1-613-791-5555 - [msolmi@toh.ca](mailto:msolmi@toh.ca)

**eMethods**

*Search Strategy and Inclusion Criteria*

Peer-reviewed systematic reviews with meta-analysis of observational studies with a cohort, case-control or nested case-control study design which investigated the association between psychotropic medications (chapters N05, N05A, N05B, N05C, N06, N06A, N06B, N06C, N06D, and N07B in the Anatomical Therapeutic Chemical (ATC) World Health Organization (WHO) database, eTable 1) use and any adverse outcome in pregnant people of any age, controlling for the presence of a psychiatric condition (i.e., confounding by indication) were included, either via matching or statistical adjustment. We did not include conditions such as epilepsy which may be treated with psychotropic medications (i.e., valproic acid or lamotrigine for the treatment of a non-mental disorder). If multiple meta-analyses measured the same population, exposure (i.e., medication, trimester), control group, and outcome, we selected the meta-analysis with the largest number of studies. For each included meta-analysis, we considered the main analysis for all primary and secondary reported outcomes.

The exclusion criteria were as follows: (1) meta-analyses exclusively including randomised controlled trials (RCTs) or a cross-sectional study design, (2) meta-analyses of studies identified with non-systematic reviews, (3) individual patient data meta-analyses reporting results only of the one-stage approach, (4) meta-analyses which provide insufficient data for quantitative synthesis and (5) meta-analyses focusing not on a mental disorder, however using a psychotropic medication (i.e., epilepsy and anticonvulsants). For meta-analyses that pooled both RCTs and observational studies we considered observational studies only.

*Data extraction*

Blinded pairs from among six investigators (NF, SW, AG, JT, NB, KM) independently extracted relevant data from the included meta-analyses and individual studies into a Microsoft Excel spreadsheet which was designed a priori, with all discrepancies resolved through consensus. The adverse health outcomes due to exposure to psychotropic medications were extracted as defined by the original authors. For each meta-analysis, we extracted the standard identifier (PMID or DOI), first author name, year of publication, country of corresponding author, type of psychotropic medication, disorder examined, study design, age of participants, adverse health outcomes, exposure or non-exposure status, number of included studies, and total sample size. For each individual study, we extracted the standard identifier (PMID or DOI), first author name, year of publication, country of study location, study design, number of participants (cases and controls in case-control studies or total population in cohort studies), number of events and non-events in those exposed and not exposed in cohort studies, duration of follow-up, reported adjusted (or unadjusted) effect size with 95%CI, and the number and nature of adjustments.

Quality Assessment

A Measurement Tool to Assess Systematic Reviews (AMSTAR) 2[(1)](https://www.zotero.org/google-docs/?UCEXpe) was used by six investigators (NF, SW, AG, JT, NB, KM) to assess the methodological quality of each included meta-analysis and the Newcastle-Ottawa Scale (NOS)[(2)](https://www.zotero.org/google-docs/?jej9tP) was used to measure the quality of individual studies with discrepancies for each criterion reached through consensus. If the NOS score was reported within the meta-analysis, it was instead extracted.

*Statistical analysis*

We used the I^2^ statistic and Tau to assess heterogeneity[(3)](https://www.zotero.org/google-docs/?qzp8Wc) along with the 95% prediction intervals for the summary effect sizes to determine the range within which the results of a future study might lie.[(4)](https://www.zotero.org/google-docs/?AnlZxi) The presence of small-study effects was tested both with Egger's regression asymmetry test (p≤0.10)[(5)](https://www.zotero.org/google-docs/?XKZdPG) and by examining if the random-effects summary effect size is larger than that of the largest study for each association.[(6,7)](https://www.zotero.org/google-docs/?w4XBZ6)

The presence of excess significance bias was determined by using the Test for Excess Statistical Significance (TESS) and the Proportion of Statistical Significance Test (PSST).[(8)](https://www.zotero.org/google-docs/?W9PJ00) Both TESS and PSST have desirable statistical properties: adequate control of Type I errors and high statistical power, and they take inconsistency into account. The presence of excess significance bias is considered present if either TESS or PSST was greater than the Z-score of 1.645 (≥95th percentile).

*Sensitivity analysis*

Sensitivity analyses were performed to determine whether the credibility of evidence varied based on study type (restricted to cohort studies) or for studies which adjusted for covariates beyond just mental disorders. These sensitivity analyses were only performed for the associations with the highest class of evidence as determined by the main analysis.

**eResults**

*Study characteristics*

The meta-analyses reported numerous associations between the use of psychotropic medication during pregnancy and adverse health outcomes (eTable 7; supplementary material 3): congenital malformations (n=5), major congenital malformations (n=7), cardiac malformations (n=9), neonatal intensive care unit (NICU) admission (n=1), low APGAR scores (n=2), respiratory problems (n=1), preterm birth (n=7), low birth weight (n=4), small for gestational age (n=1), gestational age (n=2), cognition (n=1), psychomotor tests (n=1), autism (n=14), neuromotor deficit (n=1), ADHD (n=7), mental retardation (n=1), spontaneous abortion (n=1), and gestational diabetes (n=1).

*Other details of psychotropic medications, adjustment of analyses, and quality of individual studies*

The details about the psychotropic medications, the variables that analyses are adjusted for and the quality of individual studies included in eligible meta-analyses are reported in eTable 8 (supplementary material 4). Of the 93 unique individual studies included in the eligible meta-analyses, 77 were cohort studies and 16 were case-control studies. Of these, the psychotropic medication class examined was antidepressants in 71 studies, benzodiazepines in nine studies, mood stabilisers in eight studies, opioid maintenance therapy in five studies, and antipsychotics in two studies. The patients were diagnosed with depression in 47 studies, a general mental disorder in 34 studies, bipolar disorder in eight studies, and an affective disorder in four studies. Analyses were adjusted in 76 studies and unadjusted in 17 studies. The median NOS score was 7 (IQR 6-8).

*Strength of evidence for associations between psychotropic drugs and adverse health outcomes*

*Antidepressants*

The remaining associations did not have statistically significant effects: ADHD in children, gestational diabetes, low APGAR scores at birth, NICU admission, respiratory problems in any pregnant people with any mental disorder or depression receiving antidepressants (SSRIs, SNRIs, paroxetine, citalopram, fluoxetine, or sertraline) during any (and specific) trimesters of pregnancy (eTable 7; supplementary material 3).

*Mood stabilisers*

The remaining associations did not reach statistical significance: preterm birth in pregnant people with bipolar disorder receiving lithium in the first trimester or spontaneous abortion in pregnant people with bipolar disorder receiving lithium in any trimester (eTable 7; supplementary material 3).

*Benzodiazepines*

Associations examined included low birth weight, preterm birth, and congenital malformation in pregnant people with any mental disorder receiving benzodiazepines during any trimester of pregnancy; preterm birth in pregnant people with any mental disorder receiving hypnotic benzodiazepines during any trimester of pregnancy; and congenital malformations in pregnant people with any mental disorder receiving hypnotic benzodiazepines during first trimester of pregnancy.

*Opioid maintenance therapy*

These associations included psychomotor functioning and cognition in pregnant people with opioid use disorder currently undergoing opioid maintenance therapy during any trimester of pregnancy.

**References**

[1. Shea BJ, Reeves BC, Wells G, Thuku M, Hamel C, Moran J, et al. AMSTAR 2: a critical appraisal tool for systematic reviews that include randomised or non-randomised studies of healthcare interventions, or both. BMJ. 2017 Sep 21;358:j4008.](https://www.zotero.org/google-docs/?uTgHyy)

[2. Ottawa Hospital Research Institute [Internet]. [cited 2023 Apr 27]. Available from: https://www.ohri.ca/programs/clinical_epidemiology/oxford.asp](https://www.zotero.org/google-docs/?uTgHyy)

[3. Higgins JPT, Thompson SG, Deeks JJ, Altman DG. Measuring inconsistency in meta-analyses. BMJ. 2003 Sep 4;327(7414):557–60.](https://www.zotero.org/google-docs/?uTgHyy)

[4. Riley RD, Higgins JPT, Deeks JJ. Interpretation of random effects meta-analyses. BMJ. 2011 Feb 10;342:d549.](https://www.zotero.org/google-docs/?uTgHyy)

[5. Sterne JAC, Sutton AJ, Ioannidis JPA, Terrin N, Jones DR, Lau J, et al. Recommendations for examining and interpreting funnel plot asymmetry in meta-analyses of randomised controlled trials. BMJ. 2011 Jul 22;343:d4002.](https://www.zotero.org/google-docs/?uTgHyy)

[6. Dragioti E, Solmi M, Favaro A, Fusar-Poli P, Dazzan P, Thompson T, et al. Association of Antidepressant Use With Adverse Health Outcomes: A Systematic Umbrella Review. JAMA Psychiatry. 2019 Dec 1;76(12):1241–55.](https://www.zotero.org/google-docs/?uTgHyy)

[7. Dragioti E, Karathanos V, Gerdle B, Evangelou E. Does psychotherapy work? An umbrella review of meta-analyses of randomized controlled trials. Acta Psychiatr Scand. 2017 Sep;136(3):236–46.](https://www.zotero.org/google-docs/?uTgHyy)

[8. Stanley TD, Doucouliagos H, Ioannidis JPA, Carter EC. Detecting publication selection bias through excess statistical significance. Res Synth Methods. 2021 Nov;12(6):776–95.](https://www.zotero.org/google-docs/?uTgHyy)
